# Velocity-Based Movement Modeling for Individual and Population Level Inference

**DOI:** 10.1371/journal.pone.0022795

**Published:** 2011-08-11

**Authors:** Ephraim M. Hanks, Mevin B. Hooten, Devin S. Johnson, Jeremy T. Sterling

**Affiliations:** 1 Department of Statistics, Colorado State University, Fort Collins, Colorado, United States of America; 2 U.S. Geological Survey, Colorado Cooperative Fish and Wildlife Research Unit, Colorado State University, Fort Collins, Colorado, United States of America; 3 Department of Fish, Wildlife, and Conservation Biology, Colorado State University, Fort Collins, Colorado, United States of America; 4 National Marine Mammal Laboratory, Alaska Fisheries Sciences Center, National Marine Fisheries Service, National Oceanic and Atmospheric Administration, Seattle, Washington, United States of America; University of California, United States of America

## Abstract

Understanding animal movement and resource selection provides important information about the ecology of the animal, but an animal's movement and behavior are not typically constant in time. We present a velocity-based approach for modeling animal movement in space and time that allows for temporal heterogeneity in an animal's response to the environment, allows for temporal irregularity in telemetry data, and accounts for the uncertainty in the location information. Population-level inference on movement patterns and resource selection can then be made through cluster analysis of the parameters related to movement and behavior. We illustrate this approach through a study of northern fur seal (*Callorhinus ursinus*) movement in the Bering Sea, Alaska, USA. Results show sex differentiation, with female northern fur seals exhibiting stronger response to environmental variables.

## Introduction

The analysis of animal movement data can provide insight into the relationship between animal behavior and the heterogeneous environment they inhabit [1]–[3]. Recent advances in technology have made animal telemetry data easier to collect at finer temporal resolutions than previously possible [4], but there are significant challenges in linking telemetry data to animal movement and resource selection. Some challenges arise in the use of telemetry data, which are typically irregular in time, have measurement error that is varying in severity, and may exhibit temporal autocorrelation [4], [5]. Other challenges arise from modeling something as complex as animal movement, which typically exhibits changing behavior over time [2], [6]–[11] and may be driven by a mix of external (environmental) and internal (biotic) factors. Still other challenges involve making population-level inference based on telemetry data from multiple animals [12], [13].

Jonsen et al. [14] deal with temporal irregularity in telemetry data by explicitly building it into the likelihood portion of a Markovian state-space movement model. Johnson et al. [15] build on this idea in a continuous-time setting where directional persistence and velocity are modeled by an Ornstein-Uhlenbeck process. These continuous-time correlated random walk (CTCRW) models can then be used to make inference on velocity and movement parameters, as well as to characterize the posterior predictive distribution of the path of the animal. In the same spirit, Tremblay et al. [16] utilize a forward particle filtering method to create an ensemble of possible movement paths with regular temporal intervals, and Sumner et al. [17] utilize Bayesian methods to incorporate multiple data sources and prior information to obtain a posterior distribution of the animal's path [18]. Methods such as these are especially appealing because they provide information about the individual's location at any given time as well as the innate uncertainty associated with our knowledge of it.

State-space models are used extensively to model changing movement behavior over time [2], [6]–[11], [19]. Without accounting for heterogeneity in animal behavior over time, important drivers of movement could appear to be insignificant, due to the temporally changing nature of the animal's response. Consider, for example, a northern fur seal (*Callorhinus ursinus*). Northern fur seals are central place foragers during the summer months [20] and a typical summer movement path might exhibit loops in which the animal first travels away from the central location and then returns [16], [20]. If we did not consider the animal's changing behavior over time, and fit a statistical model to a movement path containing such loops, we might find no statistical relationship between the animal's movement and it's rookery, as the animal would spend large amounts of time moving both towards and away from the rookery. These contrasting behaviors (moving towards and away from the rookery) could effectively cancel each other out. If we instead fit a statistical model to a smaller segment of the movement path, containing only movement away from the rookery (or towards the rookery), the animal's directed movement behavior could be revealed.

Gurarie et al. [2] address this issue by utilizing a behavioral change point model to identify structural changes in an animal's movement. Their approach allows for the number and location of change points to be inferred from the telemetry data, but their model does not incorporate environmental effects; the inference on behavioral changes is based solely on the telemetry data. Polansky et al. [11] utilize Fourier and wavelet analysis to examine periodicity in movement behavior. Morales et al. [6] propose a model based on a mixture of random walks, though their analysis requires specification of the number of movement states an animal can exhibit, which may not be known beforehand. McClintock et al. [19] propose a method for incorporating an unknown number of random walks.

Telemetry data for different animals in a population often differ in length and occur on different spatial and temporal domains, but must be combined for population-level inference. Subsets of the population (e.g., genders or age classifications) may exhibit diverse movement patterns and responses to environmental drivers, but methods of testing for such differences are not well developed. Additionally, the relative ease with which telemetry data can now be collected makes the computational efficiency of an approach important as the number of movement paths grows and the temporal resolution of those paths increases. Hooten et al. [21] utilize a CTCRW model as the basis for an agent-based approach linking telemetry data to resource selection within a dynamic occupancy framework. The agent-based nature of this approach yields a framework for testing hypotheses related to animal movement and resource selection, but at a high computational cost, making population-level inference or inference about heterogeneous responses to the environment impractical.

Hooten et al [21] consider two classifications of environmental and biotic drivers of movement and resource selection: static drivers, which relate the absolute level of a covariate to movement behavior, and potential drivers, which relate local differences in the level of a covariate to movement behavior. Throughout this paper, we will use the term “potential” in this sense. Thus a “potential driver of movement” is a driver of movement based on the change of a covariate, and not a “hypothesized driver of movement”. To illustrate the distinction between static and potential drivers of movement, and the assumptions associated with each, consider a continuous covariate like elevation. Modeling movement behavior as a function of elevation (static driver) assumes that animals move differently at lower elevations than they would at higher elevations. On the other hand, modeling movement behavior as a function of the change in elevation (potential driver) assumes that animal movement is related to the gradient, or directional derivative, of elevation.

As another example, consider the movement of a marine animal in relation to sea surface temperature (SST). Modeling the gradient of SST as a potential driver of animal movement could provide inference about whether the animal is moving predominantly toward colder (or warmer) waters. If, instead, we modeled SST as a static driver of movement, we would be investigating whether an animal moves differently in colder water than it does in warmer water.

We propose an approach to linking telemetry data to potential drivers of animal movement. This approach utilizes the CTCRW model of Johnson et al. [15] to stochastically interpolate temporally-irregular telemetry data to regular time points on a scale comparable with environmental covariate effects. A velocity-vector-based model allows for inference to be made about potential drivers of movement within a framework that is intuitive and computationally efficient. A change point model allows for temporal heterogeneity in these velocity-based drivers of movement, and an unknown (random) number of change points can be accommodated through use of birth-death Markov chain Monte Carlo (BDMCMC) methods [22]. This approach allows for familiar methods of model comparison and selection. Finally, cluster analysis of movement parameters allows for inference to be made on heterogeneous and complex population-level movement patterns.

In what follows, we first present the statistical framework underlying our approach, then illustrate the approach by analyzing the movement paths of 45 northern fur seals, and present results of this study. We conclude with a discussion of the velocity-based framework for animal movement and resource selection.

## Methods

### Continuous Statistical Model for Telemetry Data

Assume that we have telemetry location data for an individual animal at times 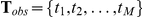. Let 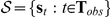 be the set of observed locations associated with the animal at all observation time. Often, these measurements do not come at regular temporal intervals, as missing data are common. Johnson et al. [15] provide a means for making inference about the continuous-time movement path of the animal, given such telemetry data. In practice, this approach allows for inference about the location and directional velocity of the animal at an arbitrarily fine temporal resolution at regular temporal intervals. In particular, Johnson et al. [18] provide a means for finding the posterior distribution 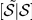, where 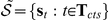 is the quasi-continuous path of the animal at an arbitrarily fine temporal resolution 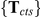, and the bracket notation ‘
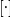
’ denotes a probability distribution.

**Figure 1:**
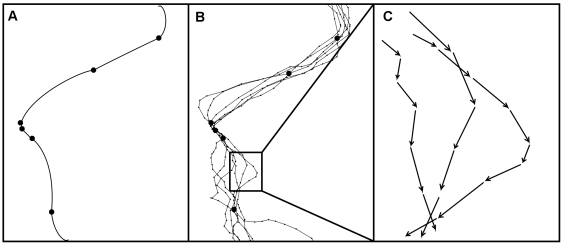
Overview of velocity-based movement modeling framework. Animal telemetry data (a) are collected at temporally irregular intervals. A continuous time correlated random walk model (b) is used to create a posterior predictive distribution of paths at temporally regular intervals with high temporal resolution. Path realizations from the posterior predictive distribution are transformed to velocity vectors at temporally regular intervals (c). These velocity vectors allow for efficient modeling of environmental and biotic drivers of animal movement.

There are advantages to conditioning on 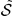, as opposed to 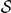 directly, to make inference about movement and resource selection. The locations in 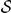 are typically temporally irregular, since telemetry data are typically collected at irregular intervals. Lagrangian (individual-based) models of movement often use the movement step lengths between locations 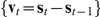 instead of the actual telemetry locations 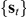 [23], but the movement steps between successive observations that are close temporally are likely to be quite different than the movement steps between successive observations that are distant temporally. By conditioning on the continuous path 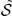, we can obtain values of 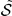 at temporally regular intervals, providing equal weights to all movement steps. At the same time, the distribution of 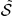 accounts for the uncertainty in the location and directional velocity at any given time. Thus, times in 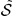 that are close to times for which we have telemetry observations in 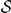 will have less uncertainty in the distribution of location and velocity than will times that are temporally distant from any telemetry observations (Figure 1). The ability to sample realizations from 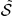 at arbitrary temporal resolution also allows us to use a temporal resolution that matches the resolution of available environmental covariates.

Our strategy is to utilize realizations from the continuous posterior path distribution 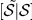 to make inference about environmental and biotic drivers of animal movement and resource selection. In effect, this amounts to a model-based augmentation of the telemetry data 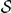. This approach is similar to that of multiple imputation [24], [25], in which missing values are imputed by multiple draws from the posterior predictive distribution of the missing data, and inference is made by averaging over the results obtained by each of these draws. The approach that we take here, which was first described in the movement context by Hooten et al. [21], is to make inference about a set of parameters 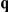 related to drivers of movement by first proposing a statistical model (i.e., likelihood) for the relationship between the movement path and the environmental drivers of movement:
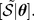
(1)Specifying prior distributions for 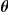 allows us to find the posterior distribution:
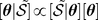
(2)and inference about 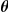, conditioned only on 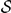, can be accomplished by integrating over the distribution of 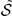:
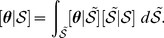
(3)Within a Bayesian hierarchical modeling (BHM) framework, we can easily accomplish this integration through composition sampling.

Hooten et al. [21] employed a dynamic occupancy model within an agent-based framework for 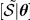. This framework is flexible and intuitive, but computationally demanding. At this time, the computational costs of this agent-based approach preclude more complicated modeling considerations, such as multiple animals or multiple movement profiles in one path. We thus propose an alternative approach for linking potential drivers of movement to 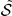 that is similar in spirit, but based on velocity rather than occupancy.

### Velocity-Based Movement Model

Let 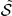 be a continuous representation of an animal's true, but unknown, continuous movement path. A simple transformation, 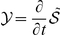, yields the animal's continuous time-varying velocity vector, which specifies the animal's speed and direction at any time. Given a starting location 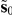, this transformation is invertible and preserves all information present in the movement path 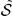. In practice we will not have a truly continuous movement path, but instead a quasi-continuous set of location information 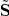 at temporally regular intervals 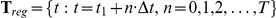. Thus, 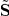 would take the form of a 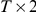 matrix of locations at temporally regular intervals along an animal's movement path, with 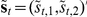, and 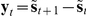 yields the animal's velocity vector at times 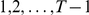, which can be collected in a 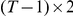 matrix 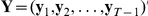. Thus the 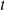-th row of 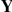 contains the 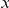 and 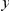 coordinate of the animal's velocity vector specifying the animal's speed and direction at time 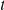. The computationally discrete form for the transformation from location to velocity is essentially a first-differencing, as is common in the analysis of time series data [26] and animal movement [23]. As the temporal resolution 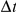 of the discrete form 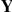 approaches zero, the limit is the continuous velocity vector 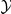.

We now propose a statistical model for 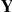, conditioned on parameters (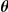) related to potential drivers of movement and the movement path 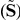. Environmental data are commonly available in gridded form (e.g., pixels). As our response variable, 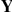, is in velocity vector form, we propose to utilize the gradient of environmental variables as covariates in a statistical model [21], [27]. The gradient of a spatially-referenced covariate is a vector field that points in the direction of the greatest rate of increase in the covariate. If there are 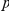 environmental or biotic drivers of movement of interest, let 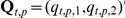 be the vectorized gradient of the 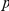-th environmental variable at the location 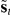, and let 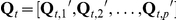. If 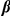 is a 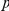-vector of regression coefficients related to the 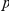 environmental (and other) covariates whose gradients are in 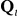, then we could then specify a multivariate regression model for the relationship between the velocity vector 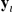 and the environmental and biotic covariates in 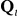:
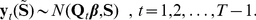
(4)Here 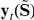 is normally-distributed with mean 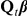 (matrix multiplication) and variance 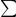.

Modeling the relationship between velocity and covariate gradients in this way attempts to capture an animal's response to changes in the covariates (potential drivers of movement in [21]), as opposed to the animal's response to a static level of the covariates (static drivers of movement in [21]). For example, a positive 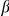 in (4) related to elevation would indicate that the animal moves generally in the direction of increasing elevation, perhaps indicating a migration to higher elevations, a negative 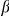 would indicate that the animal is moving to lower elevations, and a 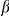 near zero would indicate that the animal's movement is not correlated with increasing or decreasing elevation.

Environmental data often come in categorical form, which can be incorporated into models as static drivers of movement with the assumption that animal movement and behavior differs between categories. However, the velocity-based framework (4) incorporates potential drivers of movement (gradients) and not static drivers. To illustrate one approach for utilizing a categorical variable in the existing velocity-based framework, consider a categorical variable consisting of 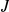 land cover types. For each cover type 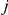, a new spatial variable could be created consisting of the shortest distance from each spatial location to the 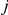-th cover type. The gradients of these 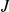 new variables would point in the direction of the shortest direct path to each cover type, allowing inference to be made in the velocity-based framework about cover types that animals are drawn to, or away from.

### Temporal Heterogeneity in Drivers of Movement

It is often unreasonable to assume that the animal's response to the environment remains homogeneous over the temporal domain of 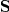. We propose an approach for modeling heterogeneity in response to potential drivers of movement that is based on partitioning the temporal domain of the movement path into 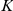 regions in which the animal's response is homogeneous. This allows the effect of an environmental driver of movement to vary over time, and also allows inference on the degree of heterogeneity in the animal's response to the environment. The number of change points, 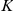, can be either fixed *a priori* or random and unknown, as explained below.

Let 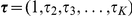 be a 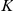-vector with each entry 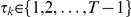 being the change point between two partitions in which the animal exhibits distinct movement profiles. Then we can write (4) as:
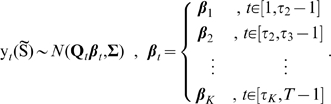
(5)In (5), we model changing patterns of movement and resource selection as coming from a bivariate normal distribution with a mean function 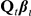 that is piece-wise linear. Each partition of the movement path represents a contiguous segment of time in which the animal's pattern of movement and selection is homogeneous. Thus 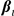, the response to the drivers of movement represented by covariate gradients in 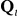 is allowed to vary over time, taking different values at each partition of the movement path 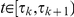. We will present an approach that allows inference to be made on the number of partitions 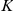 in an observed movement path, their temporal locations 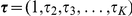, and the response to drivers of movement associated with each partition 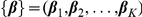. Allowing the length, location, and number of the partitions to change results in a highly flexible model of heterogeneous response to the environment. In this way, we can make inference about changing behavior over time in individual animal movement and selection.

### Univariate Formulation

The velocity-based model of animal movement and selection we have presented (5) is bivariate. The dependent variable is the vector-valued velocity 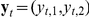 of the animal at time 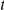, where 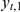 and 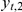 are the components of the vector-valued velocity in two orthogonal directions (e.g., Easting and Northing). The independent variables are also vector-valued:
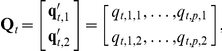
where 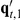 and 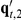 are the components of the 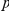 covariate gradients in the same two orthogonal directions.

In a movement setting, it is reasonable to assume that 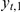 and 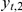 are uncorrelated, conditional on 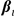. If this were not true, and 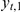 and 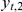 were correlated, the animal would prefer moving in northeast or southwest directions (in the case of positive correlation) or southeast or northwest directions (in the case of negative correlation) over other directions. Drift behavior of this form, if present, could be modeled in the first-order effects 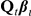. The assumption of independence of 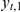 and 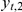 allows us to formulate the bivariate model (5) in an equivalent univariate regression form:
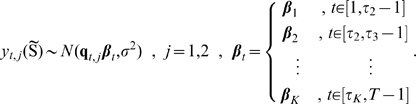
(6)This model (6) specifies the relationship between the components of an animal's velocity vector over time 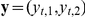 and the parameters related to drivers of movement 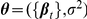 in a linear framework that is extensible, allows for easily interpretable results, and is computationally efficient.

### Prior Distributions for Model Parameters

We will fit this model using a Bayesian hierarchical modeling (BHM) framework [28] and thus specify prior distributions for all model parameters. For the regression coefficients 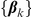, we specify a Gaussian prior distribution with mean 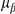 and variance-covariance matrix 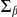.
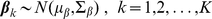
(7)In practice, setting 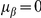 and 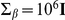 gives a diffuse prior that allows inference to be driven in large part by the data.

For the variance component in the regression model (5), we specify an inverse-gamma prior distribution with shape parameter 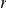 and scale parameter 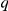:
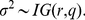
(8)In our analysis, we have set 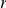 and 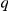 in such a way that the prior mean of 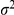 is 10 and the prior variance is 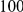, giving a diffuse prior distribution.

For the locations of the change points 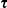, we specify a discrete uniform prior distribution, with change points equally likely to occur at any point in the observation window 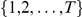:
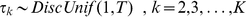
(9)subject to the constraints that 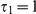 and 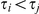 for each 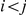.

If we have fixed 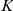 *a priori*, then (6)–(9) is a fully specified BHM. If we allow for an unspecified number of change points, and thus movement partitions, we allow 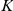 to be random as well, and assume a Poisson prior distribution:
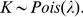
(10)


The choice of 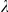, the prior mean number of behavioral partitions of the movement path, should be made based on existing knowledge of the animal, the length of the observation period, and the time scale at which inference on changing behavior patterns is desired.

### Model Implementation

Having specified a statistical model, we now outline the procedure used to fit this model to telemetry data. Markov chain Monte Carlo (MCMC) methods are used extensively to fit BHMs such as we have specified in (6)–(10) by sampling from the posterior distribution of the parameters [29]. In this case, full-conditional distributions can be found for 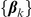 and for 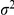, allowing use of the robust Gibbs sampler within an MCMC framework [29]. Updates to 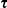 are accomplished through Metropolis-Hastings steps in the algorithm.

Allowing 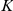 to vary results in a parameter space that varies as samples are drawn from the posterior distribution with varying numbers of change points, and thus varying the dimensionality of model parameters 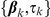. That is, we do not specify beforehand how to partition the movement path into contiguous segments in which the animal's pattern of movement and response to the environment is homogeneous. Rather, we have specified a BHM (6)–(10) so that location data can be used to infer the number and location of these partitions. MCMC methods employ random walks through parameter space to sample correlated realizations from the distribution of interest. In the case of 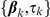, the MCMC approach will lead to different iterations having different numbers of partitions 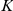. As each of the 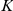 partitions has a distinct pattern 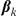 of response to environmental and biotic covariates, changing the number of partitions results in adding or deleting the set of parameters 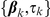 describing that partition from the model.

Reversible jump Markov chain Monte Carlo (RJMCMC) methods are one popular approach for sampling from the appropriate stationary distribution in a situation where the parameter space varies [30], but the rate of convergence in the algorithm is highly dependent on the choice of proposal distribution. Birth-death Markov chain Monte Carlo methods (BDMCMC) are continuous-time MCMC methods that are closely related to RJMCMC [22], [31]. One particular advantage of BDMCMC is the ease with which multiple proposal distributions (e.g., birth distributions) can be implemented [22]. In the BDMCMC approach, the parameter space of the change-point model (6)–(10) is viewed as a point process in 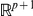 in which each point is a vector: 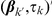 containing model parameters for one movement partition. New movement partitions are added to the model space in a manner specified by a birth distribution, 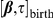. Each existing movement partition is allowed to die (be removed from the point process and thus the model) at a Poisson rate specified in such a way that the BDMCMC process is guaranteed to converge to the appropriate stationary distribution [22], [31]. Success of the BDMCMC procedure is dependent on finding a birth distribution that allows for good mixing and thus rapid convergence to the appropriate stationary posterior distribution. In general, BDMCMC is merely an efficient algorithm for implementing the change point model (7). We provide further discussion of the BDMCMC process in the supporting information (Text S1).

The hierarchical model in (6)–(10) is also conditioned on the true quasi-continuous movement path 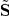. 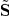 is unknown, and to make inference on 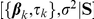, as opposed to 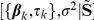, we need to integrate over the distribution of 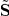. This is accomplished through composition sampling; BDMCMC updates of 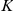, 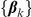 , and 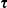 are drawn iteratively with Gibbs updates of 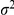, 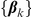 , and 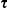 to improve mixing. In brief, the algorithm for drawing samples from the posterior distribution 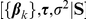 in the case where 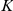 is treated as random and unknown is as follows:

1. Specify a beginning model state with 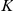 partitions.
2. Sample 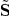 from 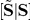.
3. Update 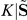 and 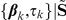 using the BDMCMC procedure.
4. Sample 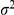 from its full-conditional distribution.
5. Sample 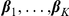 from their full-conditional distributions.
6. Update 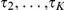 using Metropolis-Hastings steps.
7. Repeat steps 2–6 until convergence is reached and a sufficient number of samples are obtained.

The algorithm for the fixed-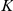 case is identical to the variable-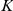 case, except that step 3 is omitted.

It should be noted that steps 5–6 in the algorithm are not strictly necessary for convergence to the appropriate posterior distribution as the BDMCMC process updates 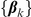 and 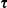 in step 3. However, including steps 5–6 greatly improves the mixing and thus computational efficiency of this iterative sampling process.

The supporting information contains example code (Text S1) for implementing this approach and a simulation study (Text S2) illustrating the implementation of this individual-level model of animal movement on simulated data.tpb

### Model Comparison and Selection

In regression models, such as (6), parsimony in covariates is often desirable. Multiple methods for comparing models with different sets of regression covariates exist, including the Akaike information criterion (AIC) [32]. For Bayesian models, the Bayesian information criterion (BIC) [33], and the deviance information criterion (DIC) [34] are commonly used. Celeux et al. [35] present eight methods for calculating DIC for models with missing data. These methods are easily applied to the model-based data augmentation approach which we present here. Celeux et al. [35] suggest that the most reliable of the eight methods they present is
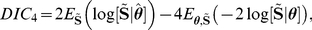
(11)where 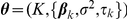, the full set of parameters in (6)–(10), and 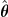 is the posterior mean of these parameters.

This criterion can be used to compare models with different subsets of covariates, which may aid inference on the relative importance of individual environmental covariates to animal movement. This criterion could also be used for multi-model inference [36].

### Population-Level Inference

Telemetry data for different animals in a population often differ in length and occur on different spatial and temporal domains. The differences in observation windows among animals can make scaling up inference to a population level difficult. One significant advantage of the continuous movement model for animal movement we propose is that a uniform temporal discretization of the movement path can be obtained for all animals. Population-level inference can then be made by aggregating inference from different animals in covariate effect space.

State-switching models of animal movement commonly assume that a population's movement can be described by a fixed number of population-level movement regimes [6], [14], each representing a distinct form of movement. For example, one regime could represent directed, migration-like movement, another regime could represent a movement state of strong response to certain environmental factors, and another regime could represent a state of little movement.

In our individual approach to modeling animal movement, we allowed for temporal heterogeneity in the response to environmental and biotic drivers of movement through a change point model (6)–(10). At a population level, we also adopt a framework that allows for different states of movement and behavior, with the following assumptions:

1. At the population level, patterns of animal movement and response to the environment can be partitioned into a fixed number of regimes, each of which can be represented by a distinct pattern of correlation to gradients of environmental or biotic covariates (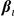 in our individual-level model).
2. Sub-groups of the population may have different propensities for each of these movement regimes, manifested by differences in the proportion of use of the regimes across subgroups.
3. Individual animals may have varying propensities for each of the movement regimes as well, manifested by differences in the proportion of use of the regimes across individuals.
4. Individual animal movement in an observation period can be modeled by breaking the path into an unknown (random) number of partitions, as specified in our individual-level approach (6)–(10). In each of these partitions, animal movement and resource selection follows one of the population-level regimes.

**Figure 2:**
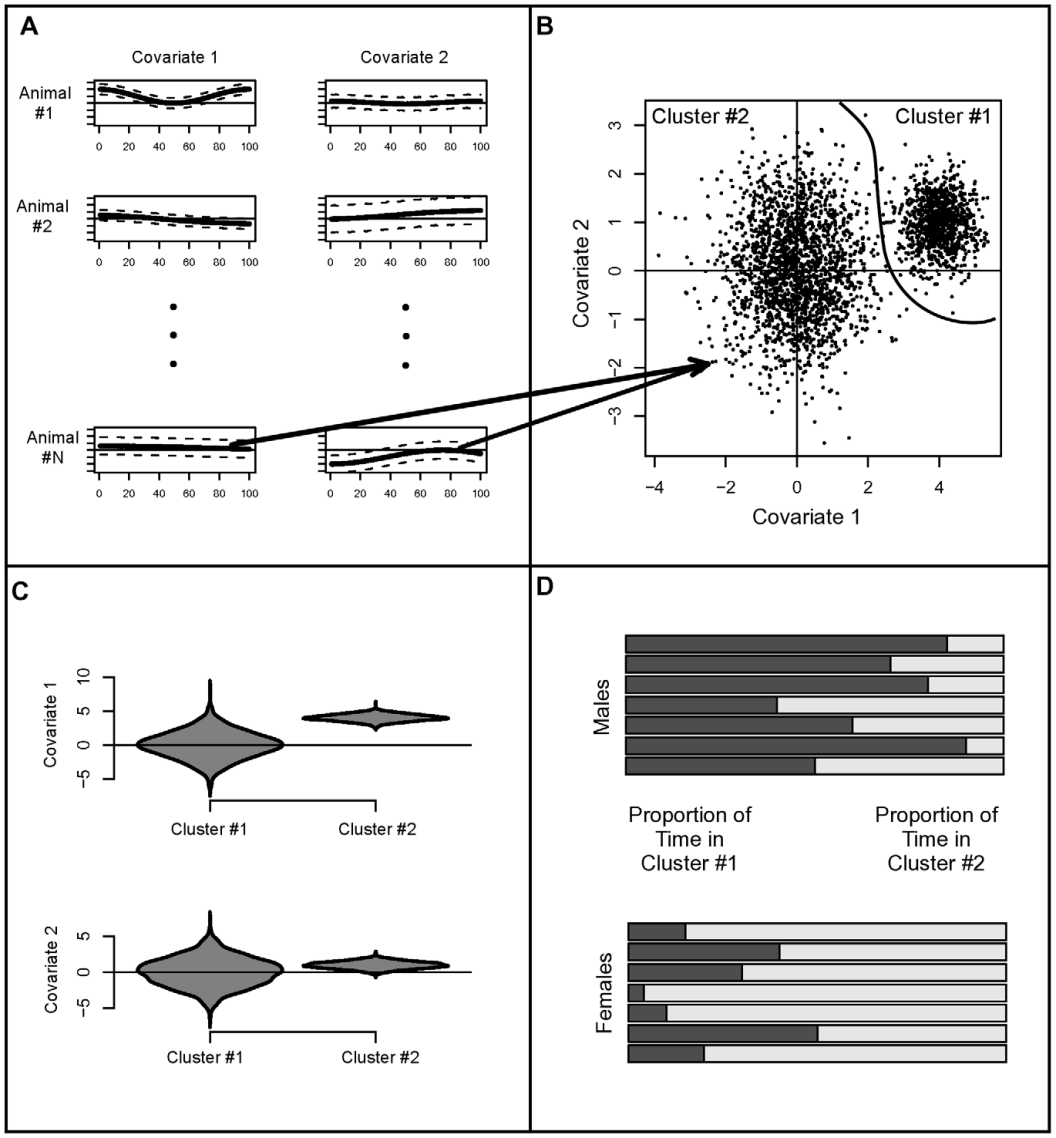
Overview of population-level inference based on a cluster analysis in the parameter space. We show here an overview, using simulated data, of the approach for making population-level inference based on the results of individual-level response to covariates over time. Inference is made about the time-varying response 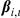 of multiple animals to environmental drivers (a), with each animal being analyzed individually. In (b), the mean values of 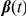 for all members of the population and all times are combined. Inference about population-level drivers of movement can be made using cluster analysis. Examination of the marginal distributions of each multivariate cluster (c) can aid understanding of the movement profile defined by each cluster. Differences between subsets of the population can be inferred from differences in the proportion of time each subset spends in each movement cluster (d).

One approach to making population-level inference together with individual-level inference would be to add another level to the hierarchical model (6)–(10). The prior on 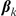 (8) could be replaced with a hierarchical prior, such as a mixture of normal distributions, with each mixture component representing one population-level movement regime. However, such an approach is currently precluded by computational barriers. Inference in such a unified hierarchical model would require jointly making inference on an individual-level model for each animal in the population, and tuning such a model to ensure efficient mixing of the MCMC algorithm could be infeasible. Instead, we propose an approach for making population-level inference about movement and resource selection based on a post-hoc analysis of the inference made on individual movement paths from all members of the population. We have outlined this approach graphically in Figure 2.

Our individual-level model, (6)–(10), results in inference about the time-varying response of the animal to different environmental and ecological drivers, 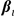, where 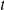 indexes time. If we apply the approach to multiple animals in a population, then the results for the 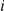-th animal are 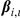 (Figure 2a), where 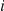 indexes animals and 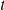 indexes time. Aggregating the values of 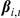 for all animals and times in parameter space (Figure 2b) can reveal population-level responses to drivers of movement. Cluster analysis [37] can be used to reveal these profiles, which represent population-level movement states. As clustering is an unsupervised form of learning, interpreting the differences between different movement clusters can be done by examining the marginal distribution of the response to each driver of movement (Figure 2c). For example, a migration to lower elevations would manifest itself as a cluster of 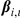 with negative values for the element of 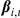 associated with elevation. Foraging behavior, in which an animal's movement is not driven by environmental factors, could manifest itself as a cluster of 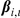 near 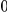, while a state of high speed movement would manifest itself as a cluster with extreme absolute values of the 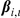. After clustering the 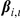 in parameter space, differences in movement between subsets of the population would manifest as differences in the proportion of time spent in each of the clusters by members of different subsets (Figure 2d). In this way, location data from multiple animals can be combined to make population-level inference about movement and resource selection.

Our simulation study (Text S2) illustrates this population-level approach based on simulated individual-level movement.

### Application: Northern Fur Seal Movement

We apply our approach for individual and population-level inference to a study of northern fur seal (NFS) movement and resource selection. Approval for the field research described below was granted by permission of the National Marine Fisheries Service, permit number 782–1455.

Northern fur seals are pelagic foragers found in the North Pacific Ocean and Bering Sea. During the winter months, NFS are migratory, while during the summer months, a large percentage of the NFS population congregate at the Pribilof Islands (St. Paul and St. George Islands, Alaska, USA) to breed [38], [39]. Female NFS, after giving birth to a pup, nurse their dependent offspring for around 4 months by alternating trips to sea to feed with time on shore to nurse. Male NFS, on the other hand, do not participate in pup rearing.

**Figure 3:**
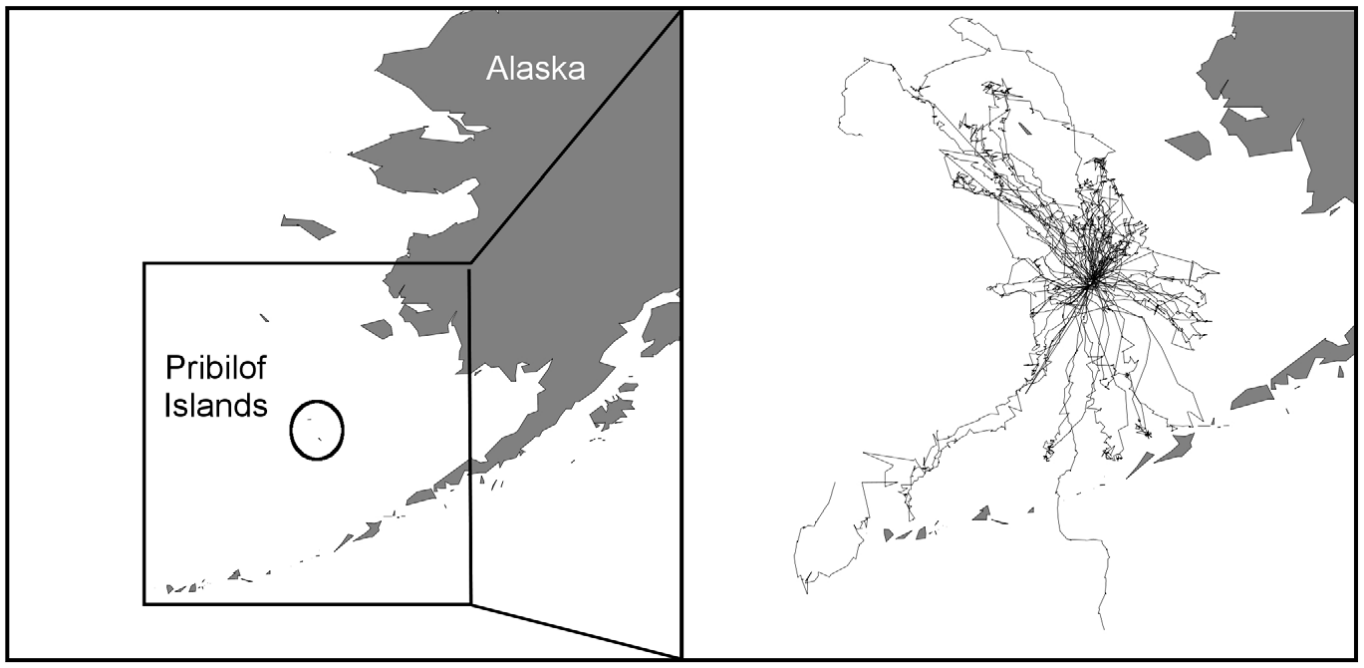
Movement paths for northern fur seals in the Pacific Ocean. In 1999 and 2000, 45 northern fur seals on the Pribilof Islands were fitted with satellite transmitters. Movement paths for all 45 northern fur seals are shown.

During the 1999 and 2000 breeding seasons, 45 northern fur seals were captured at two haul-out sites and two rookeries on St. Paul Island. In 1999, thirteen juvenile male NFS were captured, and in 2000, fifteen juvenile male NFS and seventeen lactating female NFS were captured. These animals were fitted with satellite transmitters, and location information on foraging trips was obtained using the Argos system (Argos website (accessed 2011) https://www.argos-system.org/manual/). During the summer months, in which our study was conducted, NFS are central place foragers. Figure 3 shows a compilation of the movement paths from these animals. Sea surface temperatures were colder in 1999 than in 2000, and this may lead to different NFS movement patterns in these two years.

We were most interested in at-sea behavior of NFS, and so we separated the full location record for each animal in the study into separate at-sea trips by removing locations at times when the NFS was hauled out one one of the Pribilof islands. For 34 of the NFS, this involved trimming hauled-out telemetry locations from the beginning and end of the observation window, resulting in one at-sea trip for each animal. For the remaining 11 NFS, we split the full location record into two or more at-sea trips beginning (and ending for complete trips) with a single on-land location. All told, the telemetry locations for the 45 NFS were split into 64 at-sea trips. The number of at-sea trips for each animal is shown in the supplemental material (Text S3).

We used three ocean environmental covariates for this study: sea surface temperature (SST), Chlorophyll *a* level (CHA), and net primary production (NPP). Each of these covariates were measured monthly at a 9 km resolution across the Bering Sea. For each at-sea trip, the mean observation time of the path was found, and the measured covariates from the month before and the month after the mean observation time were averaged, with weights inversely proportional to the time between the mean observation time and the monthly covariate observation times. This resulted in a spatially referenced set of covariates for each at-sea path. Missing values in the covariate data were interpolated using thin plate splines [40] as implemented in the fields package [41] in the R statistical computing environment [42]. For each covariate, the gradient (directional derivative) was calculated and used as a potential driver of movement.

Female NFS are central-place foragers during the summer months, often making trips of 4–10 days at a time to travel to foraging locations. As they provide the only source of nourishment for their pups, the length of a foraging trip is constrained by the amount of time their pups can survive without food [20]. In an attempt to include this potential driver of movement in our study, we include the gradient of the distance to the seal's rookery in the set of model covariates. This is done for both male and female NFS.

We use the velocity-based approach described in the preceding sections to examine telemetry data from each of the 64 at-sea trips made by these NFS. We then make population-level inference based on the individual-level results for 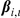 for all animals. The aim of our study is to characterize individual seal response to environmental drivers of movement and make inference about potential differences between subsets of the population. In particular, our population-level study focuses on two questions. We first compare movement patterns in male NFS tagged in 1999 to movement patterns of male NFS tagged in 2000. We then examine potential differences between juvenile male NFS and lactating female NFS movement and response to environmental factors.

### Individual-Level Methods

A preliminary model fit with a fixed number of change-points (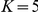) was conducted on each animal to provide reasonable birth distributions. Following [22], we utilize a birth distribution based on the posterior distribution of 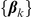 from these preliminary models. In the full models, in which 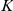 is allowed to vary, when a new partition 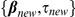 is added to the parameter space via the BDMCMC process, it is drawn from the following birth distribution: we first draw 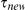, the change-point for the new partition, uniformly from all times at which there is not currently a change-point:
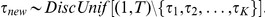
We then draw model parameters for the new partition from a Gaussian distribution:
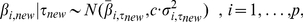
(12)where 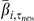 is the posterior mean value at time 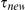 for 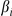 from the preliminary fixed-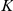 model fit, 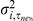 is the posterior variance at time 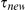 of 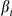 in the fixed-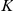 model fit at time 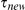, and 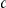 is a tuning parameter which can be modified to improve mixing. We note that a birth distribution in BDMCMC is similar to a proposal distribution in a Metropolis-Hastings update in that the posterior distribution is invariant to the choice of proposal (or birth) distribution, though the rate of convergence can vary widely for different proposal (or birth) distributions. Our use of the data to inform a birth distribution only facilitates mixing in the MCMC process, and does not influence the resulting posterior distribution (see [22] for further discussion). The supporting information (Text S1) contains more details of the BDMCMC algorithm.

We analyzed each of the 64 at-sea paths from the 45 NFS using the individual velocity-based movement approach. The algorithm for fitting the continuous-time correlated random walk model [15] did not converge for five seal paths (see Text S3 for details). For each of the remaining 59 at-sea paths we used a preliminary model with a fixed number of change-points to generate a birth distribution. The birth distribution was then used in the variable-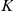 model. The variable-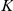 algorithm was used to generate 11,000 MCMC iterations, with the first 1,000 being discarded as burn-in. Convergence of the Markov chain for each animal was assessed visually by examining the chains for proper mixing. Example trace plots are shown in the supporting information (Text S3). For animals whose Markov chains showed lack of convergence, an additional 100,000 iterations were generated. MCMC algorithms for five NFS paths did not converge after this longer run, and these paths were discarded from the analysis, leaving 54 at-sea paths from 41 animals in the study (see Text S3 for details). Our population-level study is based on the individual results from these 41 animals.

We also conducted an in-depth individual-level study on two NFS, one male (animal 1) and one female (animal 2) to illustrate model comparison procedures. For each of these animals, a set of models with all possible subsets of the four covariates were applied to the data. The MCMC procedure was used twice with unique starting values to produce 11,000 iterations of the composition sampler for each model and animal, and the first 1,000 iterations were discarded as burn-in. Convergence was assessed visually (see Text S3 for additional details). The two chains for each model were combined, resulting in 20,000 iterations for each model and animal. The 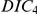 for each model was computed, using the 20,000 iterations.

### Population-Level Methods

Our population-level inference is based on clustering the time-varying coefficients 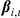 for the 41 animals in the individual-level study. To remove the temporal autocorrelation in the response to the drivers of movement, and to make the cluster analysis more tractable, we thinned the 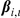 for each path, keeping only 1/100 of the time points. The resulting values of 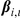 from all animals were combined, and a Gaussian mixture model clustering algorithm was fit using the MCLUST R-package [43]. Models with one to eight mixture components were compared using BIC, and the best model was used for inference on population-level drivers of NFS movement.

Differences in movement by subsets of the NFS population were assessed by examining differences in the time spent by animals in the different subsets (e.g., male and female NFS) in each of the resulting movement states. As the location data vary in length across animals, we examined differences in the proportion of time each animal spent in each movement cluster. If an animal's telemetry location data were split into multiple at-sea trips, all trips were aggregated by animal and the proportion of time spent in each movement cluster for the animal was the proportion of all at-sea time. In this way we can examine differences in the proportional use of the different movement clusters between individuals and subgroups of the population.

To test for differences between male and female NFS movement patterns, we applied a classification tree [44] to the proportion data with sex as a response variable. A classification tree was also used to test for differences between movement patterns of males tagged in 1999 and males tagged in 2000.

## Results

**Figure 4:**
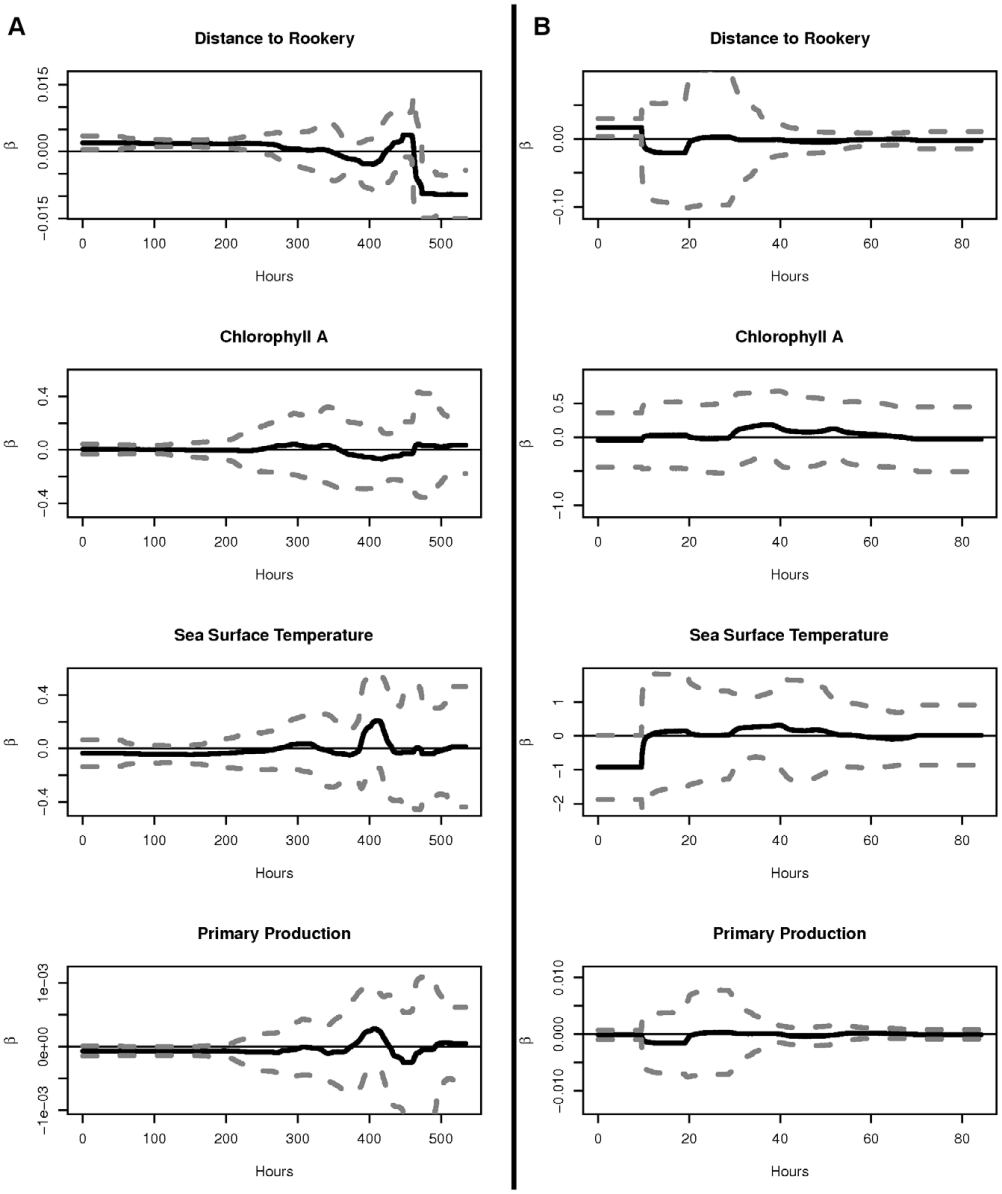
Time-varying response to the environment. The time-varying posterior mean (solid black line) and 95% credible interval (dashed line) for 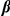 for (a) animal 1, a male northern fur seal, and (b) animal 2, a female northern fur seal are shown. The change-point model used allows for heterogeneous response to environmental and biotic drivers of movement.

The computed DIC values for each of the fifteen models for animal 1 and animal 2 are shown in Table 1. Figure 4 shows the posterior distributions of the 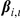 over time in the full model (model 1) for animal 1 (Figure 4a) and animal 2 (Figure 4b). Similar plots for the 39 other animals in the study are shown in the supporting information (Text S3).

**Table 1:** Comparison of models for animal 1, a juvenile male northern fur seal, and animal 2, a female northern fur seal, using DIC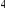.

Model Index	Distance to Rookery	Chlorophyll A	Sea Surface Temperature	Primary Production	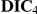 Animal 1	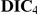 - Animal 2
1	X	X	X	X	−69752	−8836
2		X	X	X	−69471	−8852
3	X		X	X	−69860	−8855
4	X	X		X	−69794	−8867
5	X	X	X		−69825	−8862
6			X	X	−69538	−8869
7		X		X	−69397	−8877
8	X			X	−69788	−8883
9		X	X		−69539	−8877
10	X		X		−69950	−8874
11	X	X			−69775	−8889
12				X	−69487	−8885
13			X		−69565	−8879
14		X			−69496	−8892
15	X				**−69961**	**−8897**

An “X” shows that the covariate is included in the model.

**Figure 5:**
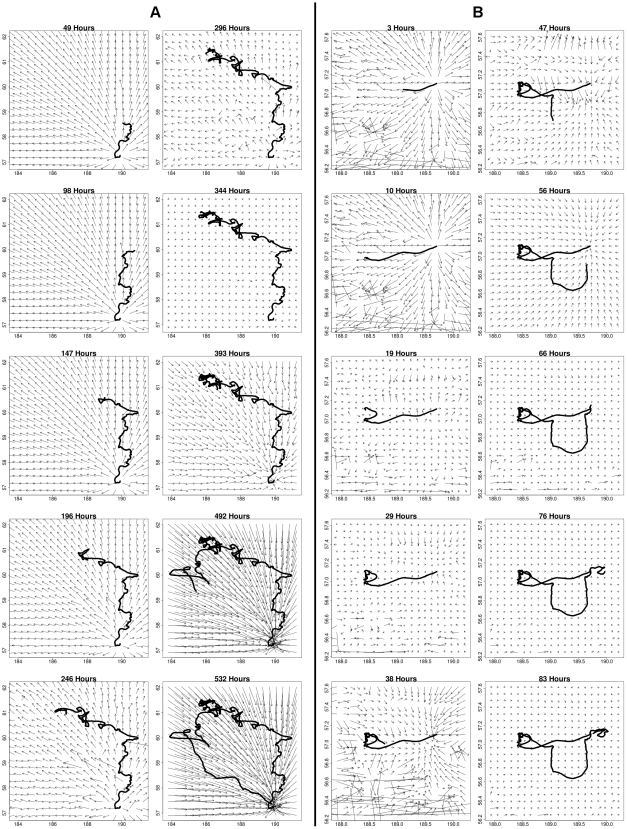
Gradient field of combined drivers of movement over time. The effect of environmental and biotic drivers of movement can be thought of as a bumpy flow surface on which the animal tends to move in the direction of greatest local descent. The gradient fields for (a) animal 1, a male fur seal, and (b) animal 2, a female fur seal, exhibit significant change over time.

The combined effect of all covariates can be thought of as an irregular flow surface on which the animal tends to travel in the direction of steepest descent. This flow surface changes over time, and Figure 5a shows snapshots in time of the net gradient field of the four drivers of movement on animal 1. Figure 5b shows corresponding gradient fields for animal 2.

**Figure 6:**
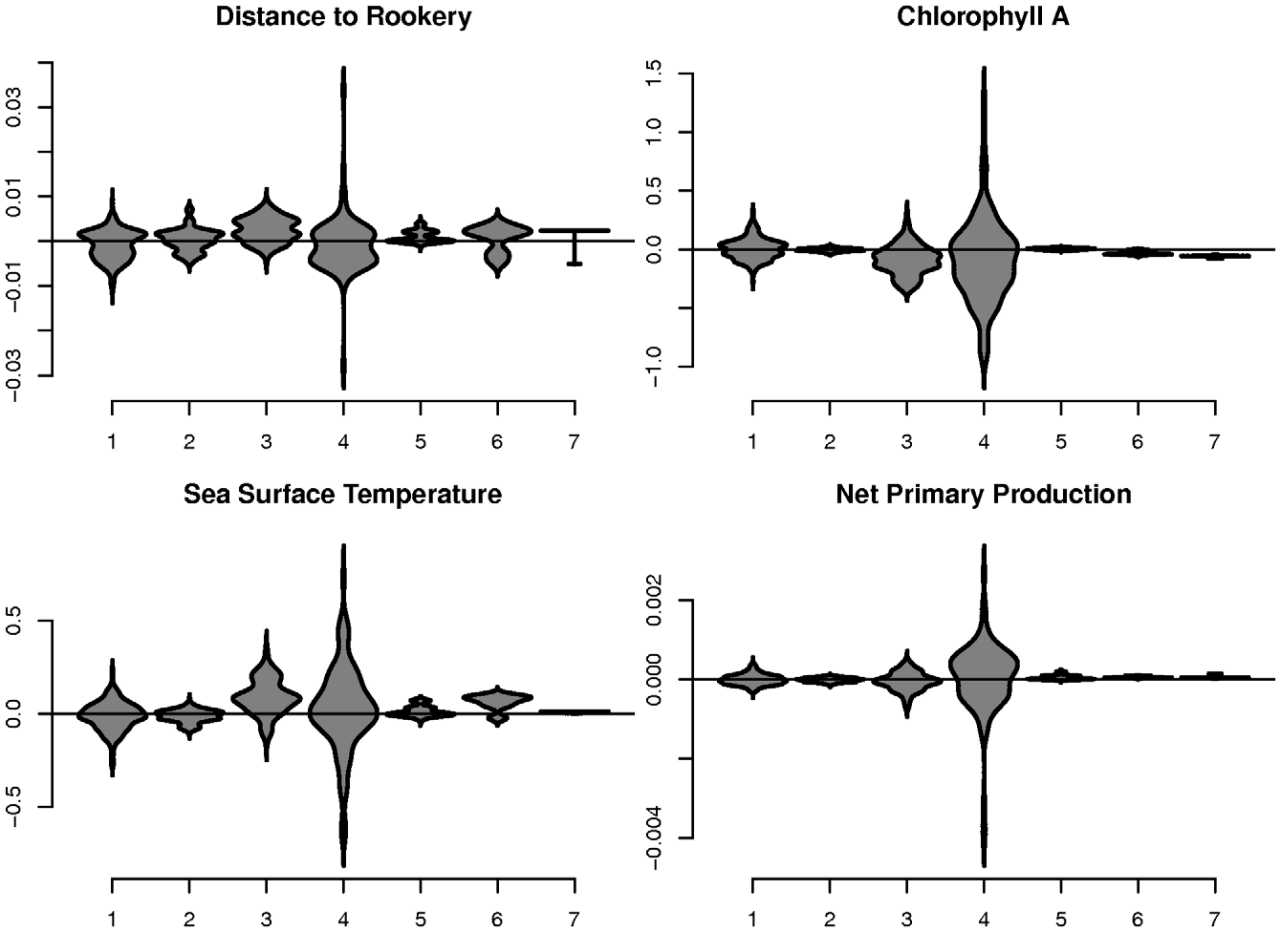
Population-level movement clusters. Violin plots of values of 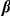 for each cluster of NFS movement. Male NFS spend proportionately more time in cluster 5, a movement state of low response to environmental drivers, than do female NFS. Female NFS spend proportionately more time in cluster 4, a movement state of high absolute speed and response to environmental drivers.

**Figure 7:**
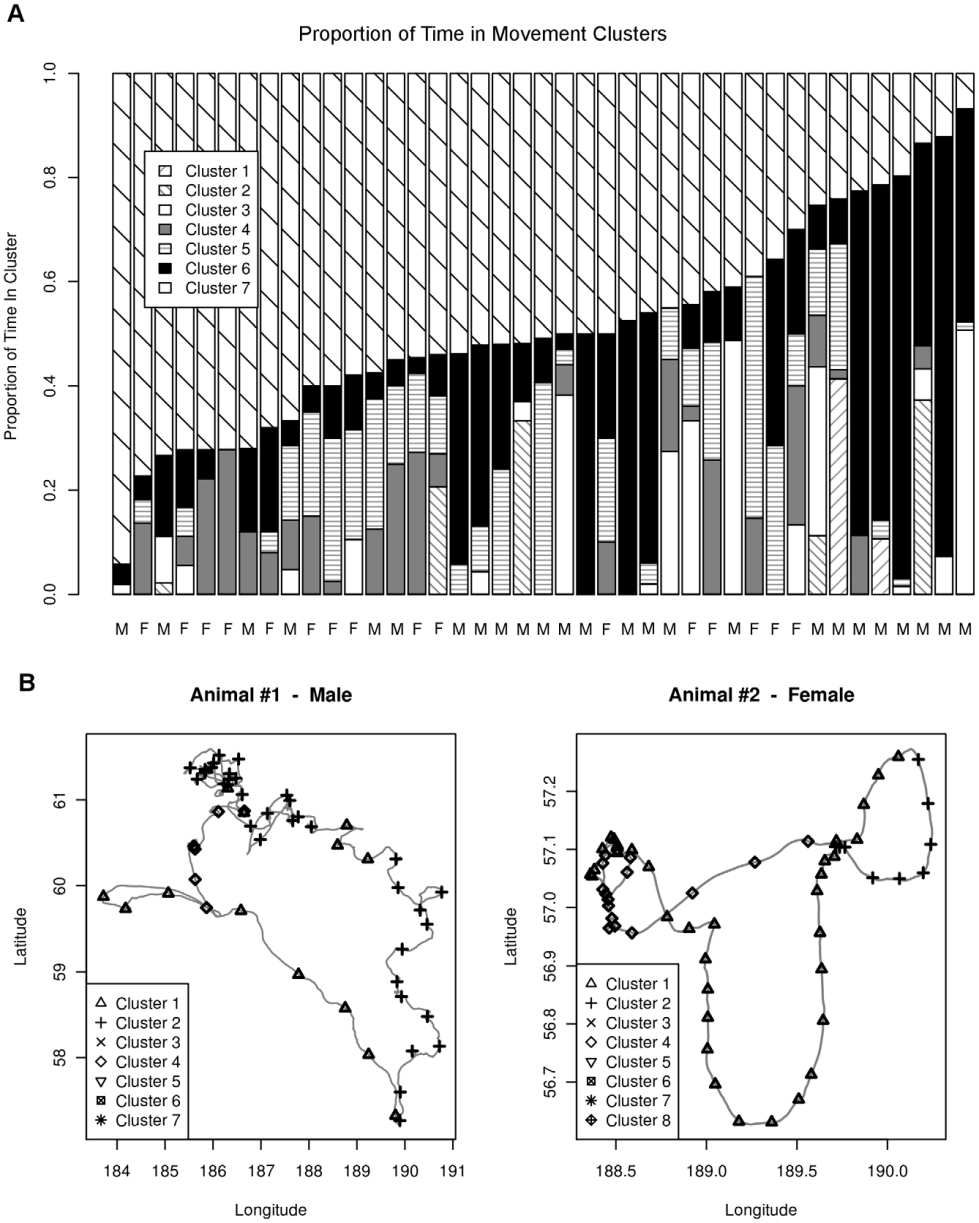
Cluster Analysis Results. In (a), the barplot shows the proportion of trip time each seal spends in each of the eight movement clusters. In (b), two example paths are shown. Animal 1, a male NFS, spends a significant proportion of its trip in clusters 1 and 2, while animal 2, a female NFS, spends a significant amount of time in clusters 1, 2 and 4.

For population-level inference, the aggregated multivariate responses to drivers of movement 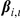 were clustered in models with 1–8 mixture components, with the 7-component model having the best BIC. Violin plots of the marginal distributions of 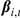 for these clusters, labeled 1–7 in order of decreasing prevalence, are shown in Figure 6. The proportion of time each animal spent in each movement cluster is shown in Figure 7a, and Figure 7b shows the movement paths of the example animals 1 and 2 with time spent in each movement cluster. Similar plots for the 39 other animals are shown in the supporting information (Text S3).

**Figure 8:**
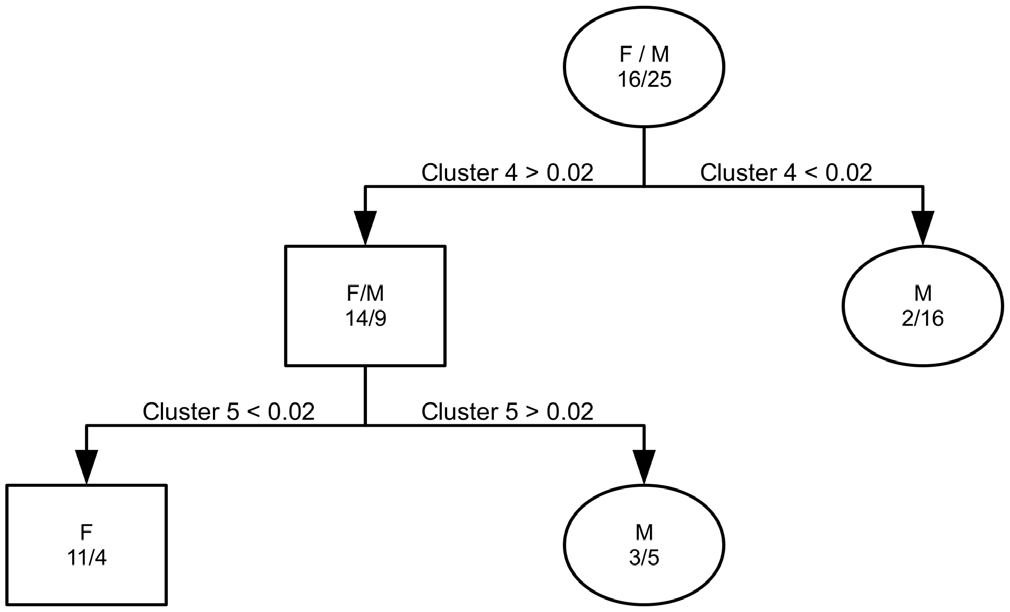
Gender differences in proportion of time spent in movement clusters. A classification tree with sex as the response variable and proportion of time spent in each of the seven movement clusters as the predictor variables reveals that female NFS spend proportionately more time in cluster 4, a movement state of higher absolute speed and response to Chlorophyll *a*, sea surface temperature, and net primary production than do male NFS.

The classification tree with sex as the response variable and proportion of time spent by each animal in each of the seven movement clusters is shown in Figure 8. This classification tree correctly identifies northern fur seal sex 78% of the time, and shows that female NFS spend proportionately more time in cluster 4 and less time in cluster 5 than do males.

The best classification tree for male NFS with year tagged as the response variable had no splits, indicating that male NFS tagged in 1999 do not spend significantly different proportions of time in any movement cluster than do male NFS tagged in 2000.

## Discussion

### Northern Fur Seal Discussion

For both animal 1 and animal 2, the model with the best DIC includes only the distance to rookery covariate, indicating that these animals were not strongly influenced by the environmental factors represented by the other covariates (Table 1).

The results concerning 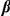 for animal 1, shown in Figure 4a and Figure 5a, confirm that the distance to rookery covariate does have a significant effect which changes over time. At the beginning of the seal's trip, the positive effect of this covariate indicates a desire to move away from the rookery, possibly towards memorized foraging locations. During the middle of the trip, the lack of any significant covariates indicates that movement is not being driven by the covariates in our study, but by some other drivers. Potential drivers of movement not examined in this study include subsurface temperatures and behavioral drivers (e.g., prey and predator locations). At around hour 475, the effect of the distance to rookery covariate becomes negative, driving the male to return to the rookery.

The results for animal 2 (Figure 4b and Figure 5b) show that this female NFS is first pulled away from the rookery (hours 3–10), then is not driven by any of the covariates in this study for a period of time (hours 19–38). After approximately 38 hours, the animal begins to return to the rookery, though the return is quite different than that of animal 1. The second half of the female's trip (after approximately 50 hours) is dominated by little response to any of the covariates in the study. The female appears to be foraging on the return trip, and forages close to the rookery between hours 66 and 83. In contrast, the path of animal 1 shows a relatively long period in which the animal is drawn out away from the rookery, followed by a fairly abrupt change in response to the distance to rookery driver (around hour 475), and a short time in which the animal returns to the rookery with little distraction. This juvenile male NFS appears to have a fairly distinct turnaround time, in which it decides to cease foraging and return quickly to the rookery.

A comparison of the scale of 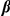 in Figure 4 shows that the female's absolute response to all covariate gradients is larger in magnitude than the male's response. This indicates that the female traveled at a higher swim velocity than the male in these example trips. The female's trip is also much shorter than that of animal 1. This is typical of the NFS studied here (see Text S3).

One possible explanation for these differences may be tied to the different roles male and female northern fur seals play in rearing pups. The female northern fur seals are the primary providers for unweaned pups, and may be more constrained in their foraging, as they must return to feed their pups. Male northern fur seals, like animal 1, may be able to forage in a less-constrained manner, and thus are less driven to return within a certain time interval, leading to a slower swim speed and longer duration of foraging trips. Additional individual-level results are in the supplemental material (Text S3).

In our population-level study, the movement of all NFS studied was clustered into seven mixture components, representing seven distinct movement profiles for this population (Figure 6). Cluster 1 is the most prevalent cluster of NFS response to the four drivers of movement, and contains values of 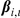 that are fairly representative for all covariates. Cluster 2 differs from cluster 1 primarily in its responses to CHA, SST, and NPP, which are all more closely distributed around zero than in cluster 1, indicating a state of movement where influence of environmental factors is minimal. Cluster 4 is characterized by extreme response (both positive and negative) to all covariates, indicating a state of fast absolute speed, as extreme responses to any covariate correspond to high velocity in the animal's movement. Cluster 5 is characterized by small (near zero) response to all covariates, indicating a state of slow absolute speed. extreme response to NPP.

The clustering algorithm used for this population-level study of NFS movement patterns is based solely on the aggregated responses to drivers of movement 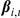, and does not take into account the animal or the time in the trip. However, the 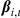 arise from our individual-based change point model of animal movement, which assumes a partition of the movement path into contiguous regions in which the response to the environment is similar. Thus it is not surprising to see that the animal movement paths in Figure 7b and the supporting information (Text S3) show both animals tend to remain in one movement cluster for a time and then switch to another cluster.

Cluster 5, which is a movement state of low absolute response to the environment, is a predominantly male response to the environment (Figure 8). Cluster 4, which is a predominantly female movement state (Figure 8), is characterized by larger absolute response to covariates. This cluster is a state of faster absolute movement and stronger response to the environment than is found in the predominantly male cluster 5. This may indicate that female NFS are more intense foragers than males, as the length of their foraging trips are constrained by the amount of time their pups can survive between feedings. Males, on the other hand, have no such constraints, and may be able to forage in a more relaxed fashion.

The results from the male-only comparison of movement between seals tagged in 1999 and 2000 show no significant differences in response to the drivers of movement used in this study. The velocity-based approach we have used for modeling animal movement is based on gradients of covariates, and responses to these gradients were similar for males tagged in both years. Sea surface temperatures were colder in 1999 than in 2000, but male NFS movement patterns were not greatly affected by this change in temperature.

### Modeling Approach

We have presented an approach for modeling animal movement based on random walks (CTCRW) that allows for inference on changing movement profiles exhibited by the animal throughout the time for which we have location information. This approach for linking animal telemetry data to environmental and biotic drivers of movement is flexible, computationally efficient, and yields easily interpretable results. A general overview of our approach to individual and population-level inference could be summarized as follows.

1. Obtain time-referenced location data 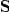 for multiple animals in a population.
2. For each animal, utilize the CTCRW model [15], or some comparable method, to obtain realizations from the posterior predictive path distribution 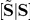, with locations at regular time intervals.
3. Obtain spatially-referenced covariates that are hypothesized drivers of movement, and calculate their gradients.
4. Make inference about drivers of movement for each individual animal using the velocity-based movement approach (6)–(10). This approach accounts for the uncertainty in the movement path distribution and allows for temporal heterogeneity in the drivers of movement.
5. Aggregate values of 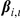 for all animals and at all times and cluster these aggregated effects in parameter space. Insight into the interpretation of each cluster's movement profile can be obtained by comparison of the marginal distributions of the effects in each cluster.
6. Make inference about differences in how population subsets respond to drivers of movement by using the proportion of time spent by individual animals in each movement cluster as predictor variables. This could be done using parametric (e.g., logistic regression) or nonparametric (e.g., classification trees) statistical methods.

Care should be taken when making conclusions based on the posterior number of partitions 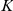, as this parameter can be sensitive to model departures [22]. The change point model we have used assumes that an animal's response to a covariate is constant on each of 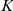 partitions of the observation period. This model can approximate much more complicated time-varying behavior, as shown in our simulation study (Text S2), but if the true response to a covariate is not piece-wise constant, then the location data will likely drive 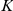 to be a large number to best approximate the changing behavior over time. For example, the distance to rookery covariate seems to vary smoothly over time for animal 1 in our application, while our model assumes a finite number of break points in which the animal changes behavior. If the covariate does vary smoothly, the model would tend to favor a large number of partitions in an effort to best capture the animal's true response to the driver of movement. Stephens [22] also notes that inference on 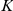 can be influenced by choice of prior distribution and birth distribution. We have found this to be true, though inference on 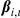, the focus of our study, is quite resilient to these variations.

A similar, but alternative approach to our change-point model would be a temporally varying coefficient model [45]–[47], which could be used to obtain similar inference about environmental drivers of movement within the velocity-based framework we have described here.

Utilizing the velocity-based model with an unknown number of change points requires tuning the birth distribution by adjusting 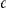 in (14). An alternative that would require less human input would be to fit multiple fixed change point models with different numbers of change points (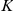) and rank models using standard model selection criteria [36]. For example, Steele and Raftery [48] have found that BIC can outperform other Bayesian selection criteria (including DIC) in choosing the number of mixture components (K) in a model. In the data augmentation case, BIC can be formulated as
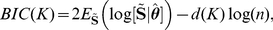
(13)where 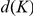 is the total number of free parameters in the model with 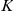 movement states and 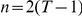 is the number of observations. In the fixed-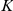 case, this formulation of BIC can be used to compare models with differing numbers of change points.

Our individual-level model allows for heterogeneous behavior over time, as implemented with a change point model. In its current form, we estimate 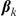 for each partition (6) independent of all other partitions. This allows animal movement behavior in each partition, as characterized by the animal's response to the potential drivers of movement, to be different from behavior in all other partitions. It may be more realistic to assume that there are a fixed (or random and unknown) number of movement states that an animal can exhibit, and the observed path can be partitioned in such a way that movement behavior in each temporal partition follows one of the fixed (or random) movement states. For example, we might expect animals to act similarly at specific times of the day each day (e.g., dormant behavior during the day for nocturnal animals), resulting in partitions each day with similar movement behavior. Our simulation study (Text S2) shows that our individual-level approach can make inference on recurring behavior of this form, but explicitly incorporating this into our model, perhaps through a hierarchical prior on the 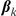 that is shared by the population, could make our approach more robust. The main difficulties here are computational, as tuning the nested birth-death processes for individual and population-level heterogeneity may be infeasible.

In our approach to modeling individual and population-level movement, we have assumed that each animal moves independently of all other animals. While this is not likely to be true in all situations, jointly modeling the relationship between movement patterns of conspecifics is challenging. One major difficulty is that only a sample of a population is typically monitored, leading to little overlap between the animal movement paths in space and time. One way the relationship between conspecific movement patterns could be incorporated into the current framework (5)–(6) would be to jointly model all movement steps 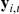 from all animals and specify a covariance structure based on the spatial and temporal separation between the movement steps of different animals. For example, the correlation between 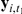 and 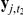, two movement steps from different animals at possibly different times, could be modeled with an exponentially-decaying structure: 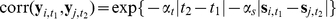, where 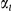 and 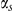 are range parameters for correlation between movement in time and space, respectively. In this way, animals that are close together in time and space could exhibit correlated movement and response to the environment.

In the estimation of resource selection functions, a distribution of available habitat is typically specified [15], [49]. The definition of available habitat can be set by the study area [9], [50] or by an underlying movement model [12], [21], [51]. Inference on use versus availability of resources can vary as the scale of what is defined as available changes [9], [50], [52]. Our approach to modeling animal movement and resource selection could be seen as defining availability of resources at two scales. Our velocity-based model with covariate gradients assumes that animals are motivated only by what is in their immediate neighborhood, thus our availability distribution amounts to the adjacent habitat at the scale of the environmental covariates (e.g., the adjacent pixels of land cover types). Jointly with this fine-scale definition of availability, we employ a stochastic CTCRW model of animal movement to stochastically integrate over our uncertainty in the animal's path between location fixes. Thus, in our approach, availability of resources is defined by local environmental gradients of the posterior distribution of movement paths in the CTCRW model.

In this study, we have modeled environmental drivers of movement as local covariate gradients fixed in time, but the integration of environmental factors in various formats could facilitate inference on effects not easily addressed in the current framework. Three possible extensions to the existing framework are the inclusion of temporally-varying environmental covariates, known barriers to animal movement, and larger scale gradients. We consider each of these in turn.

The approach we have presented allows the effect of environmental factors (e.g., 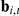) to vary over time, but the environmental factors themselves (e.g., SST) are held constant in time. This may not always be the case, especially in a marine environment. Currents are constantly shifting, and the ocean is always in flux. The use of potential environmental covariates in the existing framework for making inference about drivers of animal movement will allow for more realistic inference.

Our approach does not currently take into account potential barriers to animal movement, such as land masses for marine animals or cliffs for land animals. Such barriers could be included through rejection sampling of 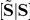 in the data augmentation step (3), accepting a continuous path realization 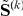 only if it does not cross the known movement barrier.

Our approach to expressing environmental drivers as gradients currently considers only local gradients, though animal movement may be motivated by memory of desirable destinations at a greater distance. A possible extension to our current approach could be to use larger scale (coarser resolution) gradients simultaneously with local environmental gradients. This could provide insight into when an animal's movement is being motivated by local environmental factors, as opposed to distant attractions. This could allow for models that predict animal movement through less desirable landscape features to destinations that are highly desirable. Our velocity-based approach for animal movement modeling is flexible and extensible, and its computational efficiency enables modeling significant complexity in an animal's response to the environment.

## Supporting Information

Text S1 **BDMCMC Algorithm.** This supplement consists of a description of the BDMCMC algorithm used in our study. We describe the particulars of the birth distribution used, and outline the steps for implementing the BDMCMC process.
(PDF)

Text S2 **Simulation Study.** This supplement contains a simulation study illustrating our approach to individual and population-level inference on animal movement.
(PDF)

Text S3 **Individual-Level Results.** This supplement contains plots of individual-level results for all animals.
(PDF)

## References

[1] Dalziel BD, Morales JM, Fryxell JM (2008). Fitting probability distributions to animal movement trajectories: using artificial neural networks to link distance, resources, and memory.. The American Naturalist.

[2] Gurarie E, Andrews RD, Laidre KL (2009). A novel method for identifying behavioural changes in animal movement data.. Ecology Letters.

[3] Cagnacci F, Boitani L, Powell Ra, Boyce MS (2010). Animal ecology meets GPS-based radioteleme6 79 try: a perfect storm of opportunities and challenges.. Philosophical Transactions of the Royal Society of London Series B, Biological Sciences.

[4] Tomkiewicz SM, Fuller MR, Kie JG, Bates KK (2010). Global positioning system and associated technologies in animal behaviour and ecological research.. Philosophical Transactions of the Royal Society of London Series B, Biological Sciences.

[5] Kuhn CE, Johnson DS, Ream RR, Gelatt TS (2009). Advances in the tracking of marine species: using GPS locations to evaluate satellite track data and a continuous-time movement model.. Marine Ecology Progress Series.

[6] Morales JM, Haydon DT, Frair J, Holsinger KE, Fryxell JM (2004). Extracting more out of relo cation data: building movement models as mixtures of random walks.. Ecology.

[7] Nathan R, Getz W, Revilla E, Holyoak M, Kadmon R (2008). A movement ecology paradigm for unifying organismal movement research.. Proceedings of the National Academy of Sciences.

[8] Getz W, Saltz D (2008). A framework for generating and analyzing movement paths on ecological landscapes.. Proceedings of the National Academy of Sciences.

[9] Forester JD, Im HK, Rathouz PJ (2009). Accounting for animal movement in estimation of resource selection functions: sampling and data analysis.. Ecology.

[10] Merrill E, Sand H, Zimmermann B, McPhee H, Webb N (2010). Building a mechanistic understanding of predation with GPS-based movement data.. Philosophical Transactions of the Royal Society B: Biological Sciences.

[11] Polansky L, Wittemyer G, Cross P, Tambling C, Getz W (2010). From moonlight to movement and synchronized randomness: Fourier and wavelet analyses of animal location time series data.. Ecology.

[12] Aarts G, MacKenzie M, McConnell B, Fedak M, Matthiopoulos J (2008). Estimating space-use and habitat preference from wildlife telemetry data.. Ecography.

[13] Morales JM, Moorcroft PR, Matthiopoulos J, Frair JL, Kie JG (2010). Building the bridge between animal movement and population dynamics.. Philosophical Transactions of the Royal Society B: Biological Sciences.

[14] Jonsen I, Flemming J, Myers R (2005). Robust state-space modeling of animal movement data.. Ecology.

[15] Johnson DS, Thomas DL, Ver Hoef JM, Christ A (2008). A general framework for the analysis of animal resource selection from telemetry data.. Biometrics.

[16] Tremblay Y, Robinson PW, Costa DP (2009). A parsimonious approach to modeling animal move ment data.. PloS ONE.

[17] Sumner MD, Wotherspoon SJ, Hindell Ma (2009). Bayesian estimation of animal movement from archival and satellite tags.. PloS ONE.

[18] Johnson D, London J, Kuhn C (2011). Bayesian Inference for Animal Space Use and Other Move ment Metrics.. Journal of Agricultural, Biological, and Environmental Statistics.

[19] McClintock BT, King R, Thomas L, Matthiopoulos J, McConnell B (2011). A general mod eling framework for animal movement and migration using multi-state random walks.. Ecological Monographs.

[20] Gentry R (1998). Behavior and ecology of the northern fur seal.

[21] Hooten MB, Wikle CK (2010). Statistical agent-based models for discrete spatio-temporal systems.. Journal of the American Statistical Association.

[22] Stephens M (2000). Bayesian analysis of mixture models with an unknown number of components - an alternative to reversible jump methods.. The Annals of Statistics.

[23] Turchin P (1998). Quantitative Analysis of Movement.

[24] Rubin DB (1987). Multiple Imputation for Nonresponse in Surveys.

[25] Rubin DB (1996). Multiple imputation after 18+ years.. Journal of the American Statistical Asso ciation.

[26] Brockwell P, Davis R (2002). Introduction to Time Series and Forecasting.

[27] Hooten MB, Johnson DS, Hanks EM, Lowry JH (2010). Agent-based inference for animal movement and selection.. Journal of Agricultural, Biological, and Environmental Statistics.

[28] Cressie N, Calder CA, Clark JS, Ver Hoef JM, Wikle CK (2009). Accounting for uncertainty in ecological analysis: the strengths and limitations of hierarchical statistical modeling.. Ecological Applications.

[29] Gelman A, Carlin BP, Stern H, Rubin DB (2004). Bayesian Data Analysis.

[30] Green PJ (1995). Reversible jump markov chain monte carlo computation and bayesian model determination.. Biometrika.

[31] Cappe O, Robert CP, Ryden T (2003). Reversible jump, birth-and-death and more general contin uous time Markov chain Monte Carlo samplers.. Journal of the Royal Statistical Society: Series B (Statistical Methodology).

[32] Akaike H (1974). A new look at the statistical model identification.. IEEE Transactions on Automatic Control.

[33] Schwarz G (1978). Estimating the dimension of a model.. The Annals of Statistics.

[34] Spiegelhalter DJ, Best NG, Carlin BP, van der Linde A (2002). Bayesian measures of model com plexity and fit.. Journal of the Royal Statistical Society: Series B (Statistical Methodology).

[35] Celeux G, Forbes F, Robert CP, Titterington DM (2006). Deviance information criteria for missing data models.. Bayesian Analysis.

[36] Burnham K, Anderson D (2002). Model selection and multimodel inference: a pactical information theoretic approach.

[37] Izenman AJ (2008). Modern Multivariate Statistical Techniques: Regression, Classification, and Manifold Learning.

[38] Kenyon KW, Wilke F (1953). Migration of the northern fur seal, Callorhinus ursinus.. Bulletin of the Ecological Society of America.

[39] Loughlin TR, Antonelis GA, Baker JD, York AE, Fowler CW (1994).

[40] Duchon J (1977).

[41] Furrer R, Nychka D, Sain S (2010). fields: Tools for spatial data (accessed 2011).. http://cran.r-project.org/package=fields.

[42] R Development Core Team (2009). R: A Language and Environment for Statistical Computing..

[43] Fraley C, Raftery A (2006). MCLUST version 3 for R: Normal mixture modeling and model-based clustering (accessed 2011).. http://cran.r-project.org/web/packages/mclust/index.html.

[44] Breiman L, Freidman J, Stone C, Olshen R (1984). Classification and Regression Trees.

[45] Hastie T, Tibshirani R (1993). Varying-Coefficient Models.. Journal of the Royal Statistical Society: Series B (Statistical Methodology).

[46] Wheeler DC, Calder CA (2007). An assessment of coefficient accuracy in linear regression models with spatially varying coefficients.. Journal of Geographical Systems.

[47] Ver Hoef JM, London JM, Boveng PL (2009). Fast computing of some generalized linear mixed pseudo-models with temporal autocorrelation.. Computational Statistics.

[48] Steele RJ, Wang N, Raftery AE (2010). Inference from multiple imputation for missing data using mixtures of normals.. Statistical Methodology.

[49] Manly B (2002). Resource selection by animals: statistical design and analysis for field studies.

[50] Beyer H, Haydon D, Morales J, Frair J, Hebblewhite M (2010). The interpretation of habitat preference metrics under use–availability designs.. Philosophical Transactions of the Royal Society B: Biological Sciences.

[51] Christ A, Hoef J, Zimmerman D (2008). An animal movement model incorporating home range and habitat selection.. Environmental and Ecological Statistics.

[52] Senft R, Coughenour M, Bailey D, Rittenhouse L, Sala O (1987). Large herbivore foraging and ecological hierarchies.. BioScience.

