# Supplementary material for: Velocity-Based Movement Modeling for Individual and Population Level Inference
Source: PLoS One. 2011 Aug 11;6(8):e22795. doi: 10.1371/journal.pone.0022795 (PMC3154913; doi:10.1371/journal.pone.0022795)
Supplement: Text S1 — BDMCMC Algorithm. This supplement consists of a description of the BDMCMC algorithm used in our study. We describe the particulars of the birth distribution used, and outline the steps for implementing the BDMCMC process. (PDF) [file pone.0022795.s001.pdf]

# Supporting Information S1 - Details of the Birth-Death Markov Chain Monte Carlo Process

Birth-death Markov chain Monte Carlo (BDMCMC) methods are a type of continuous time Markov chain Monte Carlo (CTMCMC) methods, which are used to construct ergodic chains for models with a varying parameter space [1,2]. In this appendix we present a brief overview of the BDMCMC algorithm used in this paper, and refer interested readers to Stephens [1] and Cappe et al. [2] for a full treatment.

In BDMCMC, each mixture component is viewed as a point in a multidimensional parameter space. In our case, each mixture component is a movement partition, and is defined by a $p+1$ dimensional parameter vector: $(\boldsymbol{\beta}'_k, \tau_k)$, where $p$ is the number of regression coefficients in the model (and thus $\boldsymbol{\beta}_k$ is a $p$-vector), and $\tau_k$ is the change point at the beginning of the movement partition.

The BDMCMC process allows new points to be "born" into the parameter space, corresponding to the addition of a new partition of the movement path, and existing points to "die", corresponding to the joining of two adjacent partitions into one partition of the movement path. This is accomplished by supplying a time $t_{bd}$ that the birth-death process will be run for each MCMC iteration, a birth rate $\pi_{\text{birth}}$ which determines how often (in the birth-death process) a new partition is born, and a birth distribution $[\boldsymbol{\beta}', \tau]_{\text{birth}}$ from which the parameters specifying the new partition are generated. The birth-death process, which is step 3 of the MCMC algorithm in our paper, proceeds as follows.

1. Set $t_{\text{left}} = t_{bd}$.

2. Calculate the death rate $\delta_k$ for each of the $K$ existing partitions

$$\delta_k = \frac{[\mathbf{y}|\{\boldsymbol{\beta}'_i, \tau_i\} \setminus (\boldsymbol{\beta}'_k, \tau_k)]}{[\mathbf{y}|\{\boldsymbol{\beta}'_i, \tau_i\}]} \cdot \frac{\pi_{\text{birth}}\ [\boldsymbol{\beta}'_k, \tau_k]_{\text{birth}}}{\lambda\ [\boldsymbol{\beta}'_k, \tau_k]_{\text{prior}}},$$

   where $[\mathbf{y}|\{\boldsymbol{\beta}'_i, \tau_i\} \setminus (\boldsymbol{\beta}'_k, \tau_k)]$ is the likelihood of the data $\mathbf{y}$ if the $k$-th partition is removed from the movement model, $[\mathbf{y}|\{\boldsymbol{\beta}'_i, \tau_i\}]$ is the likelihood of the data $\mathbf{y}$ in the model containing the $k$-th partition, $[\boldsymbol{\beta}'_k, \tau_k]_{\text{birth}}$ is the likelihood of the $k$-th partition in the birth distribution, $\lambda$ is the Poisson rate parameter of the prior distribution of the number of model states $K$, and $[\boldsymbol{\beta}'_k, \tau_k]_{\text{prior}}$ is the model prior likelihood of the $k$-th partition.

3. Calculate the total death rate $\pi_{\text{death}} = \sum_k \delta_k$

4. Simulate the time to the next birth or death. As this is the inter-event time of multiple Poisson processes, the time to next birth or death is exponentially distributed:

$$t_{\text{current}} \sim \text{Exp}\left(\frac{1}{\pi_{\text{birth}} + \pi_{\text{death}}}\right).$$

5. If $t_{\text{current}} < t_{\text{left}}$, continue. Otherwise, exit the birth-death process.

6. Determine whether a birth or a death occurs:

$$P(\text{birth}) = \frac{\pi_{\text{birth}}}{\pi_{\text{birth}} + \pi_{\text{death}}}$$

7. If a birth occurs, generate the new parameters of the partition from the birth distribution $[\boldsymbol{\beta}', \tau]_{\text{birth}}$. If a death occurs, remove one of the existing partitions. The probability that partition $k$ is removed is $\delta_k / \pi_{\text{death}}$.

8. Repeat steps 2 - 7 until $t_{\text{current}} \geq t_{\text{left}}$.

This birth-death process is repeated for each MCMC iteration.

One of the main benefits of using a BDMCMC process over a RJMCMC process is the ease with which multiple birth distributions can be implemented. Changing the birth distribution only affects the calculations in step 2, which makes it easy to experiment with multiple birth distributions. The success of this process is dependent on finding a combination of birth distribution, prior distributions for model parameters, and $\pi_{\text{birth}}$ (the birth-death time) that results in good mixing in the MCMC chain.

# References

1. Stephens M (2000) Bayesian analysis of mixture models with an unknown number of components - an alternative to reversible jump methods. The Annals of Statistics 28: 40–74.

2. Cappe O, Robert CP, Ryden T (2003) Reversible jump, birth-and-death and more general continuous time Markov chain Monte Carlo samplers. Journal of the Royal Statistical Society: Series B (Statistical Methodology) 65: 679–700.

# A R-Code

We provide here R-Code to implement the velocity-based movement model with an unknown (random) number of change points.

```
#
#
#  Velocity-based movement model with an
#  unknown number of change points (BDMCMC)
#
#  Author: Ephraim Hanks
#  Contact: hanks@stat.colostate.edu
#
#  Last Updated: 20110603
#
#  Reference:
#   Hanks EM, MB Hooten, DS Johnson, and JT Sterling. Velocity-based
#   movement modeling for individual and population level inference.  PLoS One.
#
#
#  Required Packages: cellmove.jabes
#  (available from http://warnercnr.colostate.edu/~hooten/other/)
#
#
#Inputs:
#
#  sim.obj - A fit from the CRAWL continuous time correlated random walk model
#            on the telemetry data in question.
#  Cov.df  - A list containing the following elements:
#            1. X - a data frame with the x and y locations of covariate
#                   grid cells, and the actual covariate values at those
#                   locations
#            2. X.grad.x - a data frame containing the x component of the
#                   gradient of each covariate, by columns.
#            3. X.grad.y - a data frame containing the y component of the
#                   gradient of each covariate, by columns.
```

```
#            4. xy - a 2-column matrix containing the x and y locations
#                   of covariate grid cells
#  lambda  - prior parameter for K
#  max.partitions - the maximum number of partitions allowed by the model
#  tau.min - smallest time value where change points are allowed
#            (needed to keep a partition from collapsing to only 1 or 2 time points)
#  tau.max - largest time value where change points are allowed
#            (needed to keep a partition from collapsing to only 1 or 2 time points)
#  tau.start - vector of start times for the change points tau
#  tau.tune - tuning parameter (bandwidth of proposal dist'n for each tau)
#  tau.buffer - smallest time allowed between change points
#            (needed to keep a partition from collapsing to only 1 or 2 time points)
#  betastrt - start values for beta (list with one vector for each starting partition)
#  betamean - prior mean for beta (one vector - shared by all partitions)
#  betavar - prior variance for beta (one number - shared by all betas)
#  s2.start - start value for sigma^2
#  s2.mean - prior mean on the IG dist'n for sigma^2
#  s2sd - prior standard deviation on the IG dist'n for sigma^2
#  timestep.bd - "length of time" to run the Birth-Death process (default=1)
#  birth.rate - Poisson rate of birth in BDMCMC process
#  birth.dist - Birth distribution - a list with the following elements
#             1. beta.means - a p by T matrix with mean values for the
#                betas at each time point
#             2. beta.sd - a p by T matrix with standard deviations
#                for the betas at each time point
#  bd.var.inf - Tuning parameter in Birth-Death process
#  n.mcmc - Number of MCMC iterations to run
#  show.tau - Logical.  If TRUE, then the change points (tau) are displayed at
#             each iteration.  Useful for guaging how well the BDMCMC process
#             is mixing.


move.reg.bd.pois <-function(sim.obj,Cov.df,lambda,max.partitions,tau.min,
tau.max,tau.start,tau.tune,tau.buffer,betastrt,betamean,betavar,s2.start,
s2mean,s2sd,timestep.bd=1,birth.rate=1,birth.dist,bd.var.inf=1,n.mcmc,
show.tau=FALSE){


###
### subroutines
###

invgammastrt <- function(igmn,igvar){
  q <- 2+(igmn^2)/igvar
  r <- 1/(igmn*(q-1))
  list(r=r,q=q)
}


get.Q <- function(X,X.grad.x,X.grad.y,loc.idx){
  n.X=dim(X.grad.x)[2]
  path.length=length(loc.idx)
  Q=matrix(NA,nrow=2*(path.length-1),ncol=n.X)
  for(i in 1:n.X){
```

```
    Q[,i] <- c(X.grad.x[loc.idx[-path.length],i],X.grad.y[loc.idx[-path.length],i])
  }
  Q
}


get.path.idx <- function(xy,path.list){
  min.x = min(diff(sort(xy[, 1]))[diff(sort(xy[, 1])) > 0])
  min.y = min(diff(sort(xy[, 2]))[diff(sort(xy[, 2])) > 0])
  x.uniq = sort(unique(xy[, 1]))
  y.uniq = sort(unique(xy[, 2]))
  ny = length(y.uniq)
  nx = length(x.uniq)
  x.breaks = c(x.uniq - min.x/2, max(x.uniq) + min.x/2)
  y.breaks = c(y.uniq - min.y/2, max(y.uniq) + min.y/2)
  tmp.x.g = findInterval(path.list[, 1], x.breaks)
  tmp.y.g = findInterval(path.list[, 2], y.breaks)
  tmp.x.y.g = (tmp.y.g-1)*nx + tmp.x.g
  tmp.rle = rle(tmp.x.y.g)
  tmp.d = tmp.rle$lengths
  tmp.locs = tmp.rle$values
  tmp.locs.full=rep(tmp.locs,tmp.d)
  path.idx.list <- tmp.locs.full[-length(tmp.locs.full)]
  path.idx.list
}



mv <- function(z,Q,tau,beta){
  mvec=rep(NA,length(z))
  tau.aug=c(tau,length(z)/2+1)
  for(r in 1:length(tau)){
      idx.current=c(tau.aug[r]:(tau.aug[r+1]-1),(length(z)/2+tau.aug[r]):(length(z)/2+tau.aug[r+1]-1))
      if(ncol(Q)>1){
        mvec[idx.current] <- Q[idx.current,]%*%beta[[r]]
      }
      else{
        mvec[idx.current] <- Q[idx.current,]*beta[[r]]
      }
  }
  mvec
}


##
## Preliminaries
##
  birth.beta.means=birth.dist$beta.means
  birth.beta.sd=bd.var.inf*birth.dist$beta.sd
  birth.tau.dens=birth.dist$tau.prob



X=Cov.df$X
```

```
X.grad.x=Cov.df$X.grad.x
X.grad.y=Cov.df$X.grad.y
xy=Cov.df$xy


datetime=sim.obj$datetime
datetime=datetime[1:(length(datetime)-2)]


###
### hyperpriors
###

#browser()

n=dim(X.grad.x)[1]
p=dim(X.grad.x)[2]
num.part=length(tau.start)+1

beta0=matrix(betamean,p,1)
Sig0=betavar*diag(p)
r=invgammastrt(s2mean,(s2sd^2))$r
q=invgammastrt(s2mean,(s2sd^2))$q

params.save=list()
numstates.save=rep(NA,n.mcmc)



###
### start values
###

beta=list()
for(i in 1:length(betastrt)){
  beta[[i]] <- betastrt[[i]]
}
beta[[length(betastrt)+1]] <- length(betastrt)
tau=tau.start
s2=s2.start
acc.count=0
K=length(tau)



###
### Start Crawl path
###

keep.idx=0
  while(keep.idx==0){
    samp.new <- crwPostIS(sim.obj, fullPost=FALSE)
    path.list=cbind(samp.new$alpha.sim.x[,'mu'], samp.new$alpha.sim.y[,'mu'])
    path.loc.idx=get.path.idx(xy,path.list)
```

```
    if(min(path.loc.idx)>0 & max(path.loc.idx)<10000){
      keep.idx=1
    }
  }
  y.seal=path.list[-1,]-path.list[-dim(path.list)[1],]
  Q=get.Q(X,X.grad.x,X.grad.y,path.loc.idx)
  z=c(y.seal[-(dim(y.seal)[1]),1],y.seal[-(dim(y.seal)[1]),2])
  meanvec=mv(z,Q,tau,beta)
  loglik.current=sum(dnorm(z,meanvec,sqrt(s2),log=TRUE))

#samp <- crwPostIS(sim.obj, fullPost=FALSE)
#path.list=cbind(samp$alpha.sim.x[,'mu'], samp$alpha.sim.y[,'mu'])
#path.loc.idx=get.path.idx(xy,path.list)
#y.seal=path.list[-1,]-path.list[-dim(path.list)[1],]
#Q=get.Q(X,X.grad.x,X.grad.y,path.loc.idx)
#z=c(y.seal[-(dim(y.seal)[1]),1],y.seal[-(dim(y.seal)[1]),2])
#T=length(z)/2
#
#browser()
#meanvec=mv(z,Q,tau,beta)
#loglik.current=sum(dnorm(z,meanvec,sqrt(s2),log=TRUE))
#cat("start ",loglik.current,"\n")

###
### gibbs loop
###

#browser()

for(m in 1:n.mcmc){


  ###
  ### Birth-Death Process
  ###

  #browser()

  time.bd.left=timestep.bd
  start.comp.time=proc.time()[3]
  tot.time=0
  while(time.bd.left>0 & tot.time<10){
    ## Calculate death rate for each existing point
    delta=rep(0,length(tau))
    if(length(tau)>1){
      ## death rate for first partition
      beta.d1=beta
      beta.d1[[1]] <- NULL
      meanvec.death=mv(z,Q,tau[-2],beta.d1)
      loglik.death=sum(dnorm(z,meanvec.death,sqrt(s2),log=TRUE))
      loglik.birth=sum(dnorm(beta[[1]],birth.beta.means[1,],birth.beta.sd[1,],log=TRUE))
      loglik.prior=sum(dnorm(beta[[1]],betamean,sqrt(betavar),log=TRUE))
      delta[1] <- birth.rate/lambda*exp(loglik.death-loglik.current+loglik.birth-loglik.prior)
      ## death rate for all subsequent partitions
```

```
  for(k in 2:length(tau)){
    beta.temp=beta
    beta.temp[[k]] <- NULL
    meanvec.death=mv(z,Q,tau[-k],beta.temp)
    loglik.death=sum(dnorm(z,meanvec.death,sqrt(s2),log=TRUE))
    loglik.birth=sum(dnorm(beta[[k]],birth.beta.means[tau[k],],birth.beta.sd[tau[k],],log=TRUE))
    loglik.prior=sum(dnorm(beta[[k]],betamean,sqrt(betavar),log=TRUE))
    delta[k]=birth.rate/lambda*exp(loglik.death-loglik.current+loglik.birth-loglik.prior)

  }
}

death.rate=sum(delta)
## see if a birth or death happens in the remaining time
if(death.rate==Inf){
  ## Death of offending location
  dead.comp=which.max(delta)
  if(dead.comp==1){
    tau=tau[-2]
    beta[[1]] <- NULL
  }
  else{
    tau=tau[-dead.comp]
    beta[[dead.comp]] <- NULL
  }
  meanvec=mv(z,Q,tau,beta)
  loglik.current=sum(dnorm(z,meanvec,sqrt(s2),log=TRUE))
}
else{
#browser()
time.star=rexp(1,1/(birth.rate+death.rate))
time.bd.left=time.bd.left-time.star
if(time.bd.left>0){
  if(runif(1)>birth.rate/(birth.rate+death.rate) | length(tau)==max.partitions){
    ## Death
    if(death.rate>0){
      dead.comp=which(rmultinom(1,1,delta/death.rate)==1)
      if(dead.comp==1){
        tau=tau[-2]
        beta[[1]] <- NULL
      }
      else{
        tau=tau[-dead.comp]
        beta[[dead.comp]] <- NULL
      }
    }
  }
  else{
    ## Birth
    tau.poss=tau.min:tau.max
    tau.not.poss=1
    for(tau.idx in 1:length(tau)){
      tau.not.poss=c(tau.not.poss,(tau[tau.idx]-tau.buffer):(tau[tau.idx]+tau.buffer))
    }
```

```
        tau.poss=tau.poss[is.element(tau.poss,tau.not.poss)==FALSE]
        if(length(tau.poss)>5){
          #tau.born=sample(tau.poss,1)
          x.idx=rep(NA,length(tau.poss))
          for(t.idx in 1:length(tau.poss)){
            x.idx[t.idx]=which.min(abs(tau.poss[t.idx]-birth.tau.dens$x))
          }
          probs=birth.tau.dens$y[x.idx]
          tau.born=tau.poss[which(rmultinom(1,1,probs)==1)]
          new.tau=c(tau,tau.born)
          new.order=order(new.tau)
          tau=new.tau[new.order]
          tau.aug=c(tau,length(z)/2+1)
          center.idx=round(.5*(tau.born+tau.aug[which(new.order==length(new.order))+1]))
          beta.born=rnorm(p,birth.beta.means[center.idx,],birth.beta.sd[center.idx,])

          beta.new=beta
          beta.new[[length(beta)]] <- beta.born
          beta=list()
          for(w in 1:length(beta.new)){
            beta[[w]] <- beta.new[[new.order[w]]]
          }
          beta[[length(beta)+1]] <- length(tau)
        }
      }
      meanvec=mv(z,Q,tau,beta)
      loglik.current=sum(dnorm(z,meanvec,sqrt(s2),log=TRUE))
      #cat("bd ",loglik.current,"\n")
    }
  }
    tot.time=proc.time()[3]-start.comp.time
  }


  ##
  ## sample CRAWL path
  ##
  keep.idx=0
  while(keep.idx==0){
    samp.new <- crwPostIS(sim.obj, fullPost=FALSE)
    path.list=cbind(samp.new$alpha.sim.x[,'mu'], samp.new$alpha.sim.y[,'mu'])
    path.loc.idx=get.path.idx(xy,path.list)
    if(min(path.loc.idx)>0 & max(path.loc.idx)<10000){
      keep.idx=1
    }
  }
  y.seal=path.list[-1,]-path.list[-dim(path.list)[1],]
  Q=get.Q(X,X.grad.x,X.grad.y,path.loc.idx)
  z=c(y.seal[-(dim(y.seal)[1]),1],y.seal[-(dim(y.seal)[1]),2])
  meanvec=mv(z,Q,tau,beta)
  loglik.current=sum(dnorm(z,meanvec,sqrt(s2),log=TRUE))
  #samp.new <- crwPostIS(sim.obj, fullPost=FALSE)
  #path.list=cbind(samp.new$alpha.sim.x[,'mu'], samp.new$alpha.sim.y[,'mu'])
  #path.loc.idx=get.path.idx(xy,path.list)
```

```
#y.seal=path.list[-1,]-path.list[-dim(path.list)[1],]
#Q=get.Q(X,X.grad.x,X.grad.y,path.loc.idx)
#z=c(y.seal[-(dim(y.seal)[1]),1],y.seal[-(dim(y.seal)[1]),2])
#meanvec=mv(z,Q,tau,beta)
#loglik.current=sum(dnorm(z,meanvec,sqrt(s2),log=TRUE))

###
### sample s2
###


tmpr=(1/r+.5*t(z-meanvec)%*%(z-meanvec))^(-1)
tmpq=n/2+q
s2=1/rgamma(1,tmpq,,tmpr)



###
### sample beta
###

#cat(tau,"\n")

#browser()
tau.aug=c(tau,length(z)/2+1)
for(k in 1:length(tau)){
  idx.current=c(tau.aug[k]:(tau.aug[k+1]-1),(length(z)/2+tau.aug[k]):(length(z)/2+tau.aug[k+1]-1))
  tmpvar=ginv(t(Q[idx.current,])%*%Q[idx.current,]/s2 + solve(Sig0))
  tmpmean=tmpvar%*%(t(Q[idx.current,])%*%z[idx.current]/s2 + solve(Sig0)%*%beta0)
  ##
  ## Keep beta the same if the matrix is singular
  ##  (this problem comes when the time interval
  ##   gets small)
  ##
  if(det(tmpvar)>10^-30){
    beta[[k]]=tmpmean+t(chol(tmpvar))%*%matrix(rnorm(p),p,1)
  }
}


#browser()

meanvec=mv(z,Q,tau,beta)
loglik.current=sum(dnorm(z,meanvec,sqrt(s2),log=TRUE))
#cat("beta ",loglik.current,"\n")

###
### MH step for tau
###

#browser()

if(length(tau)>1){
  for(k in 2:length(tau)){
```

```
      min.min=max(tau.min,tau.aug[k-1]+tau.buffer)
      t.min.dist=tau[k]-min.min
      max.max=min(tau.max,tau.aug[k+1]-tau.buffer)
      t.max.dist=max.max-tau[k]
      if(t.max.dist<=t.min.dist){
        tau.max.poss=min(max.max,tau[k]+tau.tune)
        tau.min.poss=max(min.min,tau[k]-tau.tune)
        if(tau.max.poss>tau.min.poss){
          tau.prop=sample(tau.max.poss:max((tau.max.poss-2*tau.tune),tau.min.poss),1)
        }
        else{
          tau.prop=tau[k]
        }
      }
      else{
        tau.max.poss=min(max.max-tau.buffer,tau[k]+tau.tune)
        tau.min.poss=max(min.min+tau.buffer,tau[k]-tau.tune)
        if(tau.max.poss>tau.min.poss){
          tau.prop=sample(tau.min.poss:min((tau.min.poss+2*tau.tune),tau.max.poss),1)
        }
        else{
          tau.prop=tau[k]
        }
      }
      tau.star=tau
      tau.star[k] <- tau.prop
      meanvec.star=mv(z,Q,tau.star,beta)
      mh1=sum(dnorm(z,meanvec.star,sqrt(s2),log=TRUE))
      mh2=loglik.current
      if(runif(1)<exp(mh1-mh2)){
        tau=tau.star
        tau.aug=c(tau,T+1)
        meanvec=meanvec.star
        loglik.current=mh1
        #cat("tau ",loglik.current,"\n")
      }
    }
  }



  ###
  ### save samples
  ###

  params.save[[m]] <- list(beta=beta,s2=s2,tau=tau)
  numstates.save[m] <- length(tau)

  ## backup
  #tmp.save=list(params.save=params.save,numstates.save=numstates.save,
                 n.mcmc=m,path.length=T,sample.path=path.list)
  #save(tmp.save,file="tmp.save.Rdata")
```

```
  if(show.tau==TRUE){
    cat(m," ",tau,"\n")
  }
}
#cat("\n")

list(params.save=params.save,numstates.save=numstates.save,n.mcmc=n.mcmc,n=n,
p=p,sample.path.loc.idx=path.loc.idx,sample.path=path.list,
path.length=dim(path.list)[1],datetime=datetime)

}
```
