# Supplementary material for: Velocity-Based Movement Modeling for Individual and Population Level Inference
Source: PLoS One. 2011 Aug 11;6(8):e22795. doi: 10.1371/journal.pone.0022795 (PMC3154913; doi:10.1371/journal.pone.0022795)
Supplement: Text S2 — Simulation Study. This supplement contains a simulation study illustrating our approach to individual and population-level inference on animal movement. (PDF) [file pone.0022795.s002.pdf]

Supporting Information S2 - Simulation Study

1 Overview

To verify our approach for modeling individual and population-level movement and resource selection, we conducted a simulation study. We make the following assumptions about animal behavior:

1. At the population level, patterns of animal movement and response to the environment can be partitioned into a fixed number of regimes, each of which can be represented by a distinct pattern of correlation to gradients of environmental or biotic covariates.

2. Sub-groups of the population may have different propensities for each of these movement regimes, manifested by differences in the proportion of use of the regimes accross subgroups.

3. Individual animals may have varying propensities for each of the movement regimes as well, manifested by differences in the proportion of use of the regimes accross individuals.

4. Individual animal movement in an observation period can be modeled by breaking the path into an unknown (random) number of partitions. In each of these partitions, animal movement and resource selection follows one of the population-level regimes.

2 Simulation Study

2.1 Population-Level Regimes

We consider a population with three major patterns of movement and selection, as manifested by correlation with gradients of two covariates. We represent each of these regimes by a multivariate Gaussian distribution with a mean representing the average movement response to the covariates for animals when in this movement regime (Table 1). The distributions of these regimes were chosen in such a way that there is considerable potential overlap between them (Figure 1). In this way we can guage the ability of our approach to detect movement patterns that may not be completely distinct.

Regime 1 is centered at the origin, indicating a pattern of little movement and little response to covariate gradients. Regime 2 is centered at (3,-2), indicating a movement state in which animals move predominantly in directions corresponding to increasing levels of covariate 1 and decreasing levels of covariate 2. Regime 3 is centered at (0,2), indicating a movement state in which animals move predominantly in directions corresponding to increasing levels of covariate 2.

Table 1. Population-Level Movement Regimes

Regime	Mean	Standard Deviation
1	(0,0)	2
2	(3,-2)	2
3	(0,2)	5

2.2 Differences in Population Sub-Groups

We consider two subgroups of the population: male and female. Differences in subgroup movement and selection are manifested by different preferences for the three movement regimes. We have specified

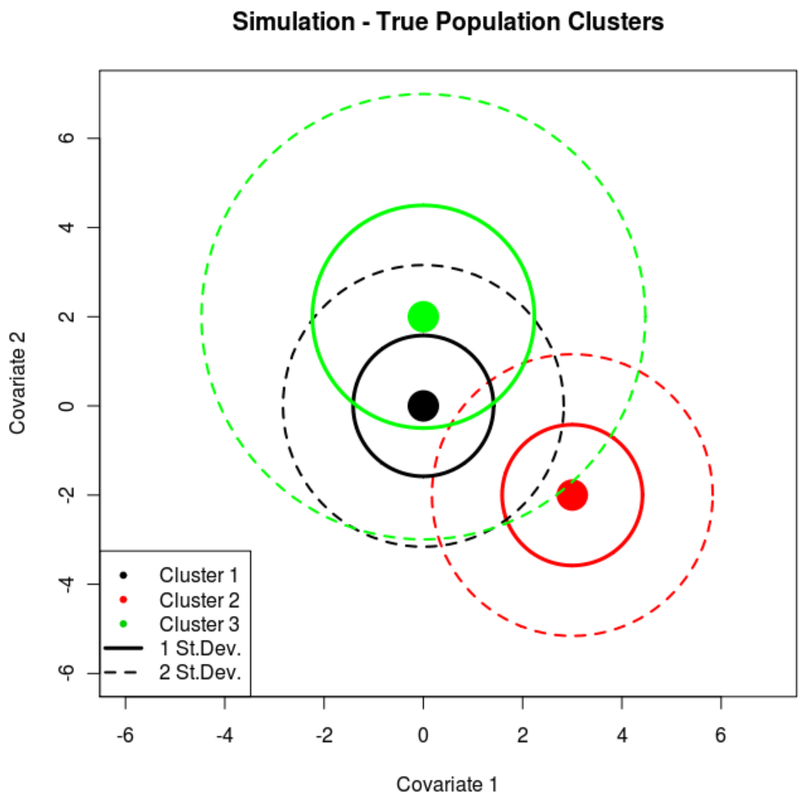

Figure 1. Population-level Movement Regimes.

different preferences in such a way that movement regime 2 is a predominantly female mode of movement and selection, regime 3 is a predominantly male mode of movement, though the difference is not as distinct as in regime 2, and regime 1 is used slightly more by males than by females (Table 2).

2.3 Individual Animals

For this simulation study, we consider 40 animals from the population, 20 male and 20 female. Each individual animal's actual proportion of time spent in each of the 3 movement regimes over the observation period is simulated from a dirichlet distribution with mean proportion for each subgroup as specified in Table 2. The resulting proportions of time in each regime are shown in Table 3.

2.4 Simulating Movement

Our approach assumes that animal movement is correlated with observed covariates. Let $\mathbf{y}_{it} = (y_{it1}, y_{it2})$ be the vector-valued velocity of the i-th animal at time t. Thus $\mathbf{y}_{it}$ is a vector representing the movement step made by the animal between time t and time $t + 1$. The correlation with observed covariates is modeled in a regression format, with covariate gradients as predictors and regression parameters $\boldsymbol{\beta}_{it}$ coming from the movement regime the animal is in at time t.

For each animal, we simulated a path of 1000 time steps in the following way.

Table 2. Sub-Group Utilization of Population-Level Movement Regimes

Sub-Group	Average Proportion Of Time In Regime 1	Average Proportion Of Time In Regime 2	Average Proportion Of Time In Regime 3
Male	.3	.1	.6
Female	.2	.5	.3

- The observation period of 1000 time steps was divided up into partitions, each associated with a movement regime, in such a way that the individual animal's proportion of time spent in each regime is as shown in Table 3.
- For each time step, values of $\boldsymbol{\beta}_{it}$ are drawn from the multivariate Gaussian distribution (Table 1) specified by the movement regime the animal is in at time t.
- At each time step, covariate gradient values $\mathbf{Q}_{it}$ are drawn from normal distributions with mean 0 and variance 1. This allows for varying levels of covariate gradients accross the observation period of the animal.
- The movement step $\mathbf{y}_{it}$ is then simulated from a normal distribution:

$$\mathbf{y}_{it} \sim N(\mathbf{Q}_{it}\boldsymbol{\beta}_{it}, \sigma^2\mathbf{I}).$$

2.5 Simulating Imperfect Observation

Telemetry data is not typically observed at regular intervals with fine temporal resolution. In our approach, we advocate accounting for uncertainty in the true, continuous movement path of an animal through use of a mechanistic, stochastic model of animal movement. This is done through sampling a new path from the posterior predictive distribution of the movement paths at each iteration of the model fitting procedure. To simulate this uncertainty in the true movement path, at each iteration of the model fitting algorithm, we simulate a new set of covariate gradients $\mathbf{Q}_{it}$, which lead to a new movement step $\mathbf{y}_{it}$. In this way, each iteration of the fitting algorithm uses a different simulated movement path.

2.6 Individual and Population-Level Inference

Our approach for making inference on individual and population-level movement and resource selection can be summarized as follows:

1. Make inference about drivers of movement for each individual animal using the velocity-based movement approach. This approach accounts for the uncertainty in the movement path distribution and allows for temporal heterogeneity in the drivers of movement.
2. Aggregate all values of $\boldsymbol{\beta}_{i,t}$, the effects of the drivers of movement for all animals and at all times and cluster these aggregated effects in parameter space.
3. Make inference about differences in how population subsets respond to drivers of movement by using the proportion of time spent by individual animals in each movement cluster as predictor variables. This could be done using parametric (e.g., logistic regression) or nonparametric (e.g., classification trees) statistical methods.

Table 3. Individual Proportions of Time Spent in Each Population-Level Movement Regime

Male	Proportion Of Time In Regime 1	Proportion Of Time In Regime 2	Proportion Of Time In Regime 3	Female	Proportion Of Time In Regime 1	Proportion Of Time In Regime 2	Proportion Of Time In Regime 3
1	0.55	0.05	0.41	1	0.03	0.86	0.10
2	0.88	0.00	0.12	2	0.00	0.05	0.95
3	0.58	0.20	0.22	3	0.95	0.05	0.00
4	0.02	0.00	0.98	4	0.00	0.87	0.12
5	0.07	0.00	0.93	5	0.83	0.00	0.17
6	0.61	0.36	0.03	6	0.00	0.06	0.94
7	0.28	0.00	0.72	7	0.45	0.00	0.55
8	0.04	0.00	0.96	8	0.89	0.11	0.00
9	0.86	0.13	0.00	9	0.51	0.49	0.00
10	0.98	0.00	0.02	10	0.01	0.43	0.56
11	0.69	0.30	0.01	11	0.02	0.07	0.92
12	0.23	0.00	0.77	12	0.00	0.91	0.09
13	0.22	0.00	0.78	13	0.01	0.97	0.03
14	0.10	0.00	0.90	14	0.00	0.05	0.95
15	0.41	0.00	0.59	15	0.00	0.02	0.98
16	0.03	0.76	0.21	16	0.02	0.96	0.02
17	0.03	0.74	0.24	17	0.00	0.05	0.95
18	0.39	0.00	0.61	18	0.01	0.74	0.24
19	0.00	0.00	1.00	19	0.04	0.96	0.00
20	0.00	0.01	0.99	20	0.13	0.87	0.00

3 Population-Level Results

The population-level cluster analysis of the $\boldsymbol{\beta}_{i,t}$ from all 40 animals in the simulated population finds four clusters to be the best fit to the data. Figure 2 shows the $\boldsymbol{\beta}_{i,t}$ used in the cluster analysis, with colors indicating which of the four clusters they were assigned to. Clusters 1-3 correspond roughly with the population-level movement regimes 1-3, while cluster 4 is positioned between the other three clusters. Cluster 4 is the least prevalent of the clusters, and may represent $\boldsymbol{\beta}_{i,t}$ values that are near change points in the animal's behavior.

A classification tree with sex as the dependent variable and proportion of time spent in each of the 4 movement clusters was fit, and the results (Figure 3) show that females in our simulated population spend a greater proportion of time in movement cluster 2 than do males. Cluster 2 corresponds to population regime 2, which had the highest discrepancy in proportional use between male and female animals, with females spending, on average, half of their time in that regime, while males on average spend only 10% of their time in that regime.

From this study, it is clear that our approach can be used to identify population-level patterns of movement, and large discrepancies in proportional use of those patterns of movement between subgroups of the population.

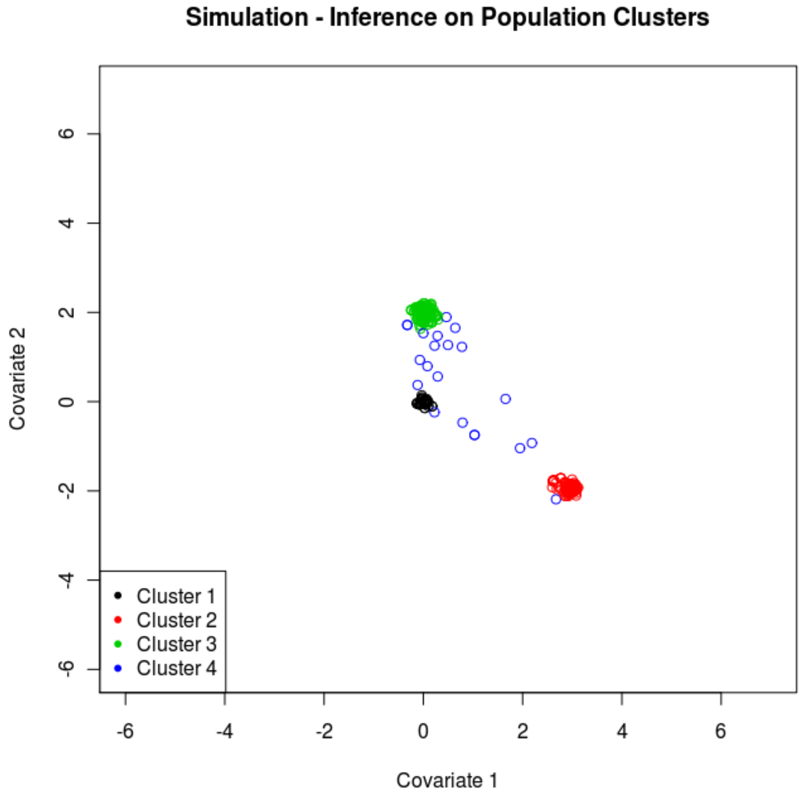

Figure 2. Inference on Population-Level Movement Clusters.

4 Additional Individual Simulations

In addition to the full simulation study above, we conducted multiple individual-level simulations to ascertain the ability of our individual approach to correctly identify movement and selection behavior that is changing over time. We again used 2 simulated covariates, but instead of fixing population-level movement regimes, we specified various patterns for the individual parameter values over time. These patterns vary from simple (one changepoint) to complex (highly periodic). The true and inferred parameter values are shown in Figures 3-6.

These results lead us to some conclusions about our individual-level approach for animal movement and resource selection.

1. The birth-death Markov chain Monte Carlo (BDMCMC) process used to make inference on time-varying parameters is able to pick up on true change points (Figures 3-4) very well.

2. When the true underlying parameters are smoothly-varying, our approach is able to make valid inference (Figures 5-6), though the BDMCMC process seems to be less effective at the endpoints of the observation period.

3. When the underlying parameters are varying quite rapidly in relation to the total time in the observation period (Figure 7), our approach is able to pick up the underlying trend in the mean,

though statistical power is lost, and the 95% credible intervals for the parameters are likely to overlap zero.

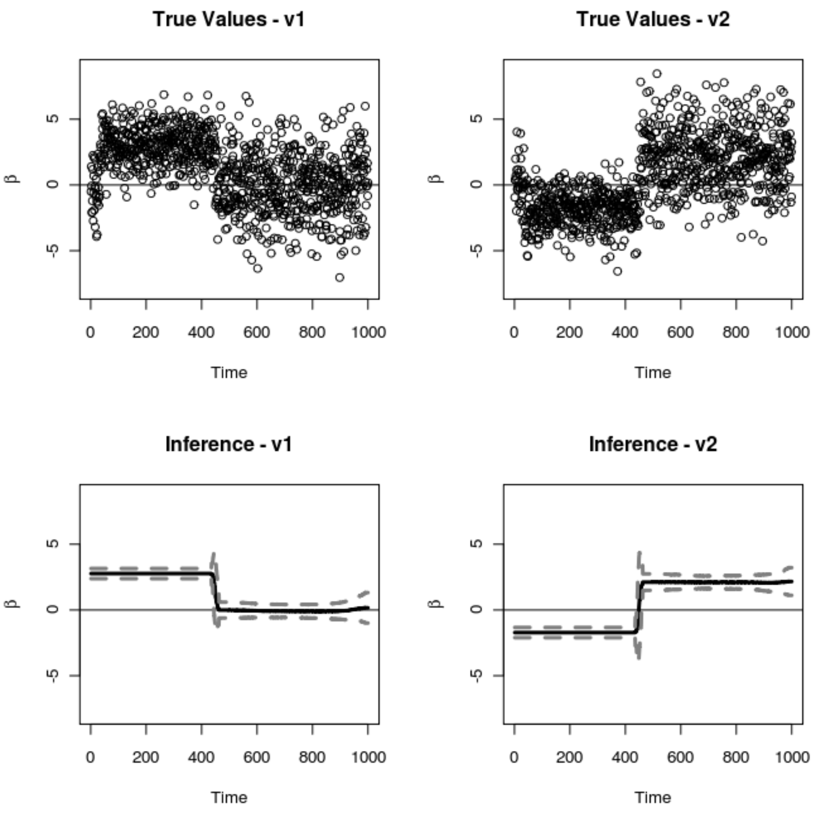

Figure 3. One Change Point

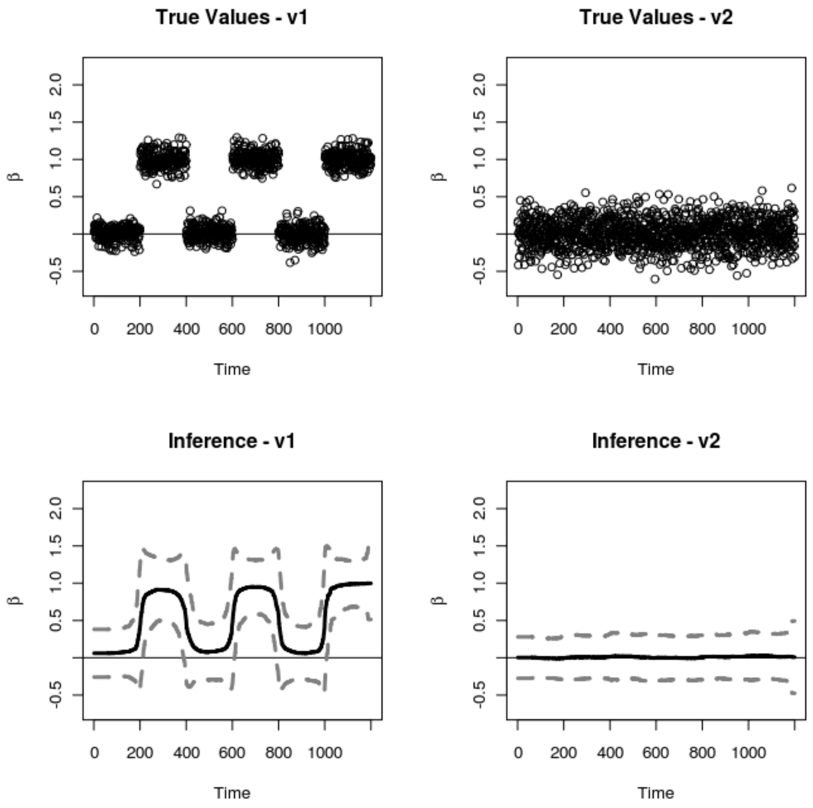

Figure 4. Multiple Change Points

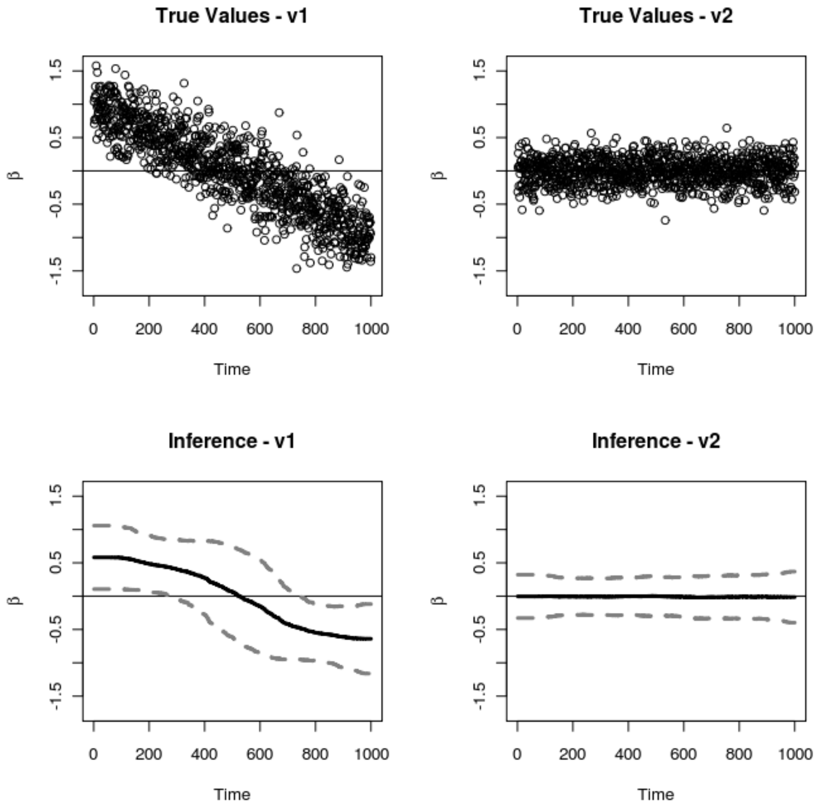

Figure 5. Linear Trend in One Variable

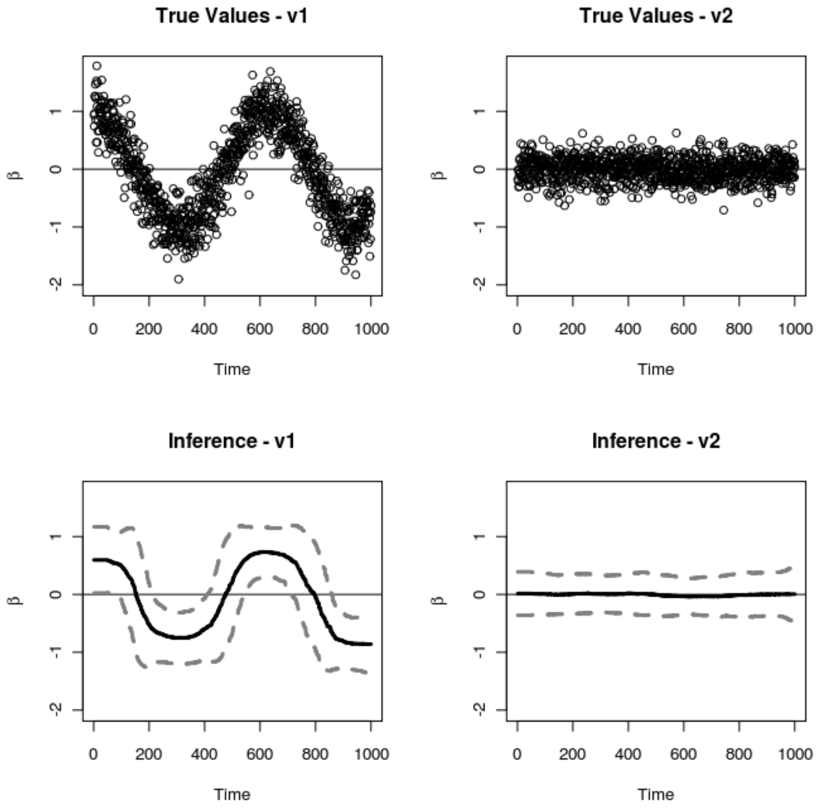

Figure 6. Sinusoidal Trend, Long Period

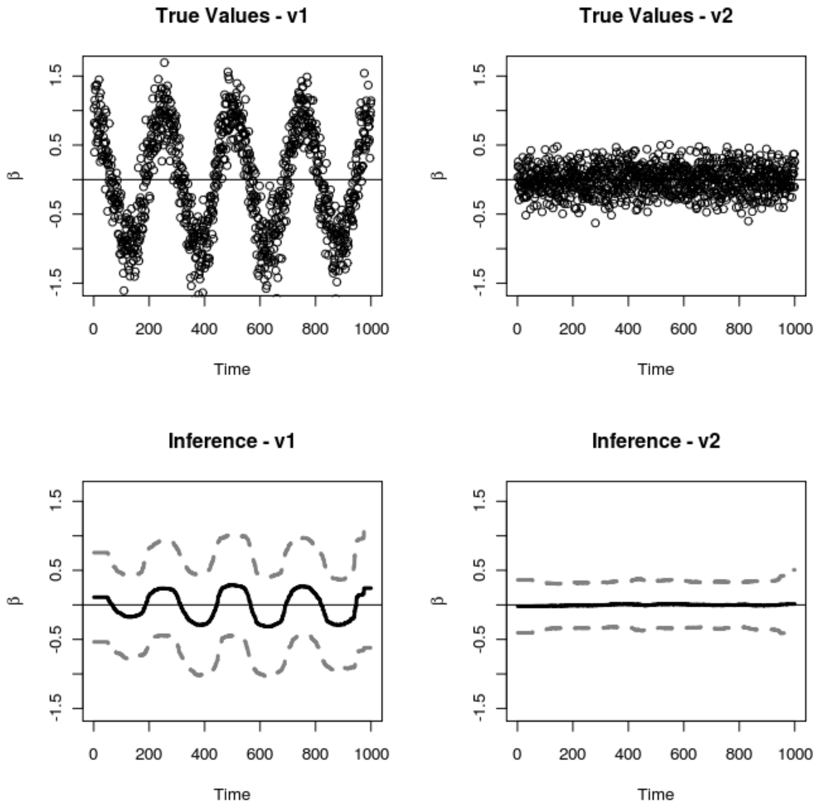

Figure 7. Sinusoidal Trend, Short Period
