# Supplementary material for: Velocity-Based Movement Modeling for Individual and Population Level Inference
Source: PLoS One. 2011 Aug 11;6(8):e22795. doi: 10.1371/journal.pone.0022795 (PMC3154913; doi:10.1371/journal.pone.0022795)
Supplement: Text S3 — Individual-Level Results. This supplement contains plots of individual-level results for all animals. (PDF) [file pone.0022795.s003.pdf]

Supporting Information S3 - Additional Results

Overview

Here we present additional results of the individual and population-level study of northern fur seals (NFS). Section 1 contains plots of $\boldsymbol{\beta}_{i,t}$ for the two animals in our focused individual study for all models listed in Table 1. Section 2 contains results for $\boldsymbol{\beta}_{i,t}$ in the full model for all animals and trips not shown in the manuscript. The temporally-varying nature of the $\boldsymbol{\beta}_{i,t}$, and the variation in path length, observation window, and spatial locations between the northern fur seals in the study make it difficult to tabulate results. The plots in Section 2 show the time varying $\boldsymbol{\beta}_{i,t}$, and the population-level movement clusters that each time-point on each path corresponds to. Finally, in Section 3 we have included example trace plots for the $\boldsymbol{\beta}_{i,t}$ that illustrate convergence of the MCMC algorithm used in our individual-level approach.

1 Results for Animal 1 and Animal 2 - Model Comparison

Animal 1 - Model 2

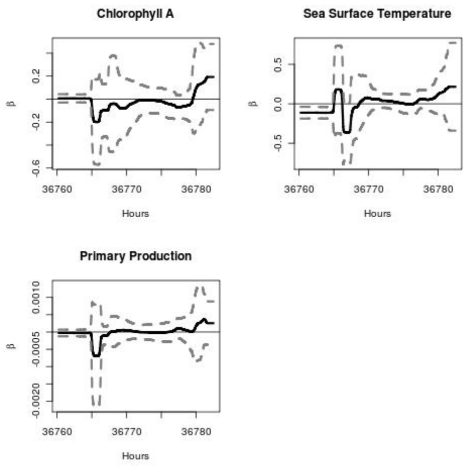

Animal 1 - Model 3

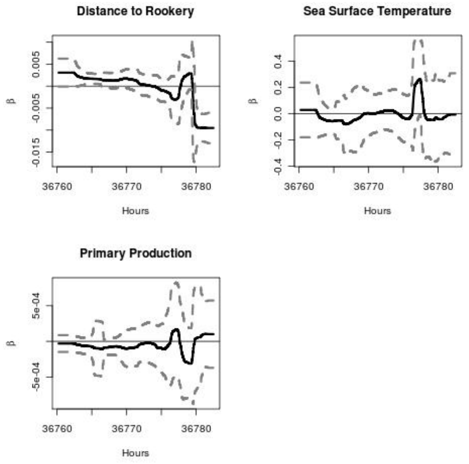

Animal 1 - Model 4

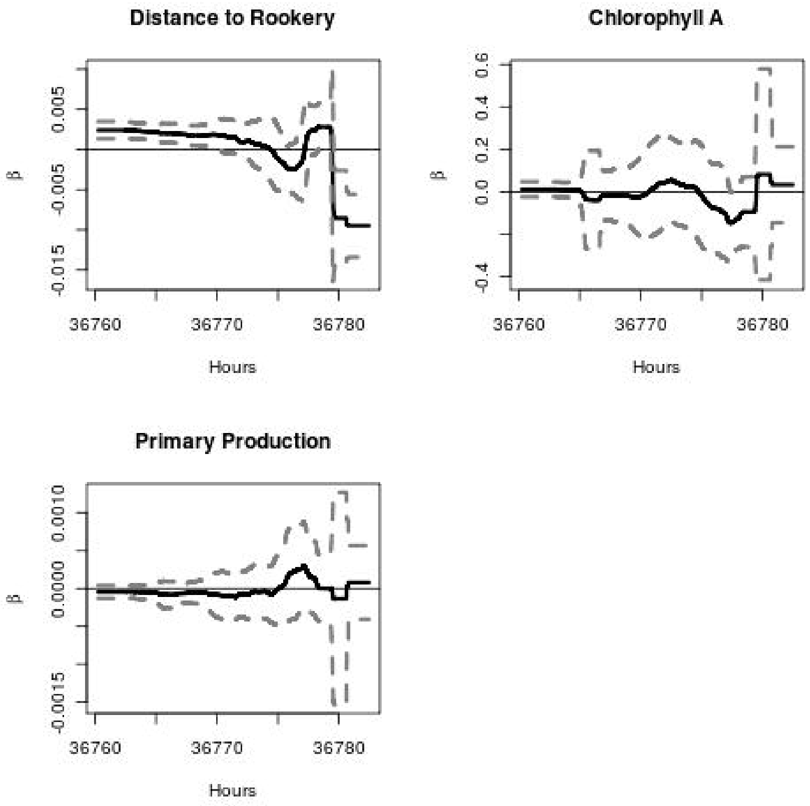

Animal 1 - Model 5

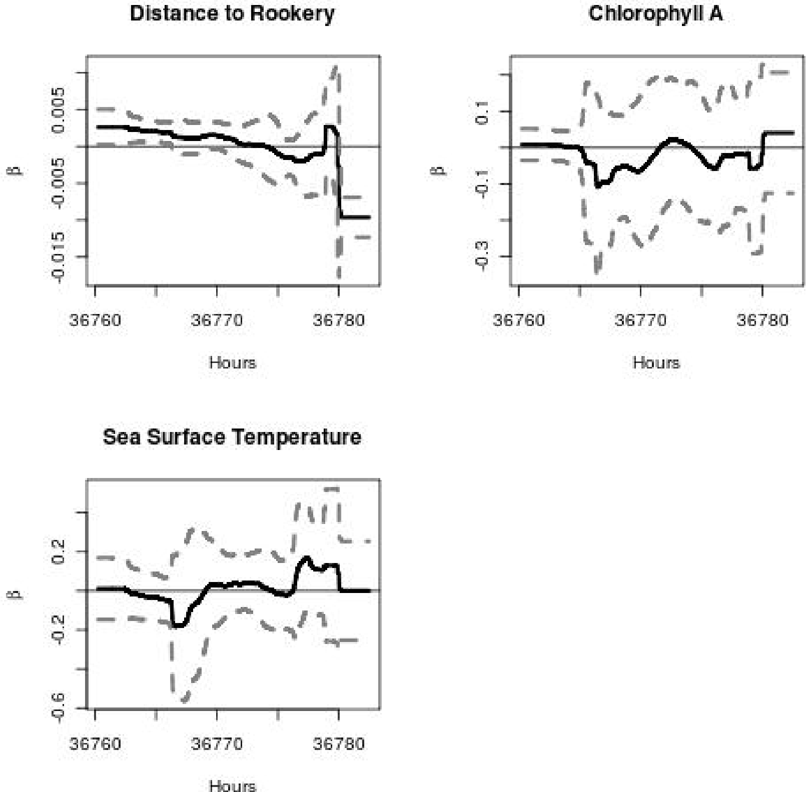

Animal 1 - Model 6

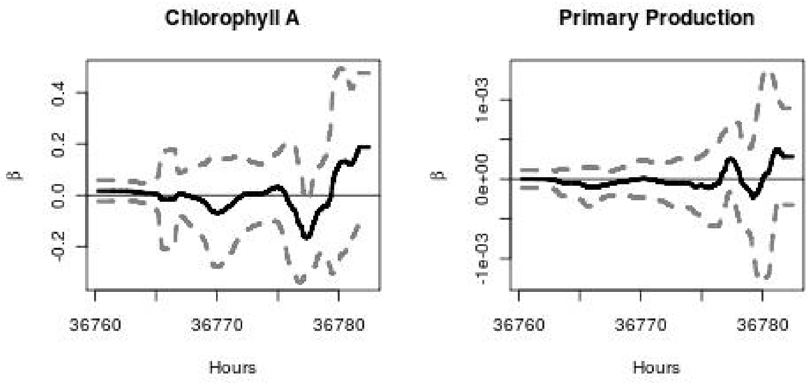

Animal 1 - Model 7

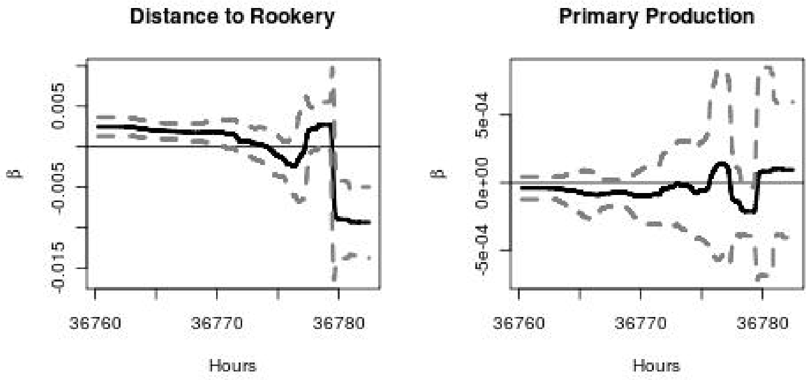

Animal 1 - Model 8

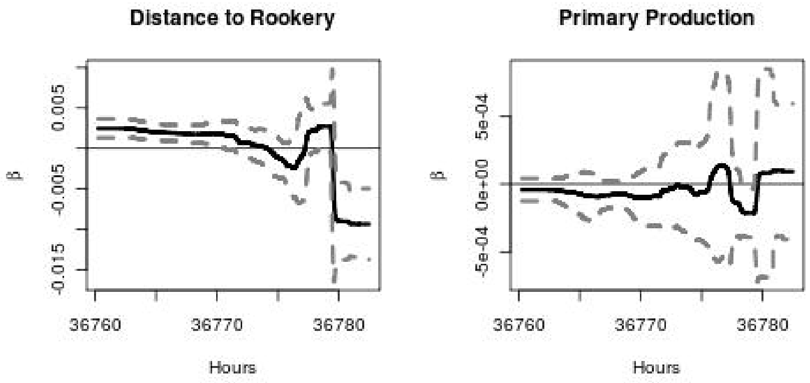

Animal 1 - Model 9

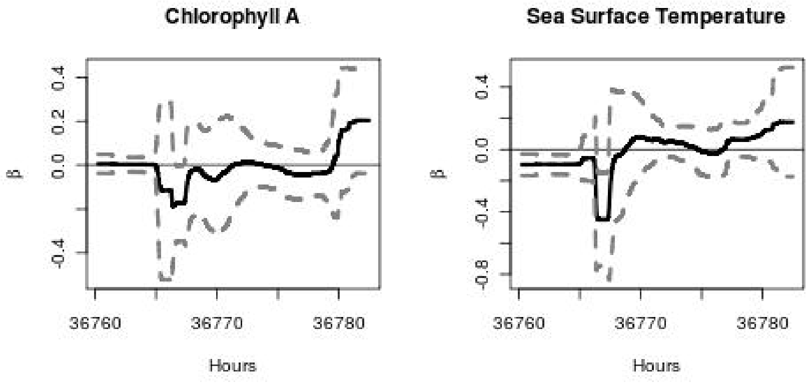

Animal 1 - Model 10

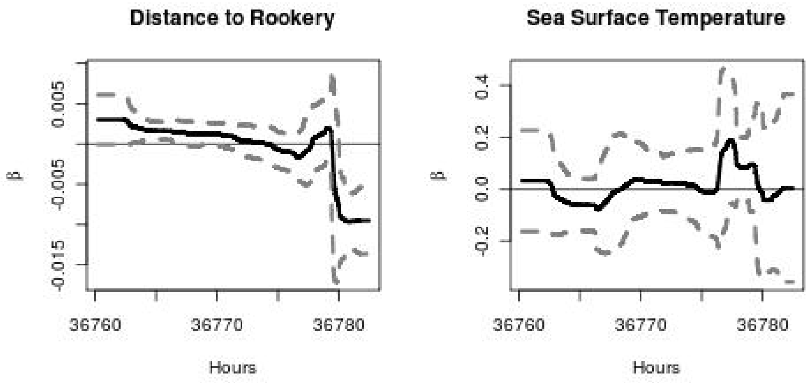

Animal 1 - Model 11

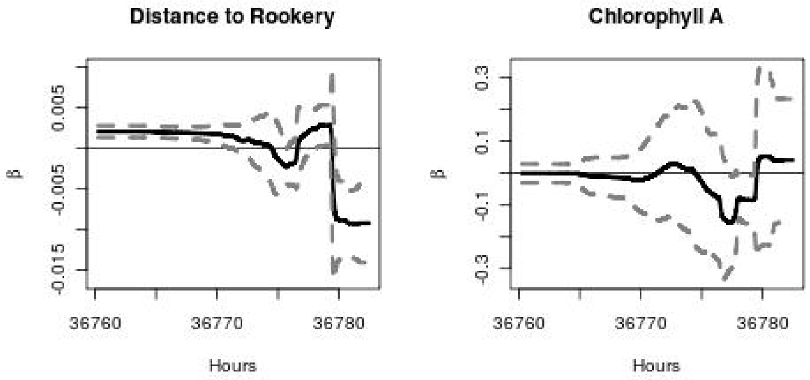

Animal 1 - Model 12

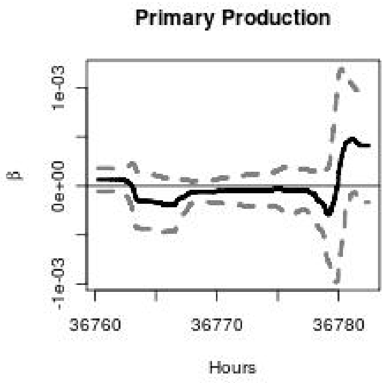

Animal 1 - Model 13

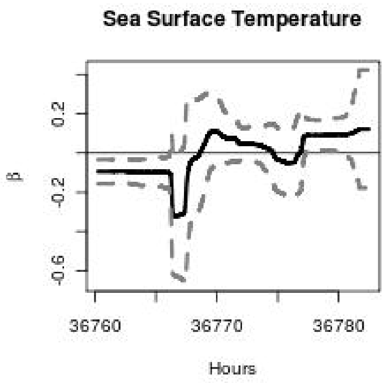

Animal 1 - Model 14

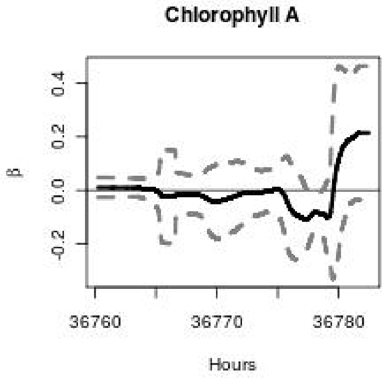

Animal 1 - Model 15

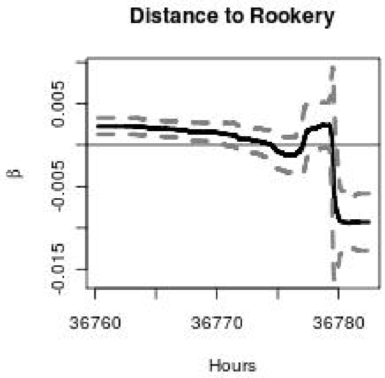

Animal 2 - Model 2

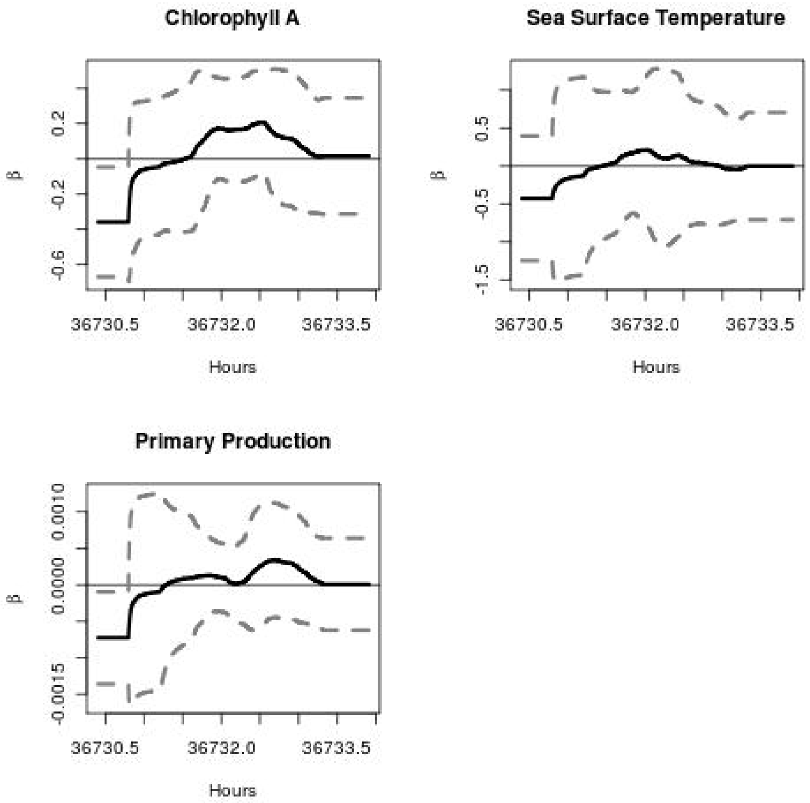

Animal 2 - Model 3

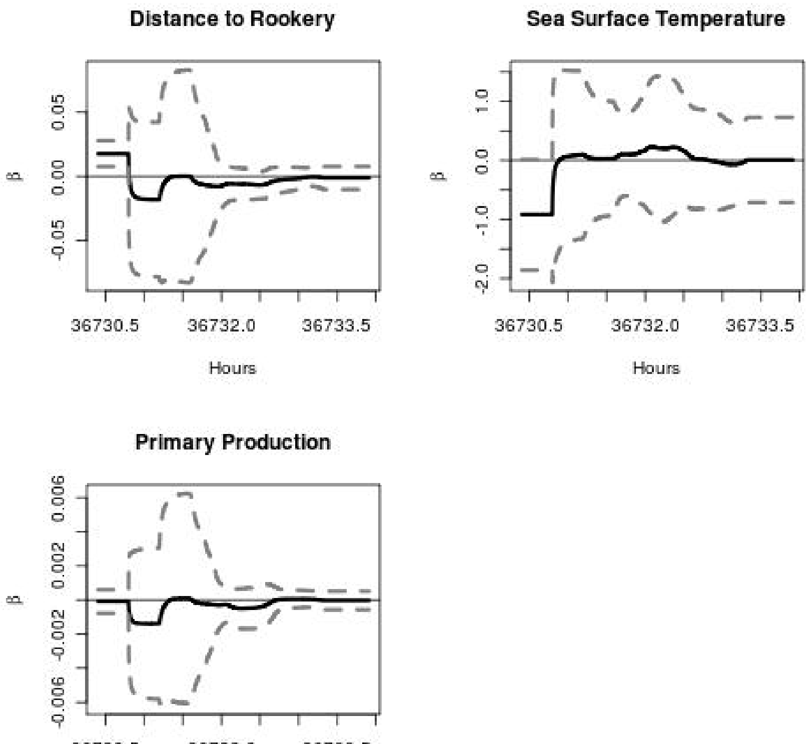

Animal 2 - Model 4

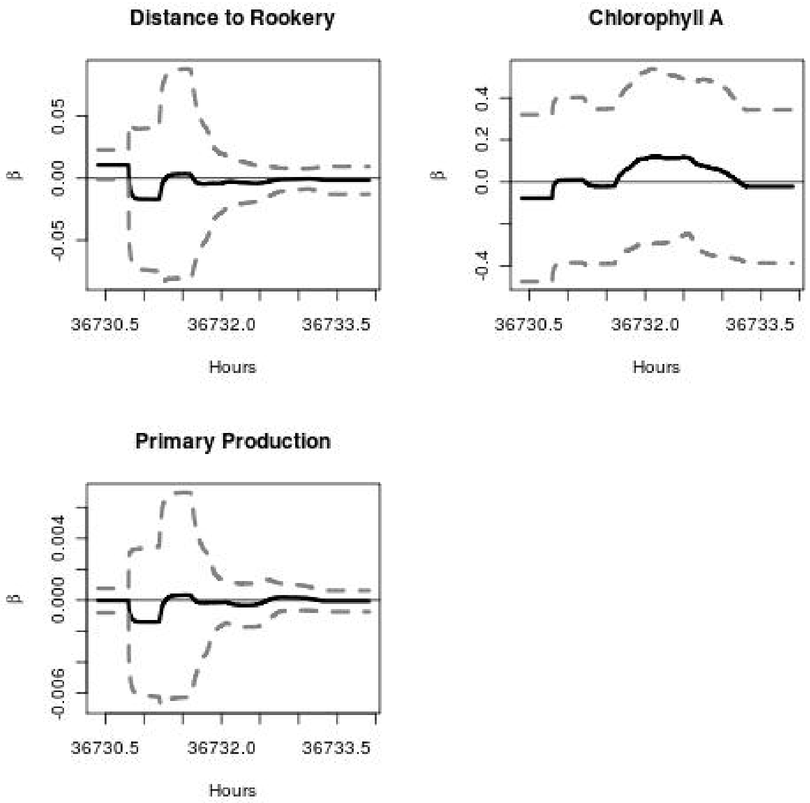

Animal 2 - Model 5

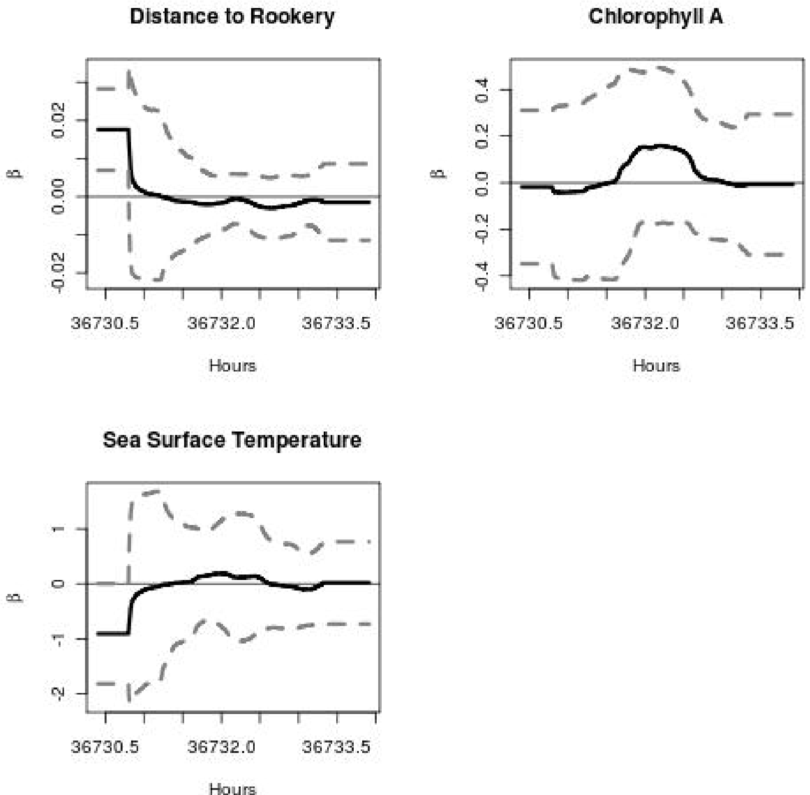

Animal 2 - Model 6

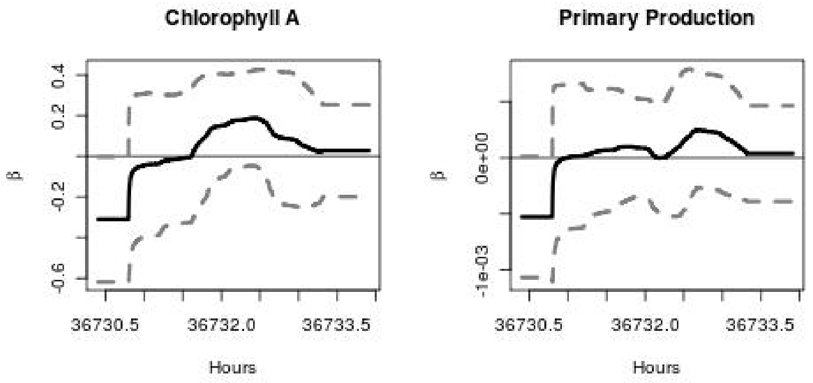

Animal 2 - Model 7

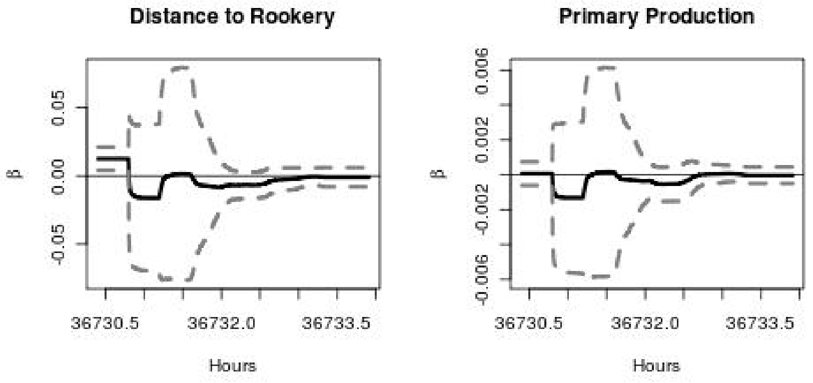

Animal 2 - Model 8

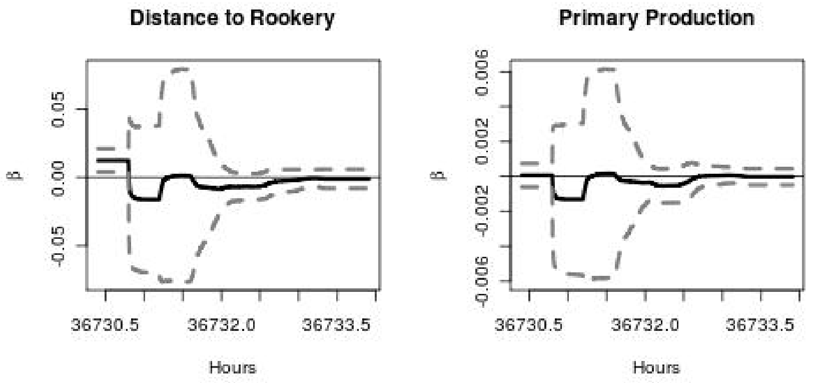

Animal 2 - Model 9

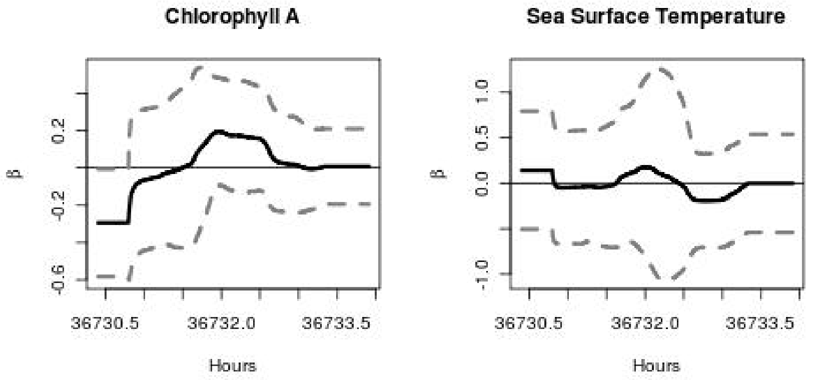

Animal 2 - Model 10

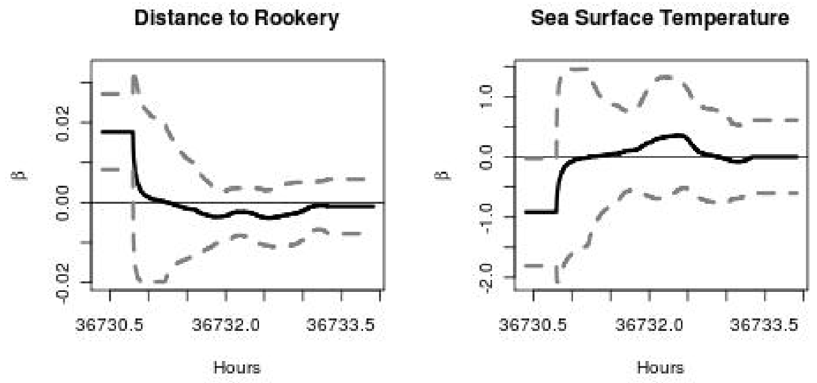

Animal 2 - Model 11

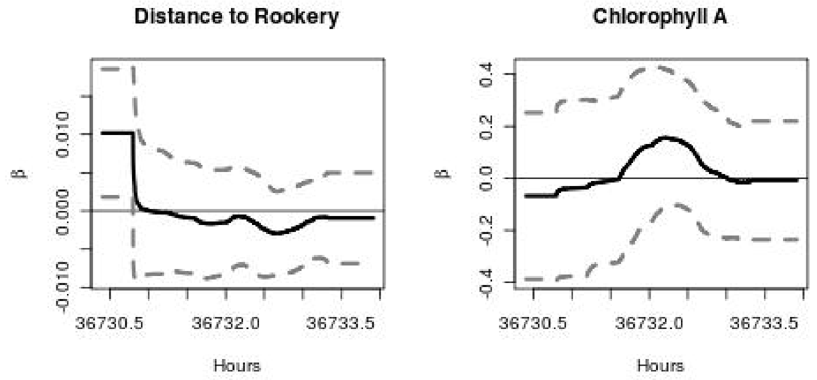

Animal 2 - Model 12

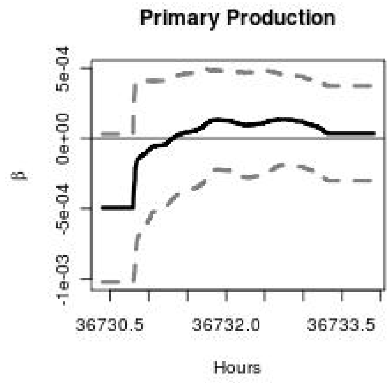

Animal 2 - Model 13

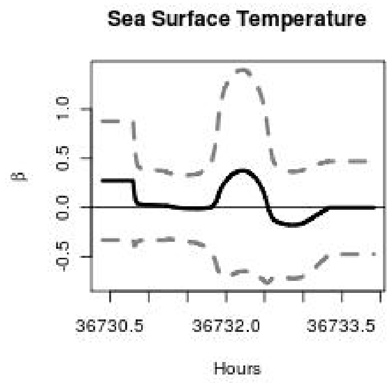

Animal 2 - Model 14

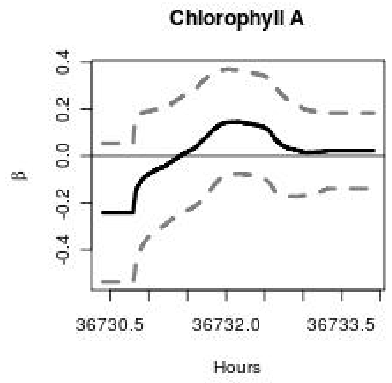

Animal 2 - Model 15

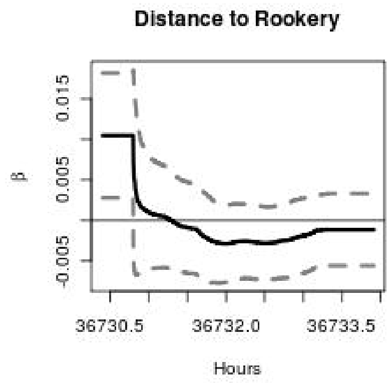

2 Full-Model Individual Results for All Animals Not Shown In Paper

Animal Index	Number of Trips	Year Captured	Sex	Tag Number	Notes
1	1	2000	M	173	Plots shown in manuscript.
2	2	2000	F	148	Plots for one trip shown in manuscript.
3	1	1999	M	136	
4	1	1999	M	137	
5	1	1999	M	138	
6	1	1999	M	139	
7	3	1999	M	140	Two trips did not converge (BDMCMC).
8	1	1999	M	141	
9	1	1999	M	142	Did not converge (BDMCMC).
10	1	1999	M	143	
11	1	1999	M	144	
12	1	1999	M	145	
13	1	1999	M	135	
14	2	2000	F	149	
15	4	2000	F	150	Two trips did not converge (CRAWL).
16	2	2000	F	151	
17	1	2000	M	152	
18	4	2000	F	153	
19	1	2000	M	154	
20	1	2000	M	156	
21	1	2000	M	157	
22	1	2000	M	158	
23	1	2000	M	159	
24	1	2000	M	160	
25	1	2000	M	161	
26	2	2000	F	162	
27	1	2000	F	163	
28	2	2000	F	164	
29	4	2000	F	165	Two trips did not converge (CRAWL).
30	1	2000	F	166	
31	1	2000	M	167	
32	1	2000	M	168	
33	1	2000	F	169	
34	1	2000	F	170	Did not converge (BDMCMC).
35	1	2000	M	171	Did not converge (BDMCMC).
36	1	2000	M	172	
37	1	1999	M	134	
38	2	2000	F	174	
39	3	2000	F	175	
40	1	2000	M	176	Did not converge (CRAWL).
41	1	2000	M	177	
42	1	2000	F	178	
43	1	2000	F	179	
44	1	2000	F	180	
45	1	1999	M	146	

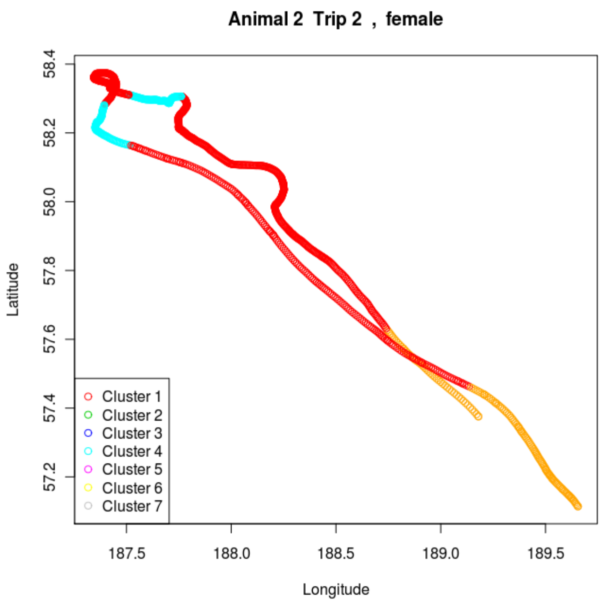

Animal 2 Trip 2 , female
Latitude
Longitude
Cluster 1
Cluster 2
Cluster 3
Cluster 4
Cluster 5
Cluster 6
Cluster 7

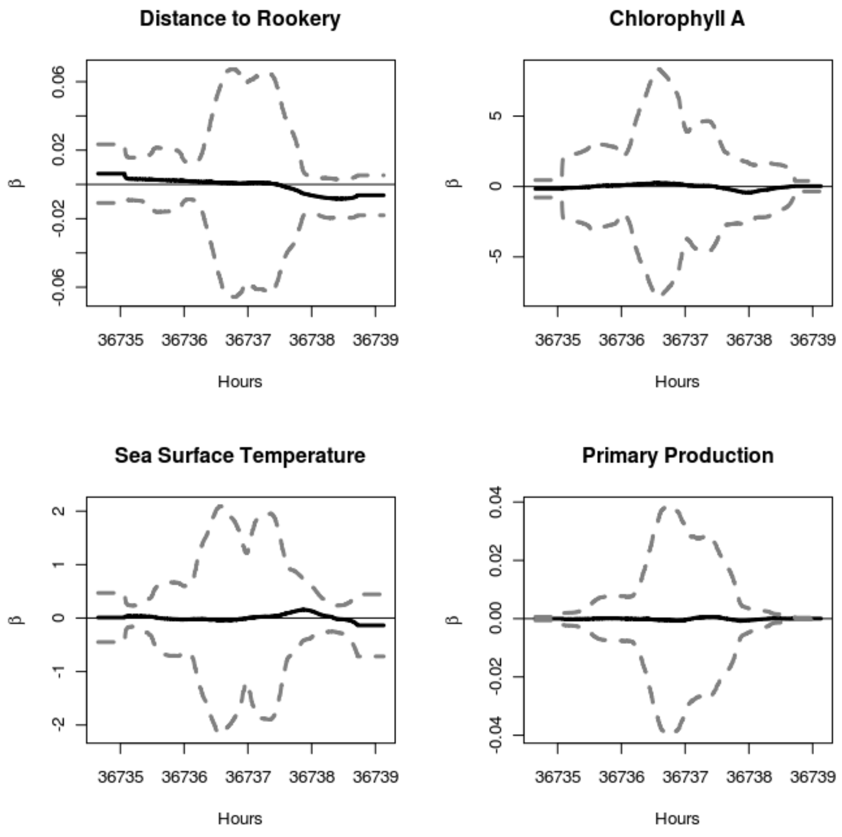

Distance to Rookery
Chlorophyll A
Sea Surface Temperature
Primary Production
β
Hours

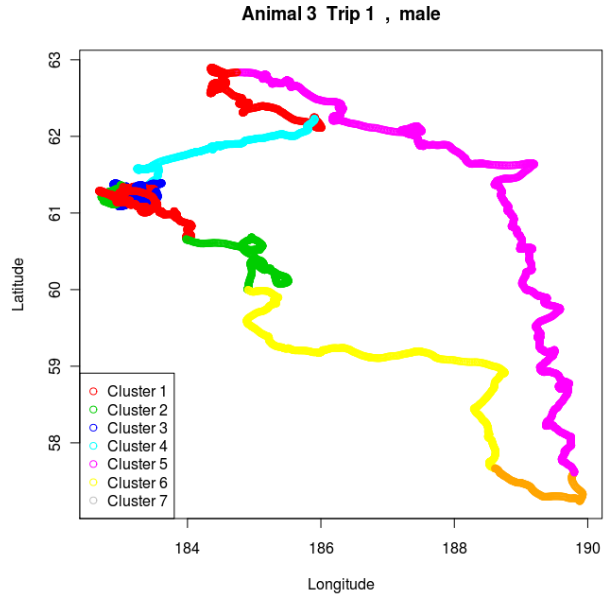

Animal 3 Trip 1 , male
Latitude
Longitude
Cluster 1
Cluster 2
Cluster 3
Cluster 4
Cluster 5
Cluster 6
Cluster 7

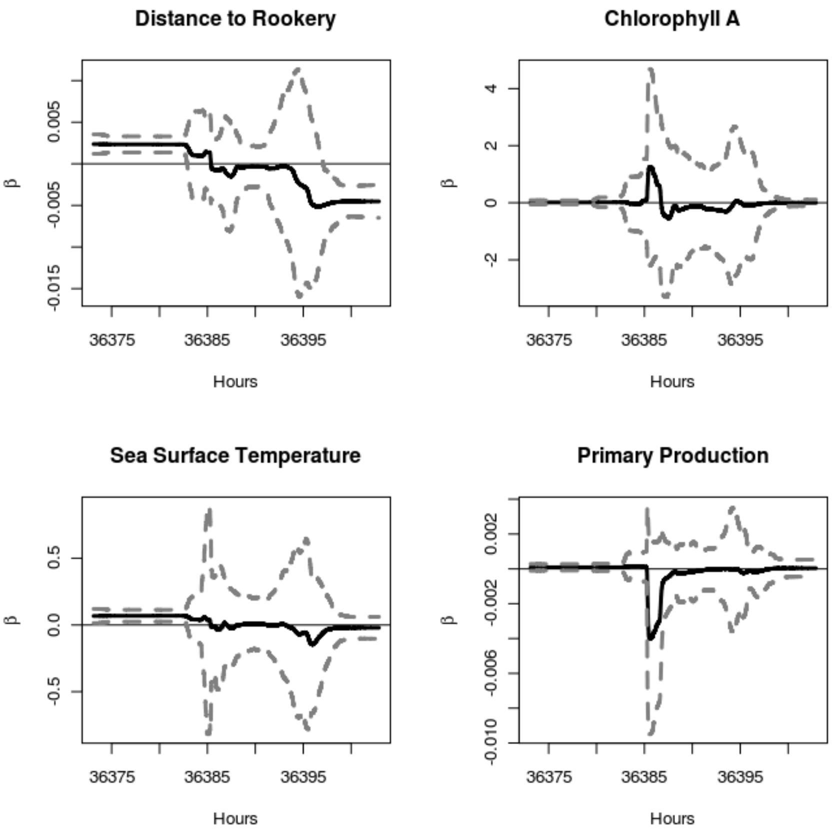

Distance to Rookery
Chlorophyll A
Sea Surface Temperature
Primary Production
β
Hours

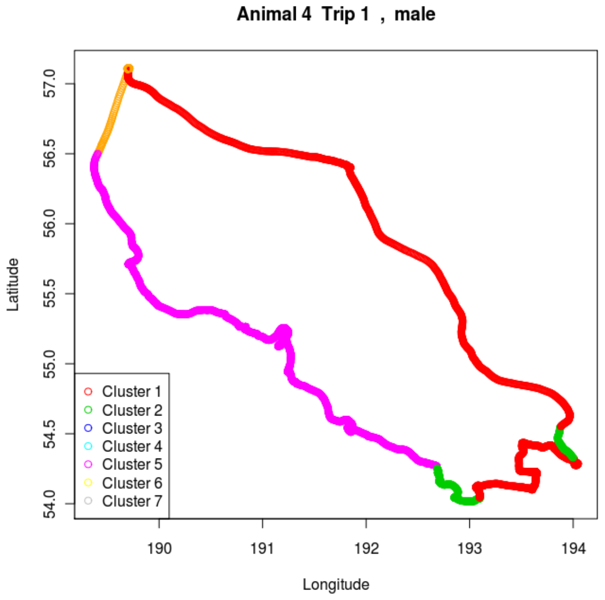
Animal 4 Trip 1 , male
Latitude
Longitude
Cluster 1
Cluster 2
Cluster 3
Cluster 4
Cluster 5
Cluster 6
Cluster 7

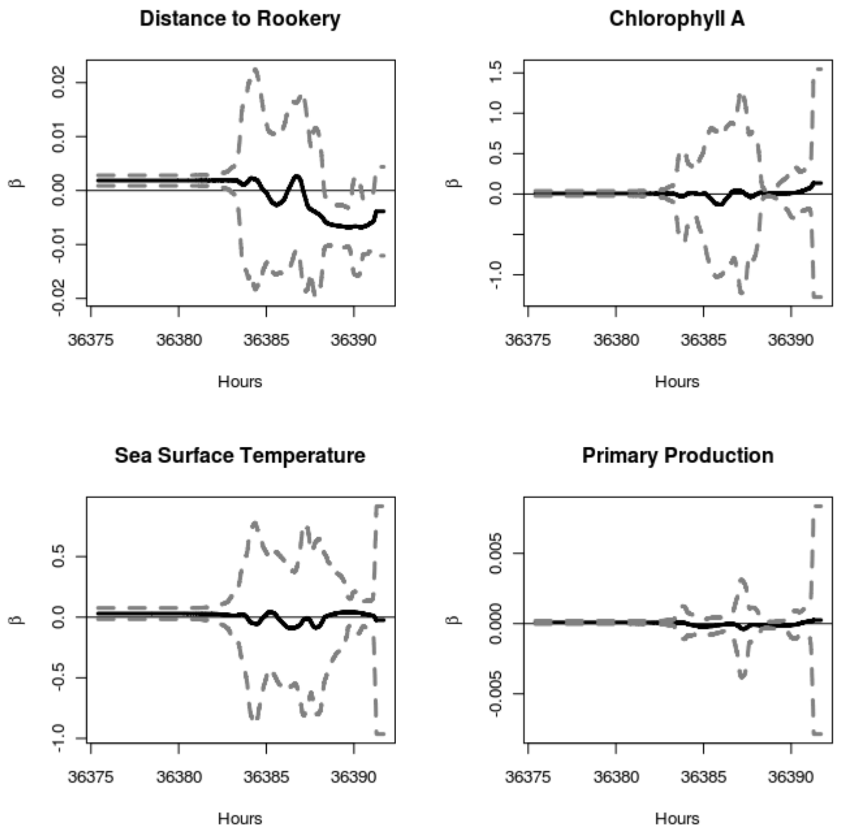
Distance to Rookery
Chlorophyll A
Sea Surface Temperature
Primary Production
β
Hours

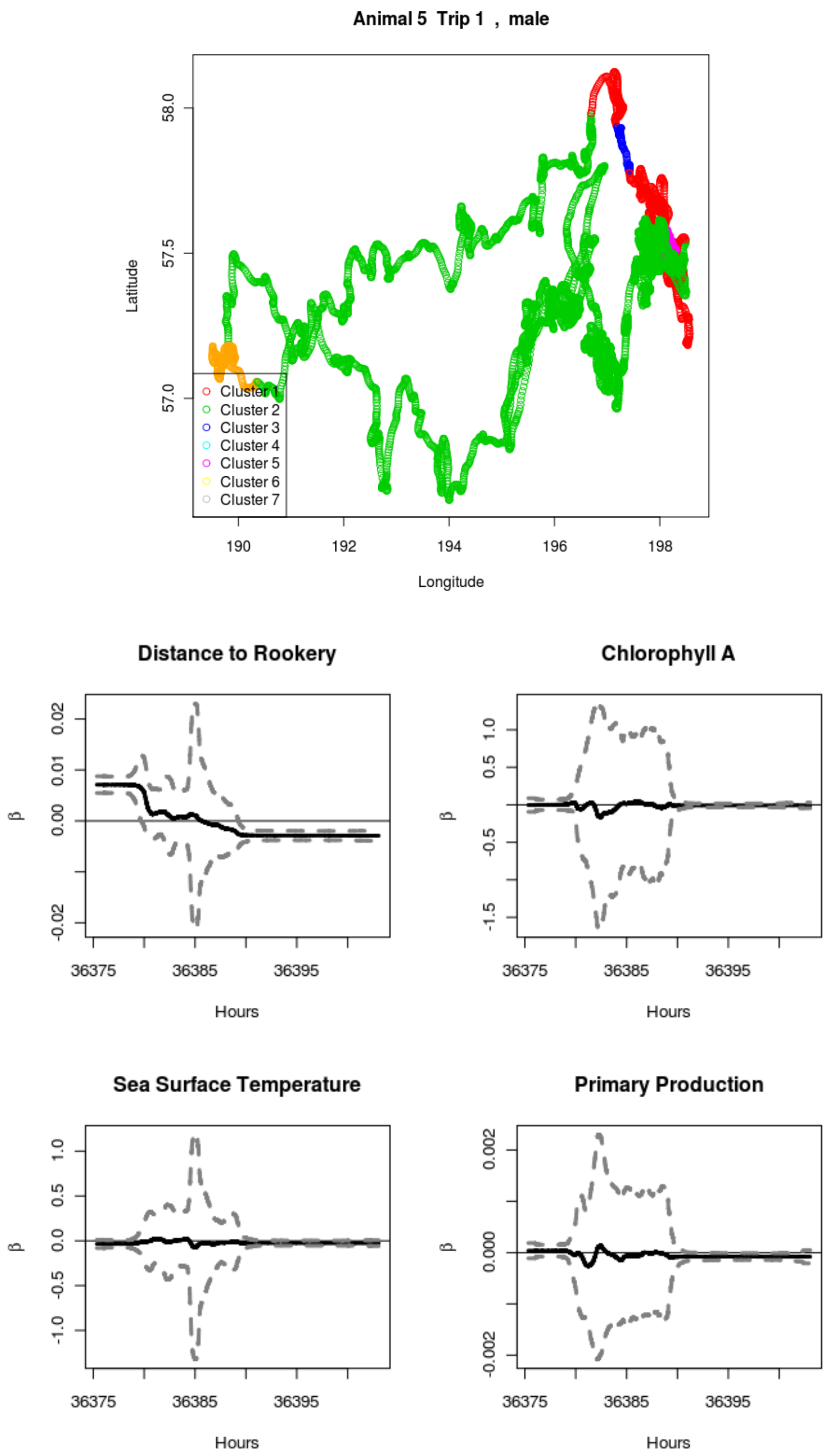
Animal 5 Trip 1 , male
Latitude
Longitude
Cluster 1
Cluster 2
Cluster 3
Cluster 4
Cluster 5
Cluster 6
Cluster 7
Distance to Rookery
Chlorophyll A
Sea Surface Temperature
Primary Production
β
Hours

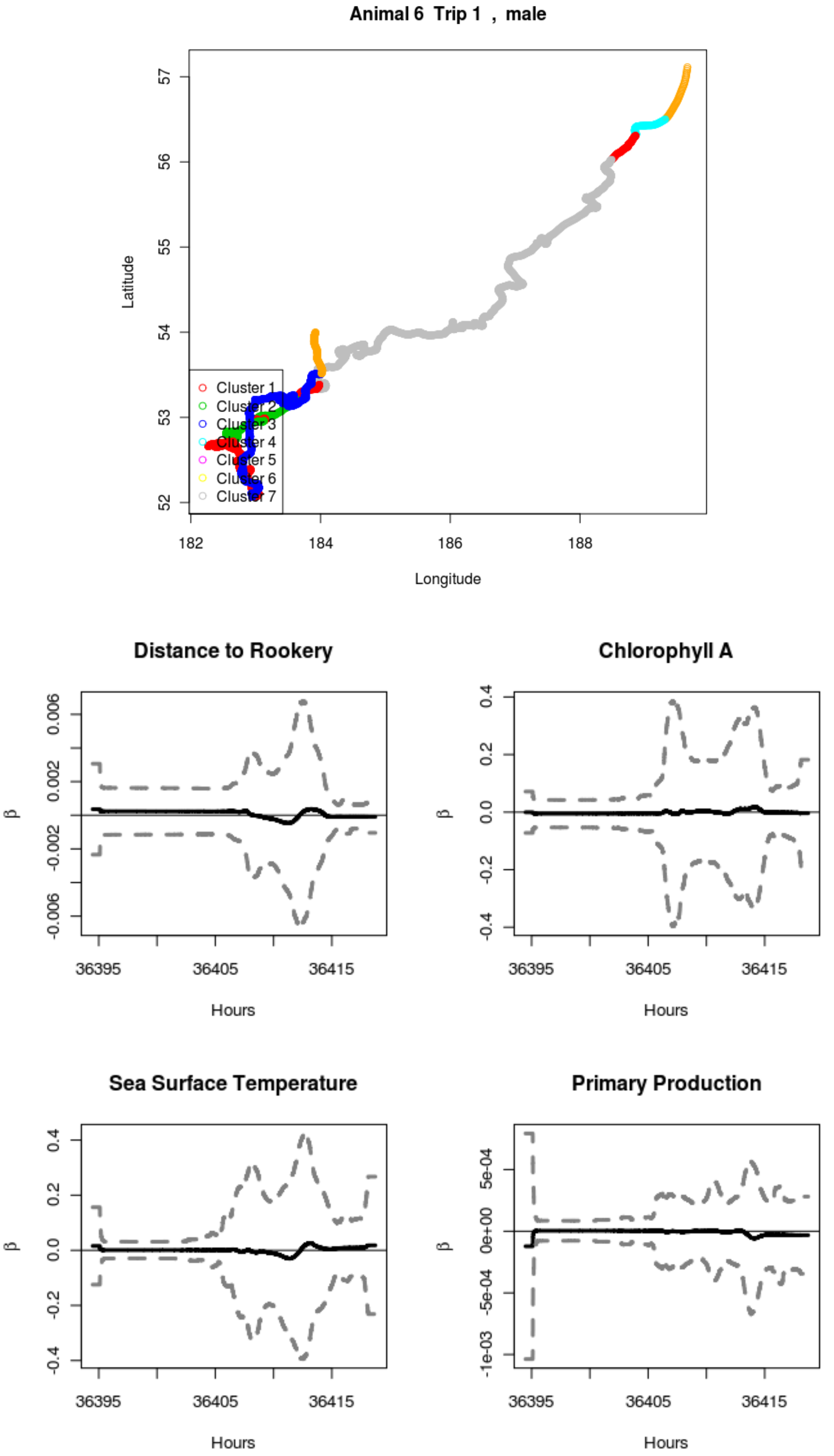
Animal 6 Trip 1 , male
Latitude
Longitude
Cluster 1
Cluster 2
Cluster 3
Cluster 4
Cluster 5
Cluster 6
Cluster 7
52
53
54
55
56
57
182
184
186
188
Distance to Rookery
Chlorophyll A
Sea Surface Temperature
Primary Production
β
Hours
36395
36405
36415
-0.006
-0.002
0.002
0.006
-0.4
-0.2
0.0
0.2
0.4
-1e-03
-5e-04
0e+00
5e-04

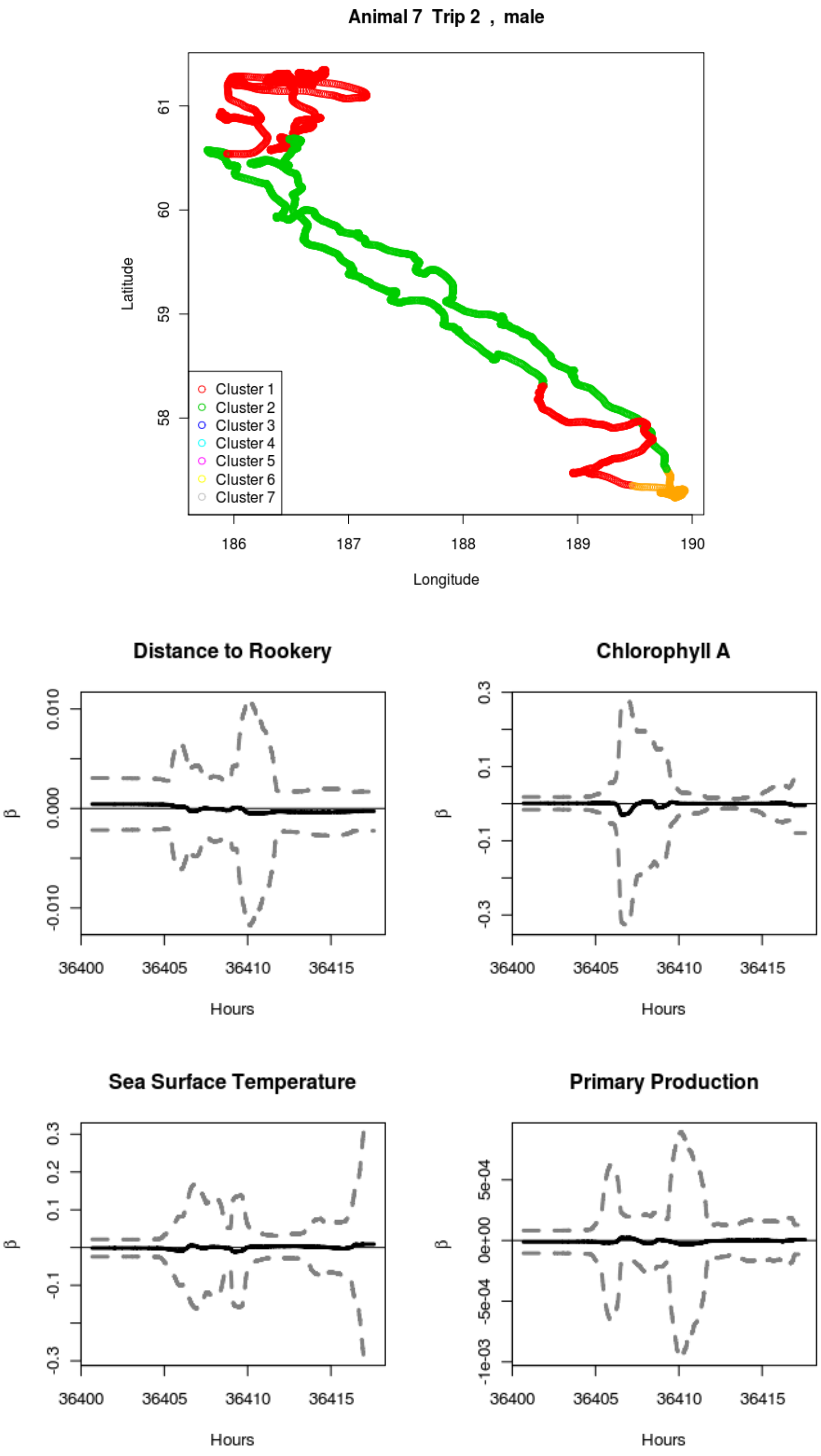
Animal 7 Trip 2 , male
Latitude
Longitude
Cluster 1
Cluster 2
Cluster 3
Cluster 4
Cluster 5
Cluster 6
Cluster 7
Distance to Rookery
Chlorophyll A
Sea Surface Temperature
Primary Production
β
Hours

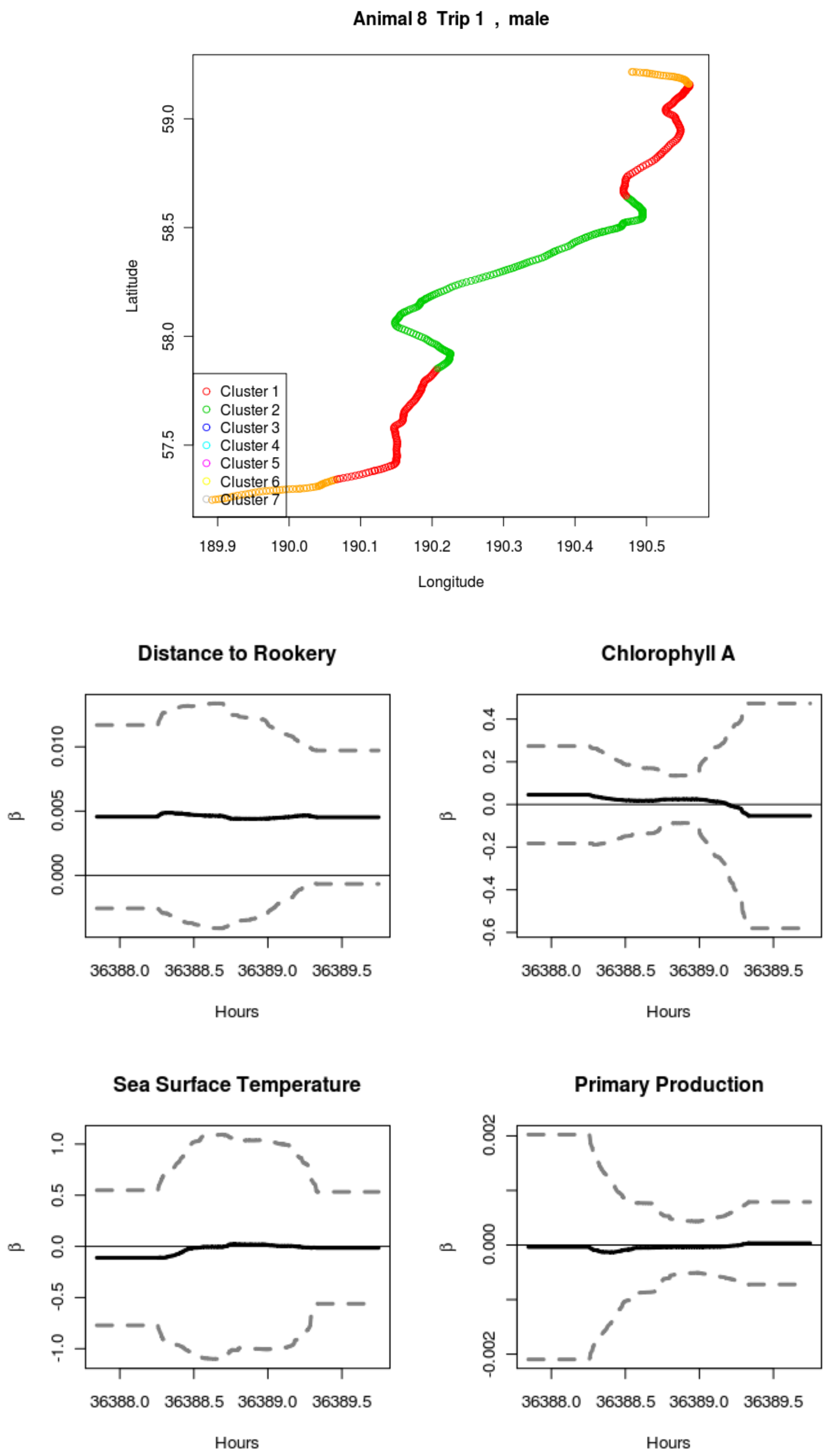
Animal 8 Trip 1 , male
Latitude
Longitude
Cluster 1
Cluster 2
Cluster 3
Cluster 4
Cluster 5
Cluster 6
Cluster 7
Distance to Rookery
Chlorophyll A
Sea Surface Temperature
Primary Production
β
Hours

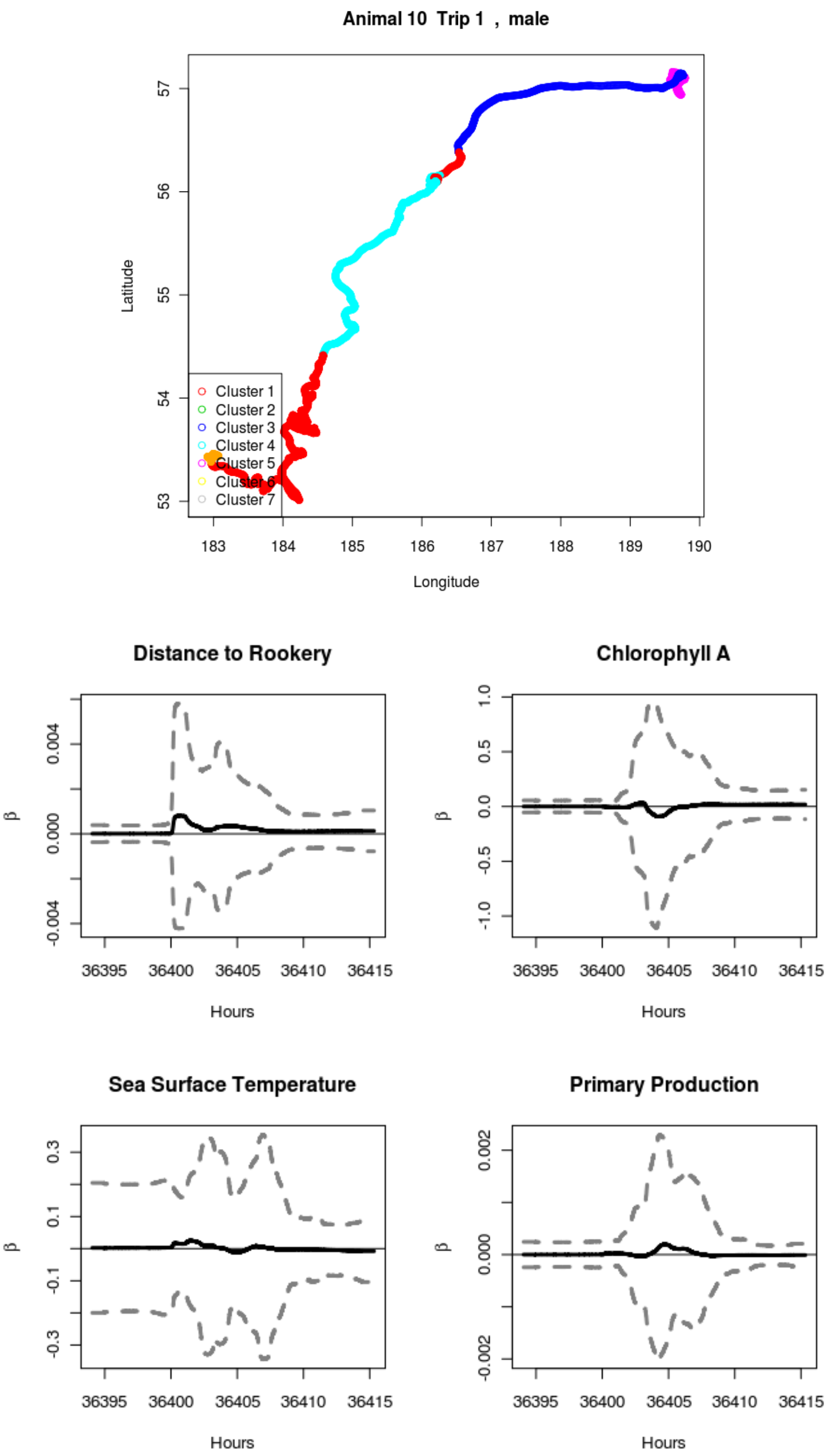
Animal 10 Trip 1 , male
Latitude
Longitude
Cluster 1
Cluster 2
Cluster 3
Cluster 4
Cluster 5
Cluster 6
Cluster 7
Distance to Rookery
Chlorophyll A
Sea Surface Temperature
Primary Production
β
Hours

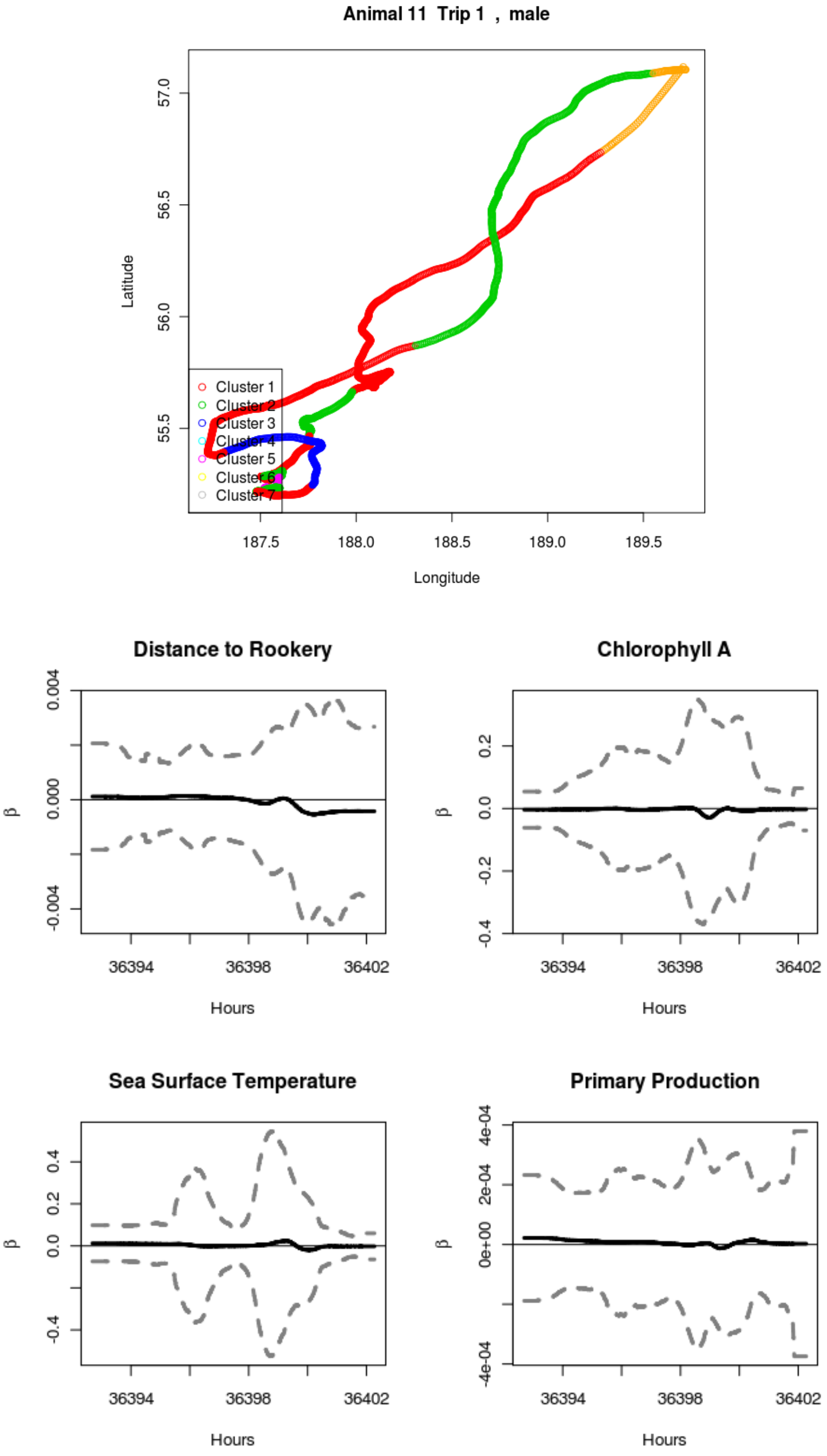

Animal 11 Trip 1 , male
Latitude
Longitude
57.0
56.5
56.0
55.5
187.5
188.0
188.5
189.0
189.5
Cluster 1
Cluster 2
Cluster 3
Cluster 4
Cluster 5
Cluster 6
Cluster 7
Distance to Rookery
Chlorophyll A
Sea Surface Temperature
Primary Production
β
Hours
36394
36398
36402

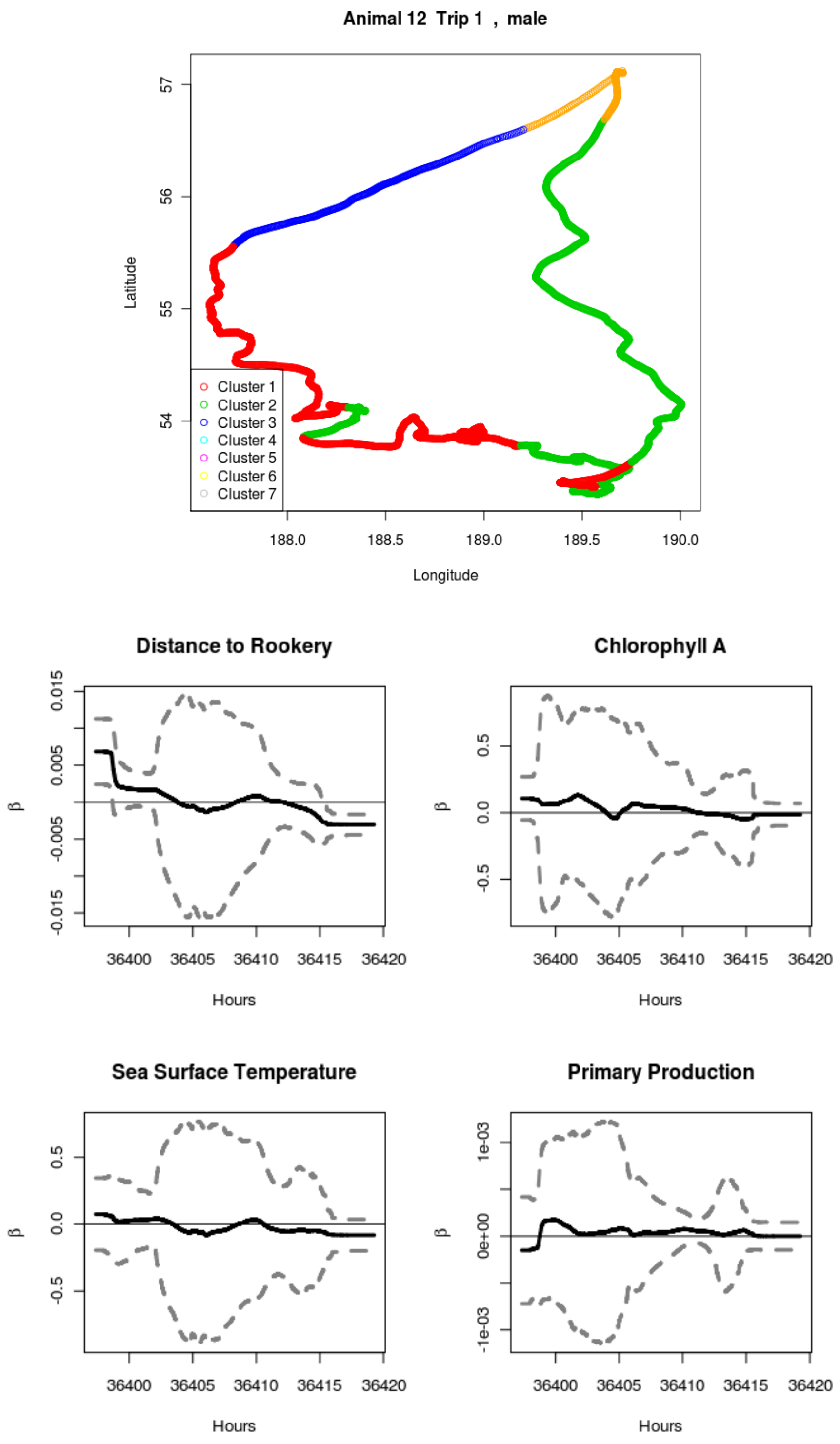
Animal 12 Trip 1 , male
Latitude
57
56
55
54
Cluster 1
Cluster 2
Cluster 3
Cluster 4
Cluster 5
Cluster 6
Cluster 7
188.0
188.5
189.0
189.5
190.0
Longitude
Distance to Rookery
β
0.015
0.005
-0.005
-0.015
36400
36405
36410
36415
36420
Hours
Chlorophyll A
β
0.5
0.0
-0.5
36400
36405
36410
36415
36420
Hours
Sea Surface Temperature
β
0.5
0.0
-0.5
36400
36405
36410
36415
36420
Hours
Primary Production
β
1e-03
0e+00
-1e-03
36400
36405
36410
36415
36420
Hours

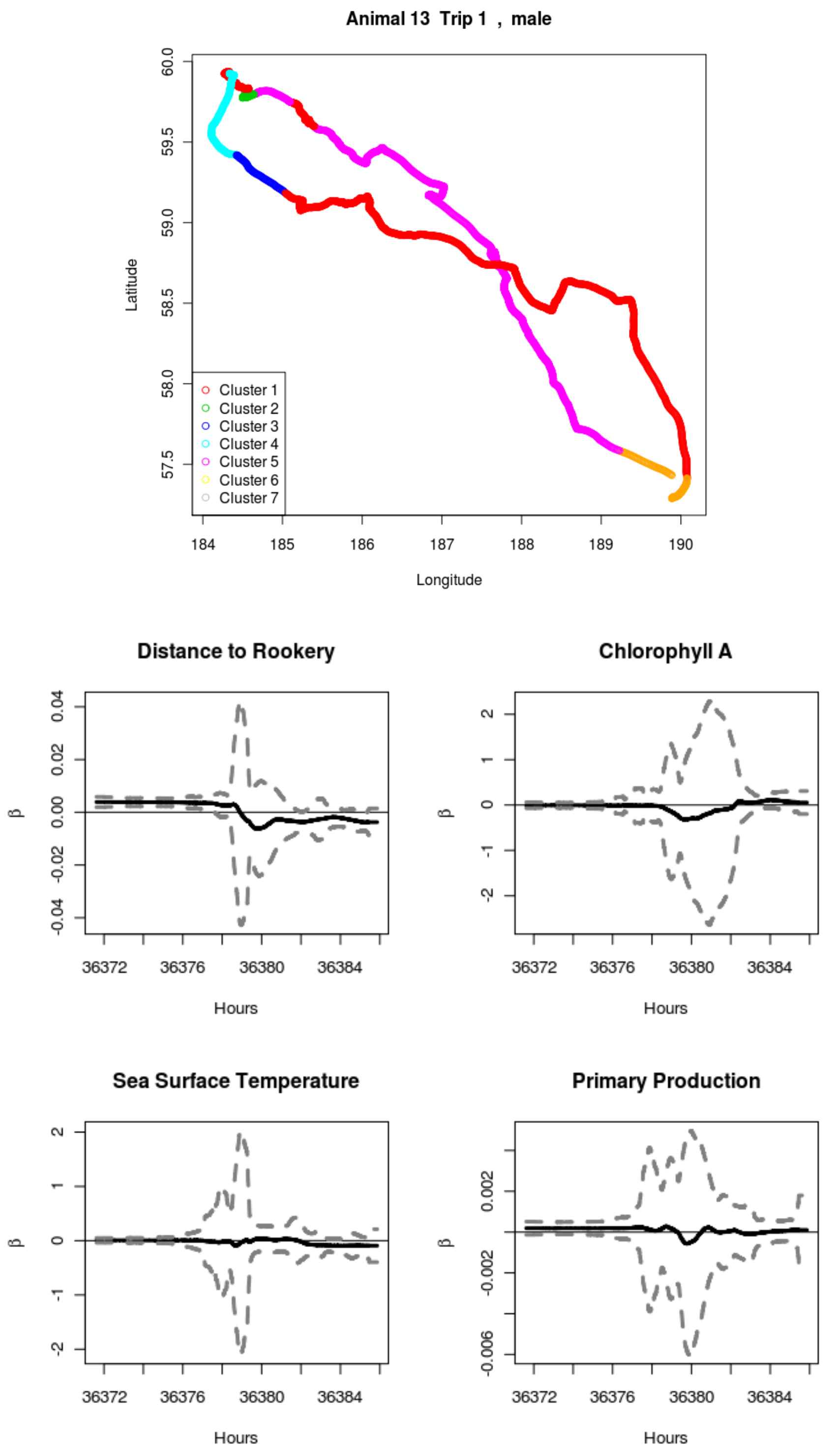
Animal 13 Trip 1 , male
Latitude
Longitude
Cluster 1
Cluster 2
Cluster 3
Cluster 4
Cluster 5
Cluster 6
Cluster 7
Distance to Rookery
Chlorophyll A
Sea Surface Temperature
Primary Production
β
Hours

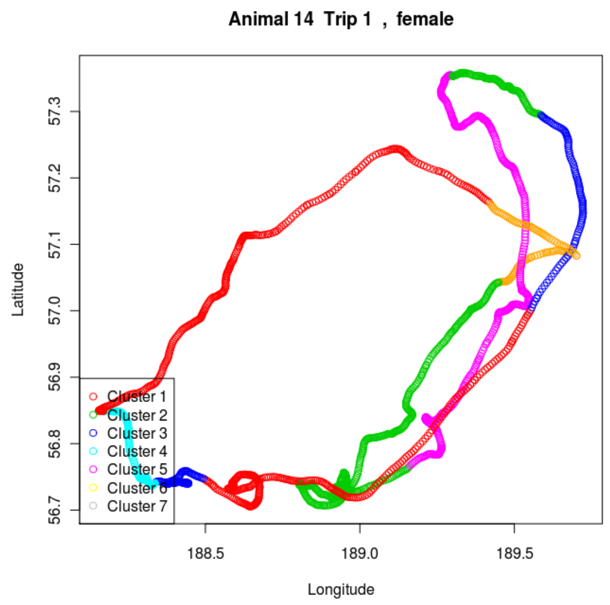
Animal 14 Trip 1 , female
Latitude
57.3
57.2
57.1
57.0
56.9
56.8
56.7
188.5
189.0
189.5
Longitude
Cluster 1
Cluster 2
Cluster 3
Cluster 4
Cluster 5
Cluster 6
Cluster 7

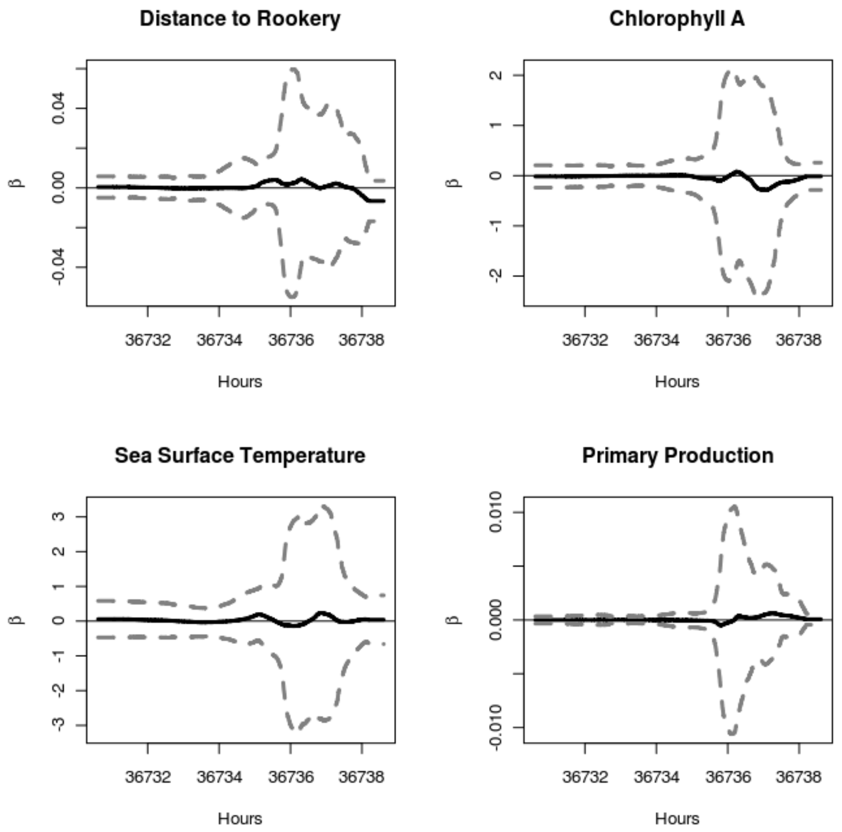
Distance to Rookery
β
0.04
0.00
-0.04
36732
36734
36736
36738
Hours
Chlorophyll A
β
2
1
0
-1
-2
36732
36734
36736
36738
Hours
Sea Surface Temperature
β
3
2
1
0
-1
-2
-3
36732
36734
36736
36738
Hours
Primary Production
β
0.010
0.000
-0.010
36732
36734
36736
36738
Hours

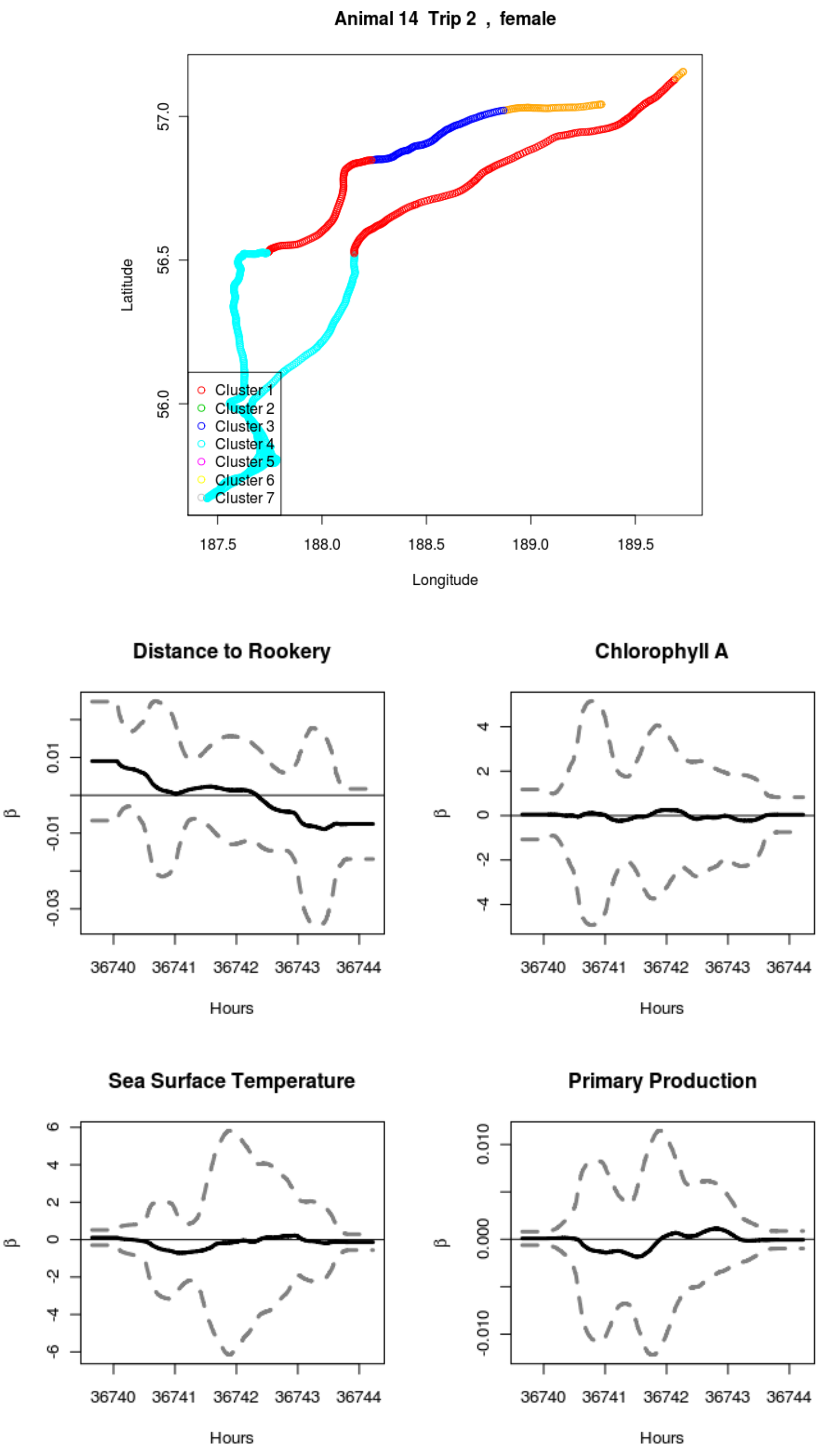

Animal 14 Trip 2 , female
Latitude
Longitude
Cluster 1
Cluster 2
Cluster 3
Cluster 4
Cluster 5
Cluster 6
Cluster 7
Distance to Rookery
Chlorophyll A
Sea Surface Temperature
Primary Production
β
Hours

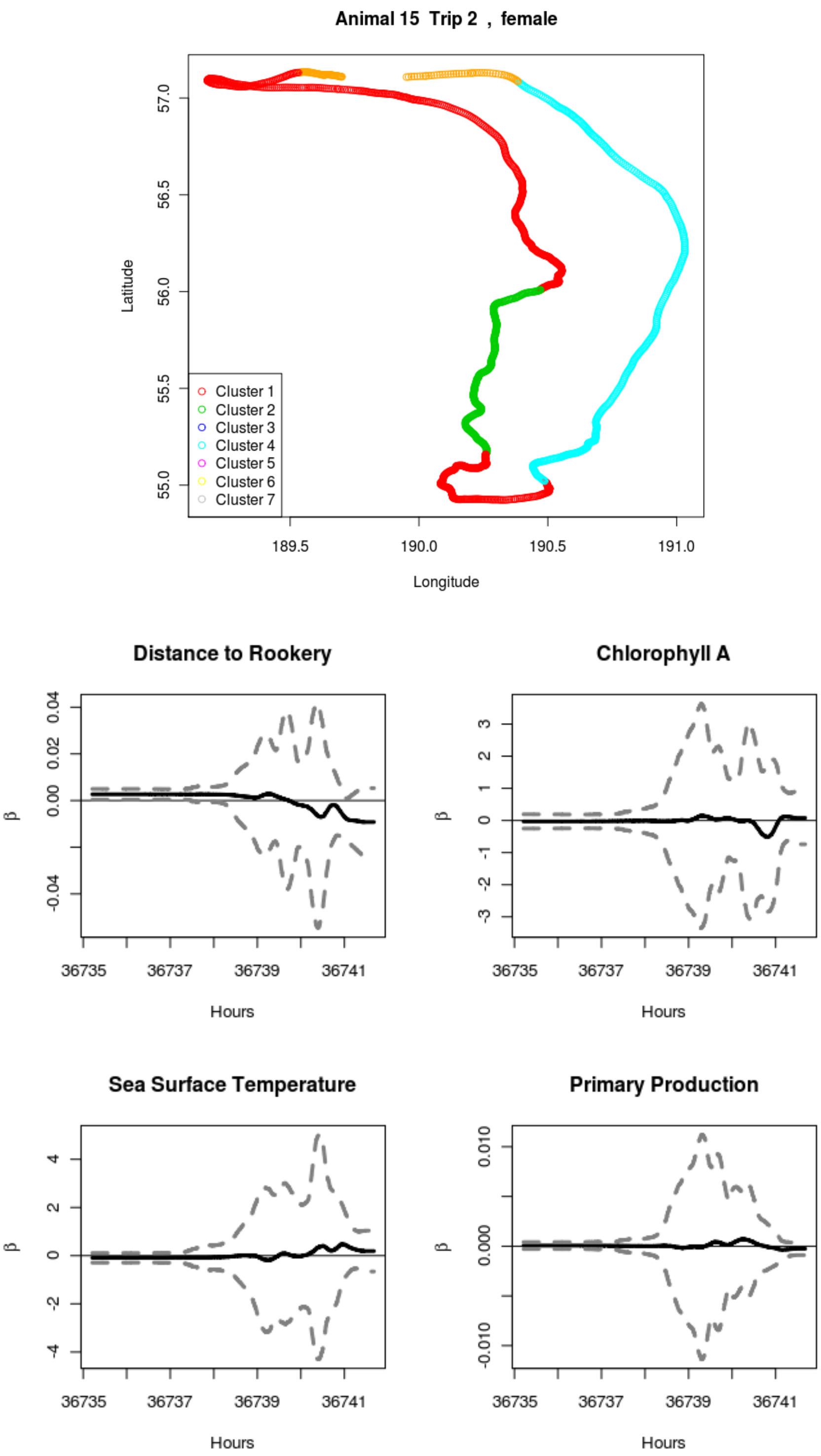
Animal 15 Trip 2 , female
Latitude
57.0
56.5
56.0
55.5
55.0
Cluster 1
Cluster 2
Cluster 3
Cluster 4
Cluster 5
Cluster 6
Cluster 7
189.5
190.0
190.5
191.0
Longitude
Distance to Rookery
β
0.04
0.02
0.00
-0.04
36735
36737
36739
36741
Hours
Chlorophyll A
β
3
2
1
0
-1
-2
-3
36735
36737
36739
36741
Hours
Sea Surface Temperature
β
4
2
0
-2
-4
36735
36737
36739
36741
Hours
Primary Production
β
0.010
0.000
-0.010
36735
36737
36739
36741
Hours

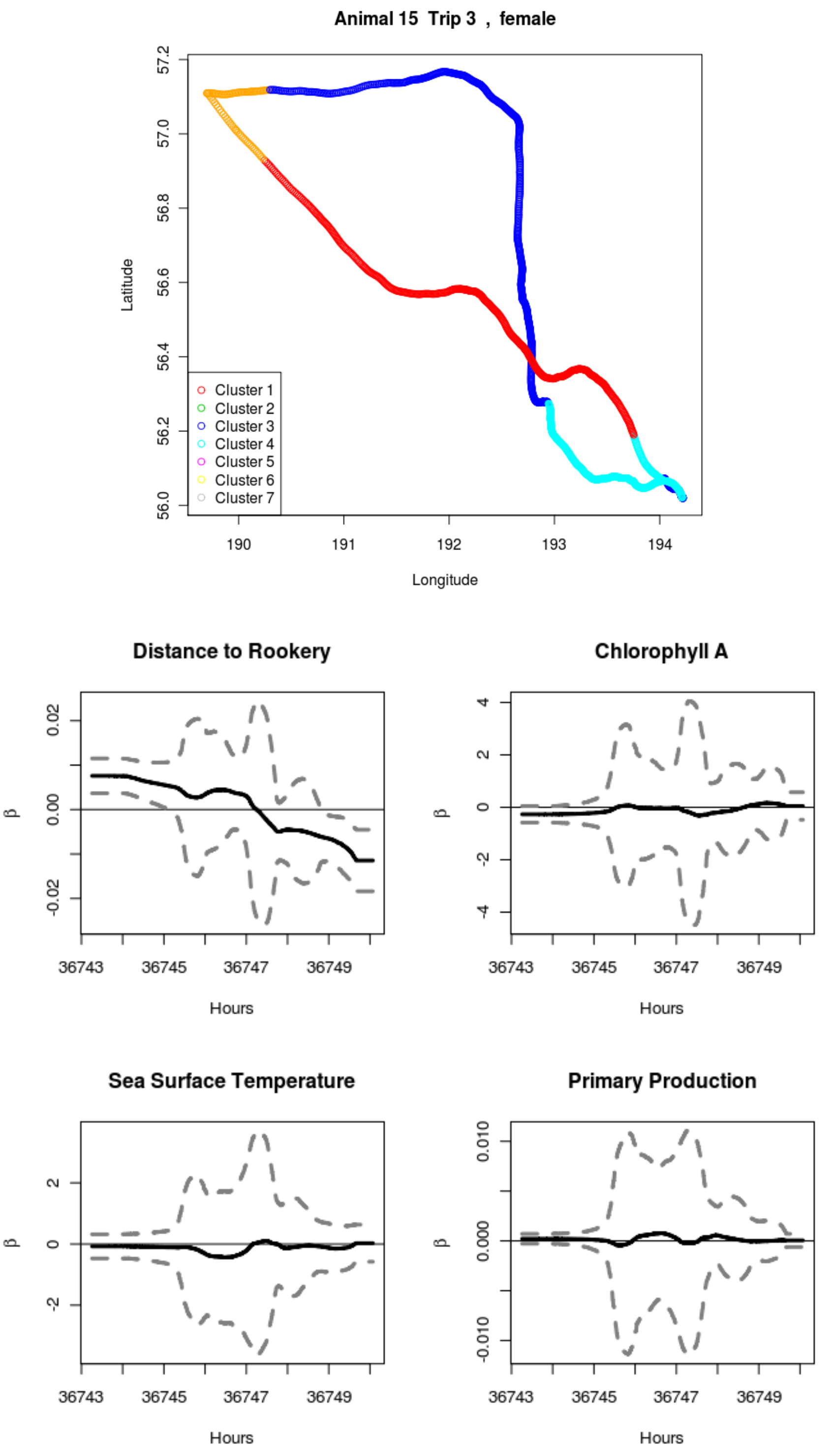
Animal 15 Trip 3 , female
Latitude
Longitude
Cluster 1
Cluster 2
Cluster 3
Cluster 4
Cluster 5
Cluster 6
Cluster 7
Distance to Rookery
Chlorophyll A
Sea Surface Temperature
Primary Production
β
Hours

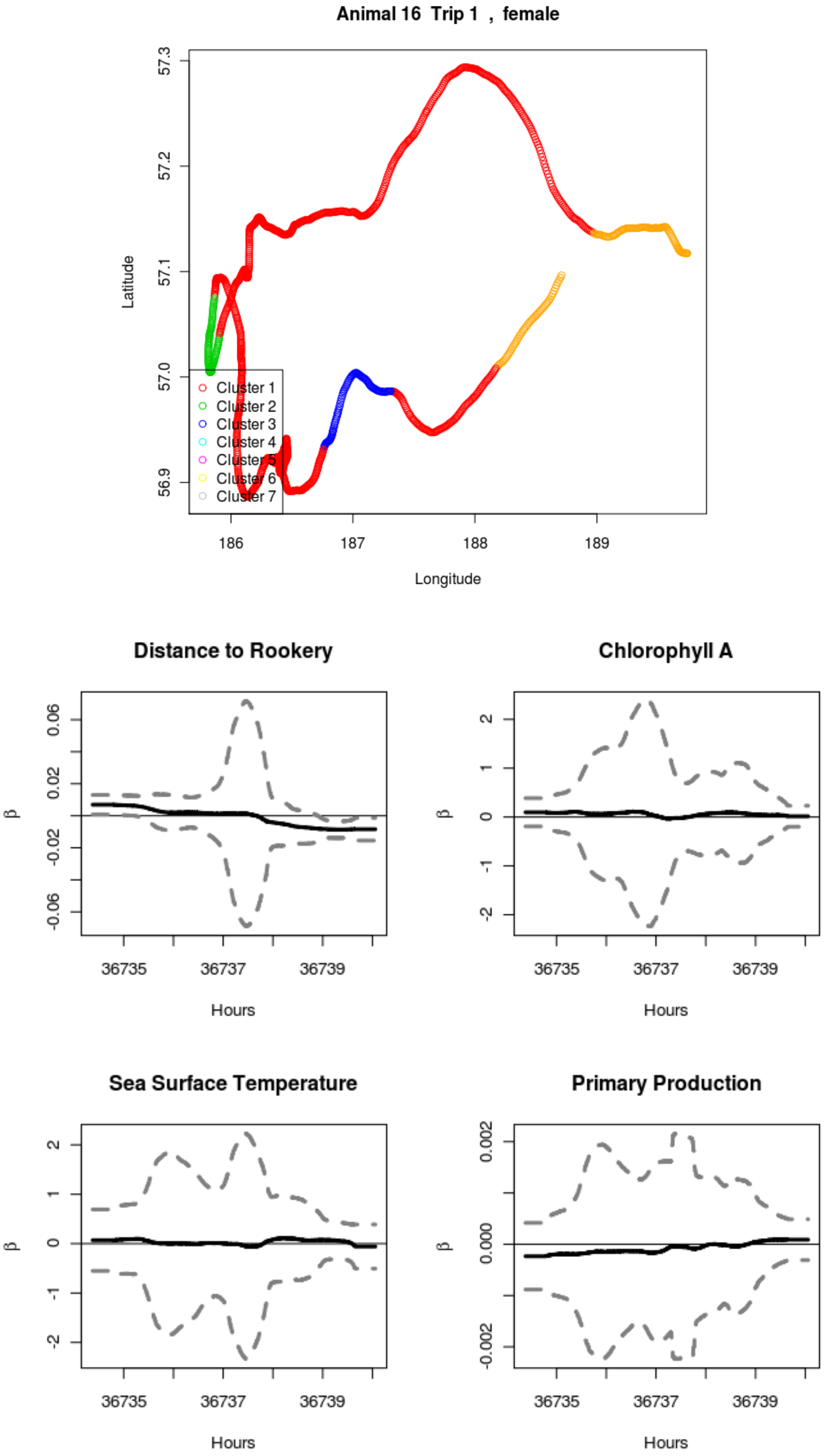
Animal 16 Trip 1 , female
Latitude
Longitude
Cluster 1
Cluster 2
Cluster 3
Cluster 4
Cluster 5
Cluster 6
Cluster 7
Distance to Rookery
Chlorophyll A
Sea Surface Temperature
Primary Production
β
Hours

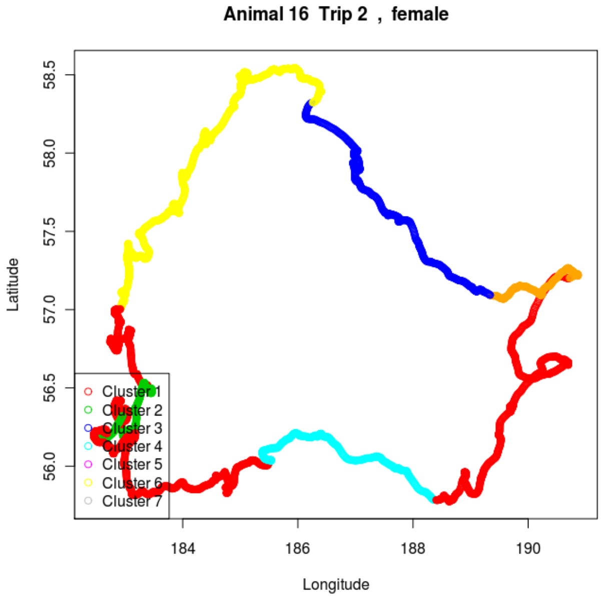
Animal 16 Trip 2 , female
Latitude
Longitude
Cluster 1
Cluster 2
Cluster 3
Cluster 4
Cluster 5
Cluster 6
Cluster 7
56.0
56.5
57.0
57.5
58.0
58.5
184
186
188
190

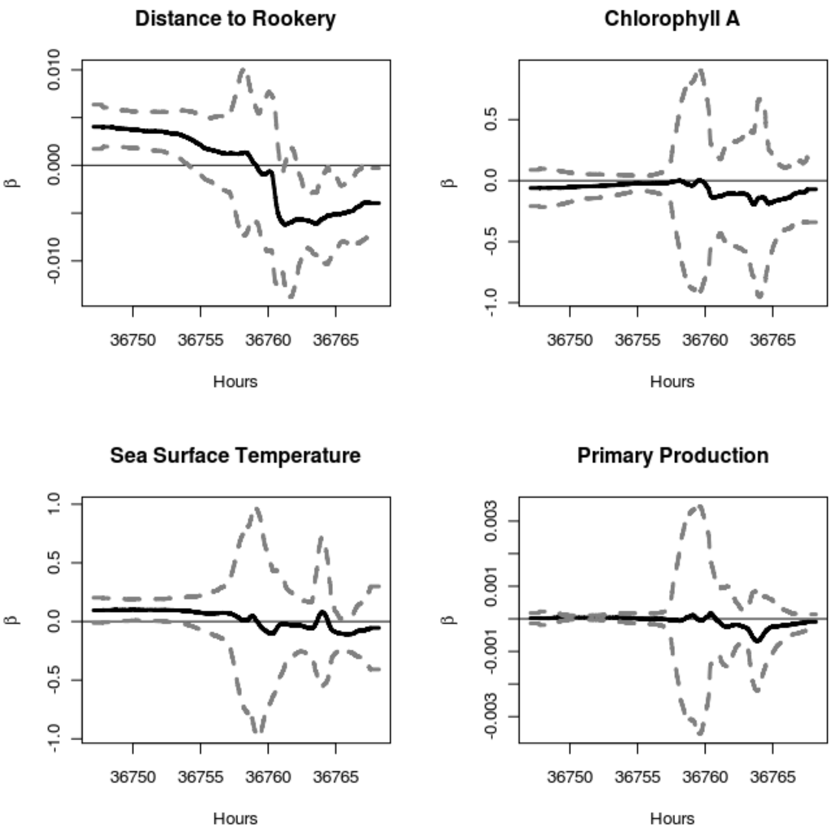
Distance to Rookery
Chlorophyll A
Sea Surface Temperature
Primary Production
β
Hours
36750
36755
36760
36765

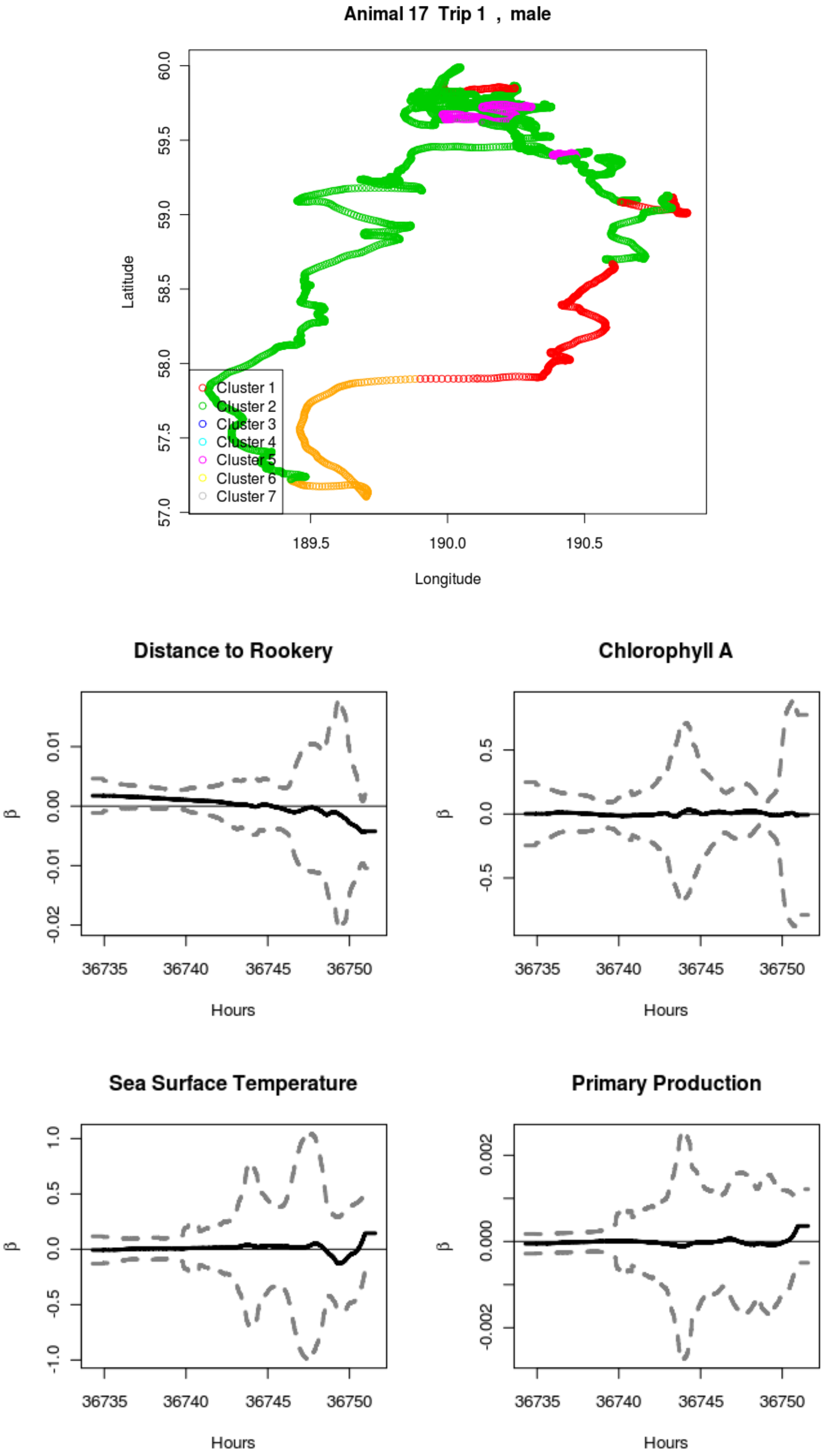
Animal 17 Trip 1 , male
Latitude
Longitude
Cluster 1
Cluster 2
Cluster 3
Cluster 4
Cluster 5
Cluster 6
Cluster 7
Distance to Rookery
Chlorophyll A
Sea Surface Temperature
Primary Production
β
Hours

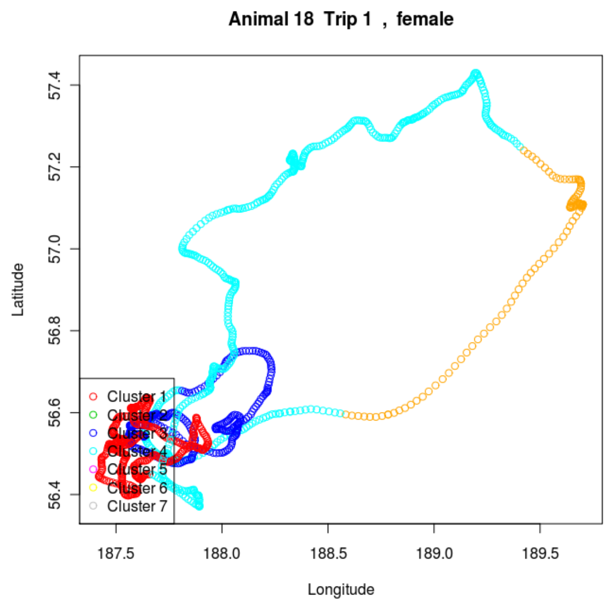
Animal 18 Trip 1 , female
Latitude
Longitude
Cluster 1
Cluster 2
Cluster 3
Cluster 4
Cluster 5
Cluster 6
Cluster 7
56.4
56.6
56.8
57.0
57.2
57.4
187.5
188.0
188.5
189.0
189.5

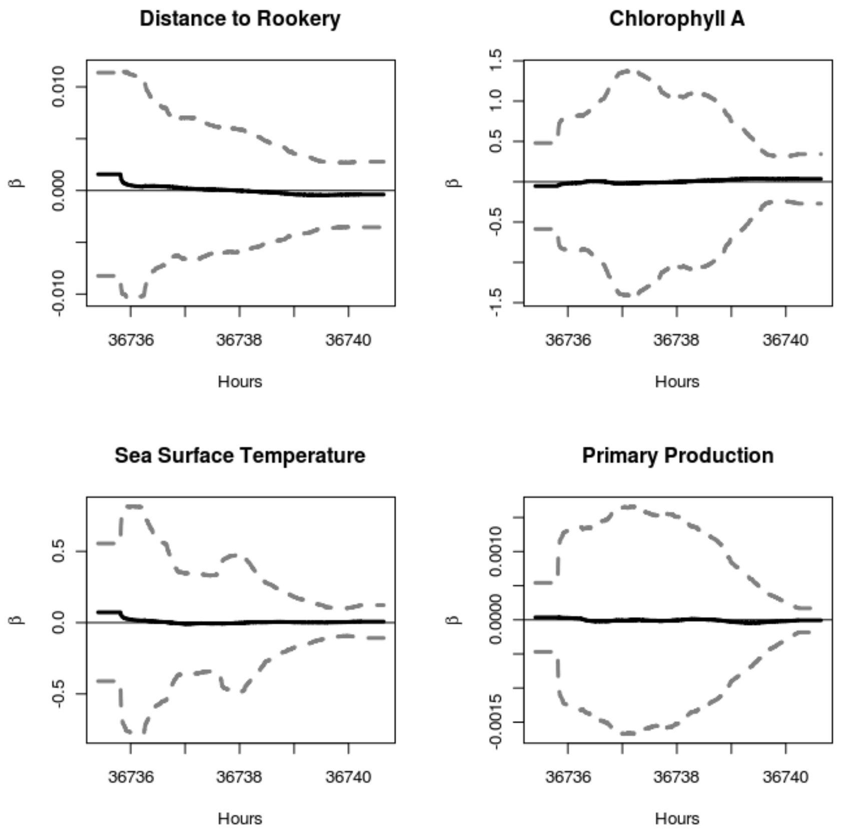
Distance to Rookery
Chlorophyll A
Sea Surface Temperature
Primary Production
β
Hours
36736
36738
36740

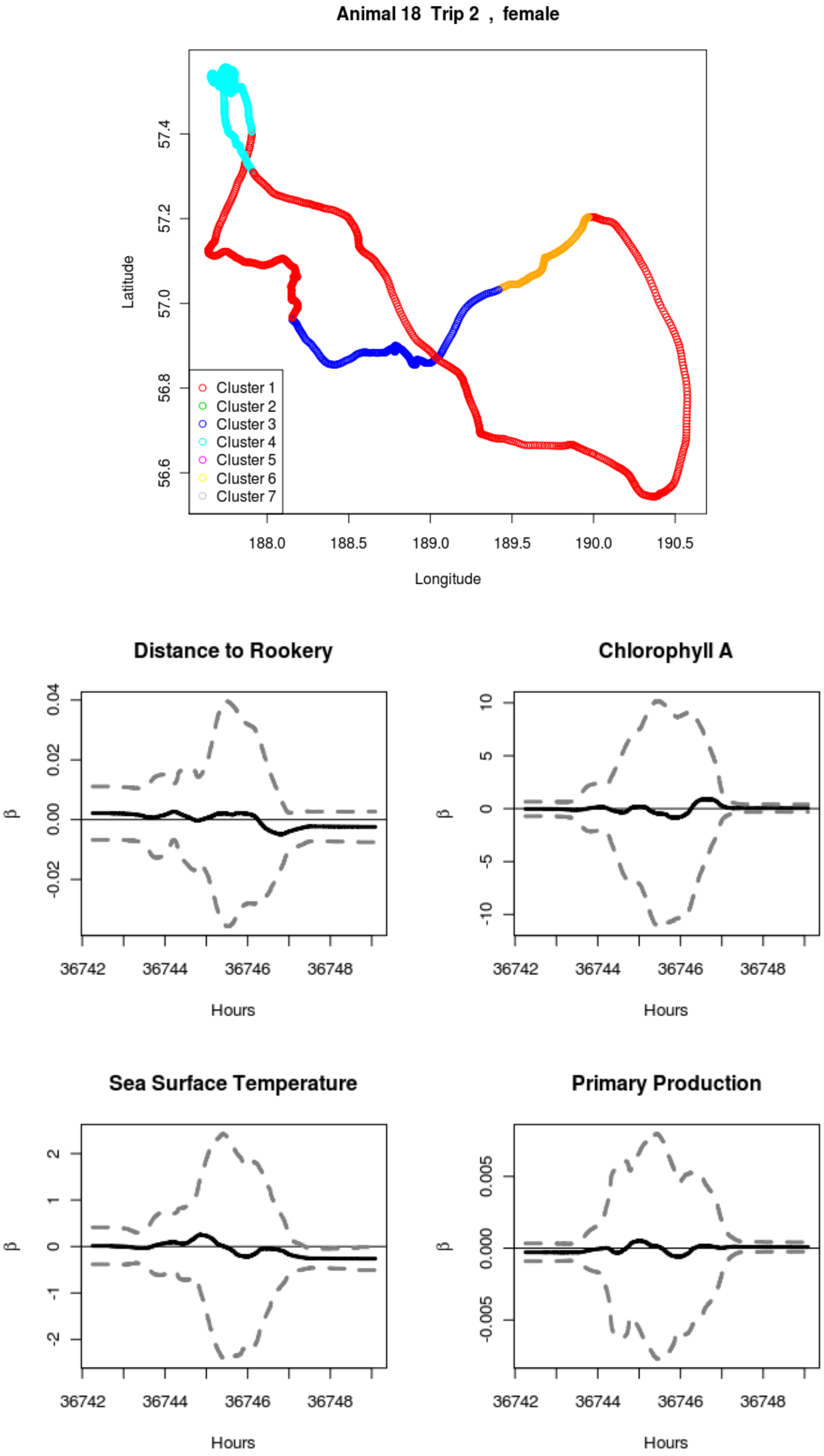
Animal 18 Trip 2 , female
Latitude
Longitude
Cluster 1
Cluster 2
Cluster 3
Cluster 4
Cluster 5
Cluster 6
Cluster 7
Distance to Rookery
Chlorophyll A
Sea Surface Temperature
Primary Production
β
Hours

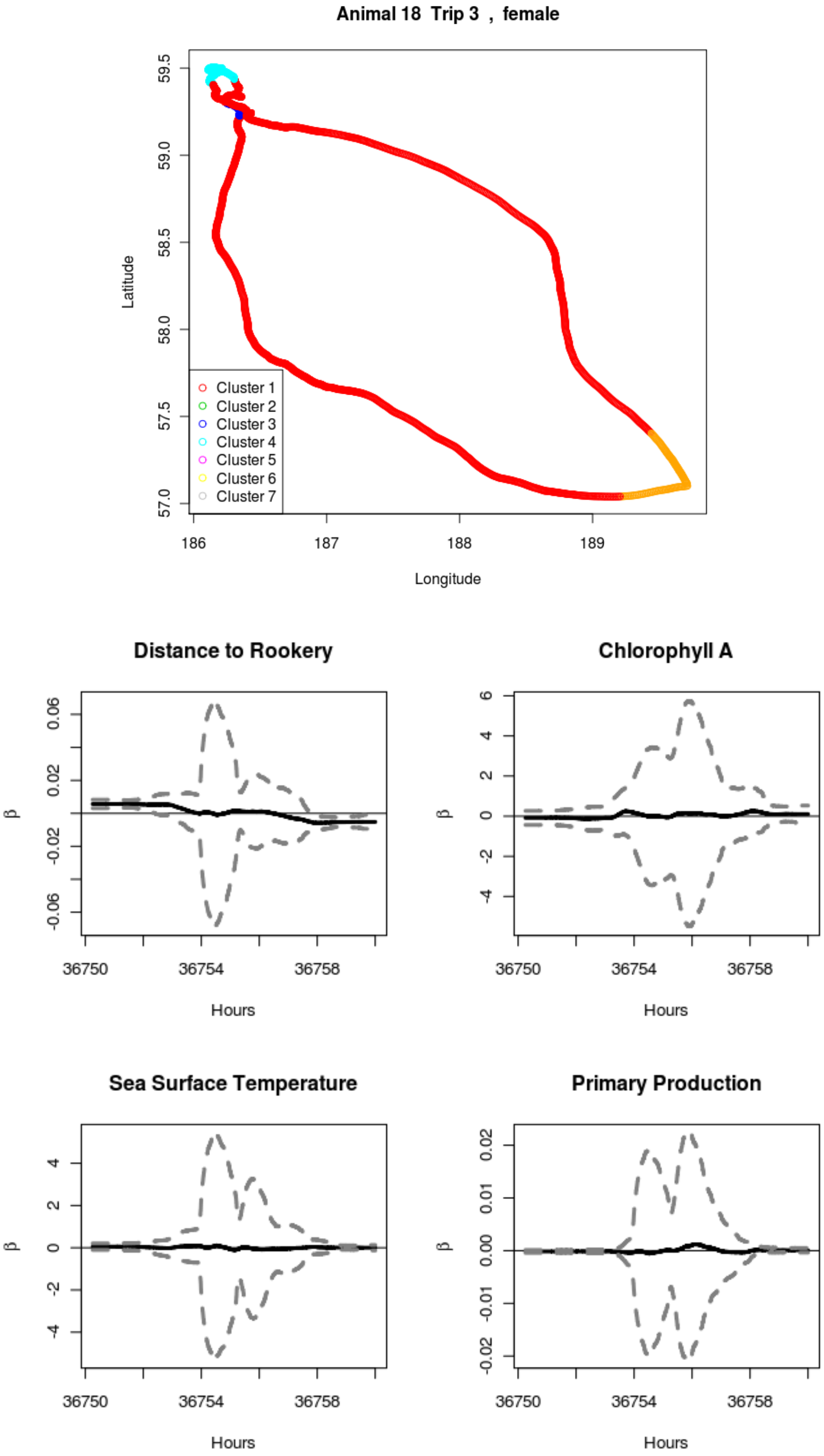
Animal 18 Trip 3 , female
Latitude
Longitude
Cluster 1
Cluster 2
Cluster 3
Cluster 4
Cluster 5
Cluster 6
Cluster 7
Distance to Rookery
Chlorophyll A
Sea Surface Temperature
Primary Production
β
Hours

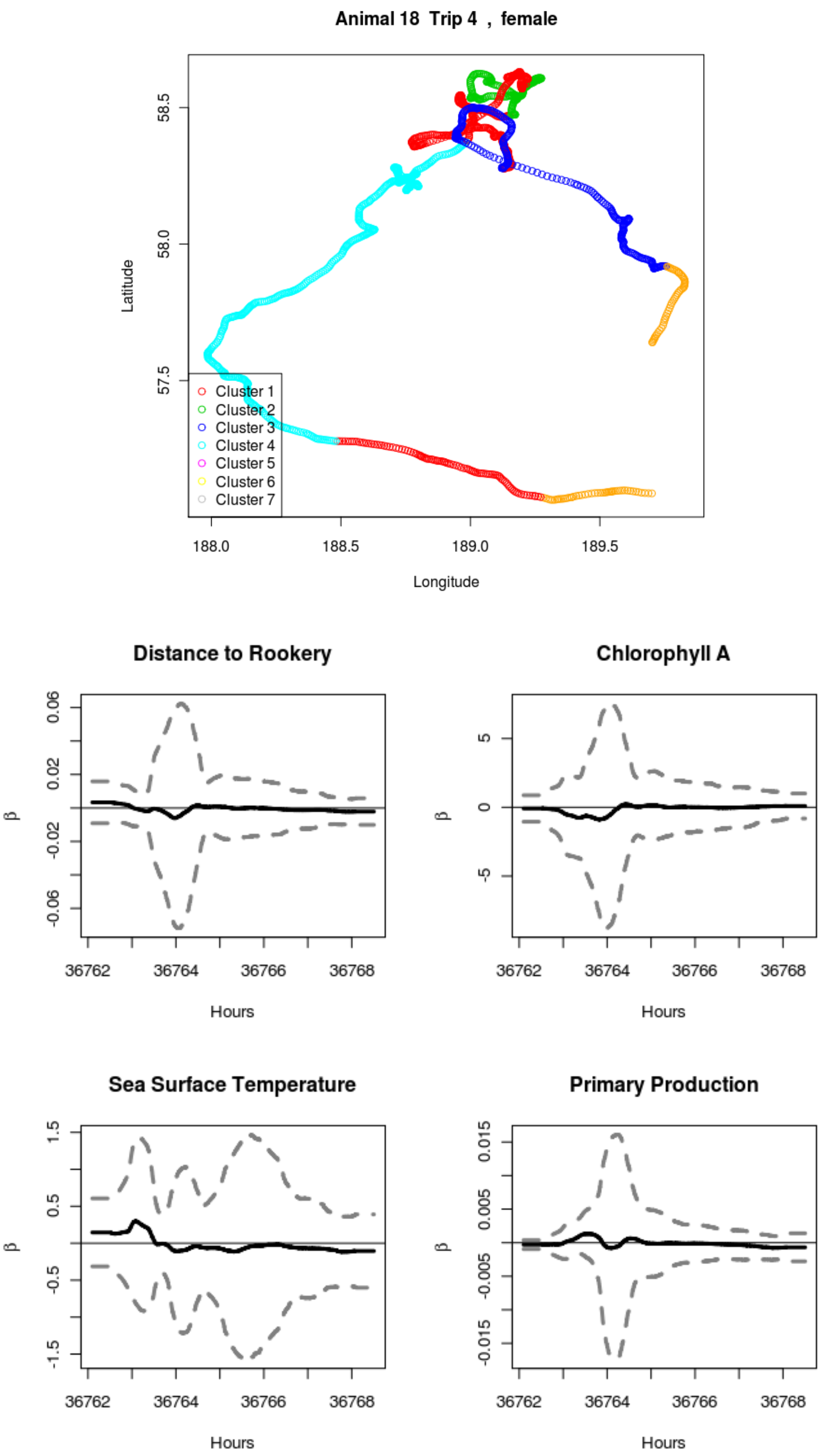
Animal 18 Trip 4 , female
Latitude
58.5
58.0
57.5
Longitude
188.0
188.5
189.0
189.5
Cluster 1
Cluster 2
Cluster 3
Cluster 4
Cluster 5
Cluster 6
Cluster 7
Distance to Rookery
0.06
0.02
-0.02
-0.06
β
36762
36764
36766
36768
Hours
Chlorophyll A
5
0
-5
Sea Surface Temperature
1.5
0.5
-0.5
-1.5
Primary Production
0.015
0.005
-0.005
-0.015

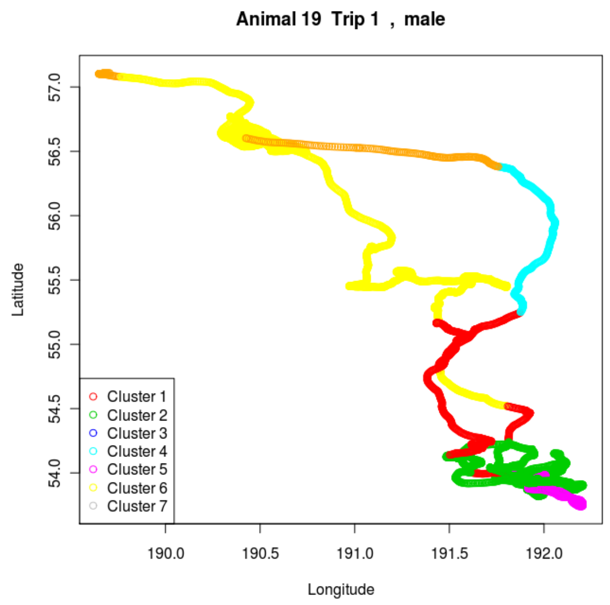
Animal 19 Trip 1 , male
Latitude
57.0
56.5
56.0
55.5
55.0
54.5
54.0
Longitude
190.0
190.5
191.0
191.5
192.0
Cluster 1
Cluster 2
Cluster 3
Cluster 4
Cluster 5
Cluster 6
Cluster 7

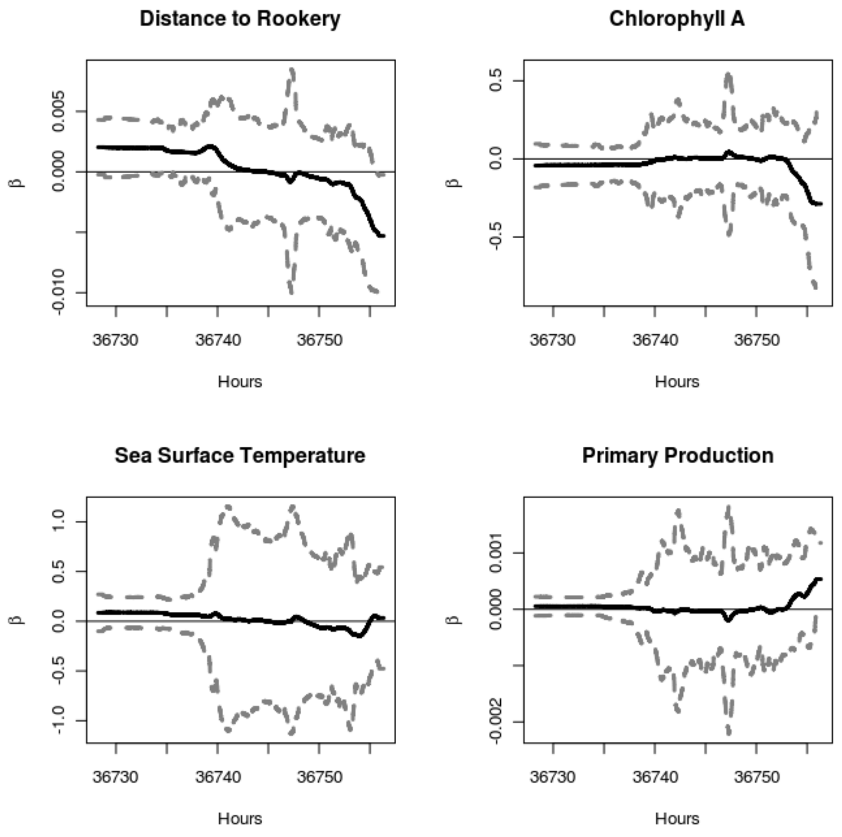
Distance to Rookery
Chlorophyll A
Sea Surface Temperature
Primary Production
β
Hours
36730
36740
36750
0.005
0.000
-0.010
0.5
0.0
-0.5
1.0
-1.0
0.001
-0.002

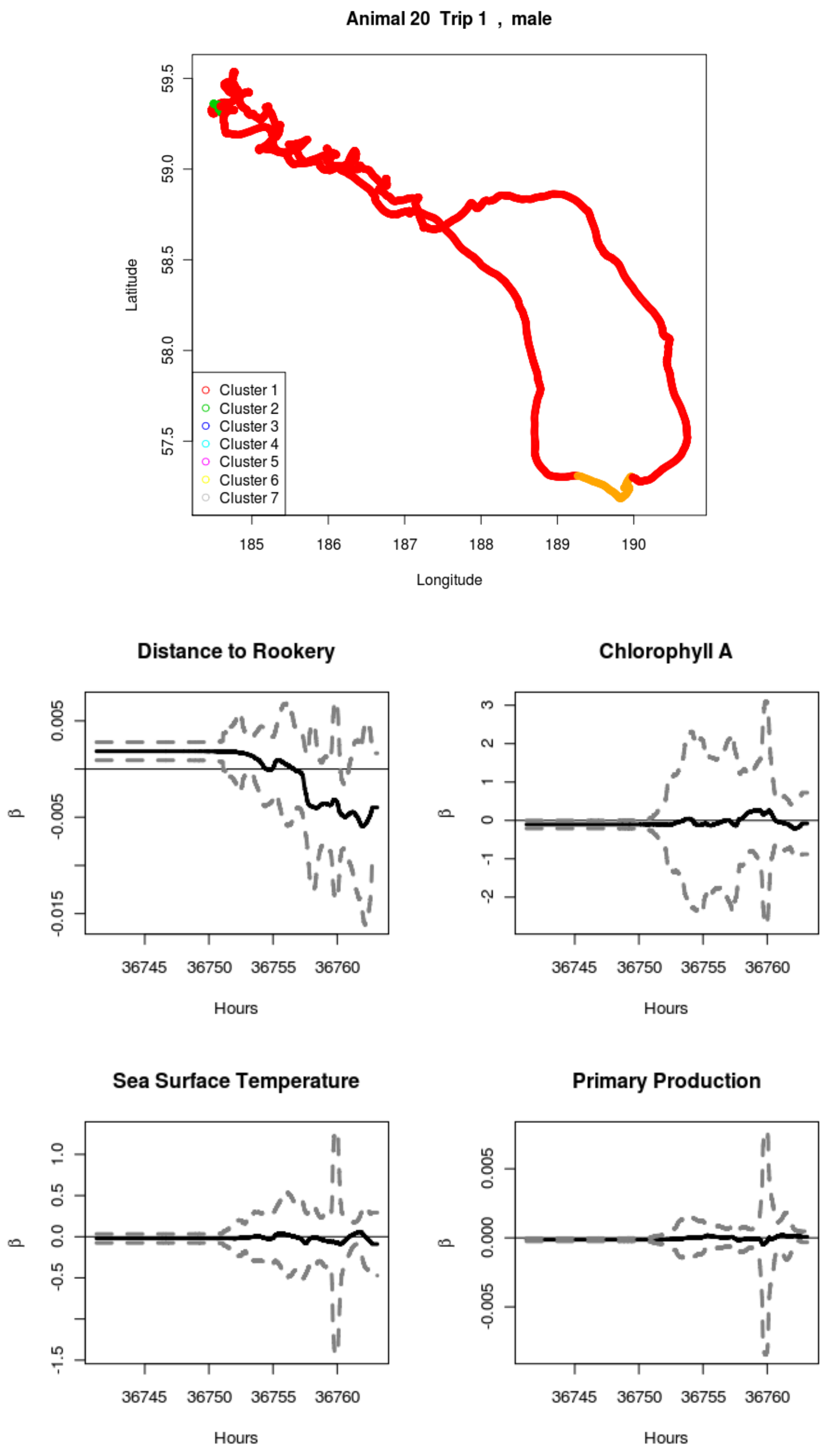
Animal 20 Trip 1 , male
Latitude
Longitude
Cluster 1
Cluster 2
Cluster 3
Cluster 4
Cluster 5
Cluster 6
Cluster 7
Distance to Rookery
Chlorophyll A
Sea Surface Temperature
Primary Production
β
Hours

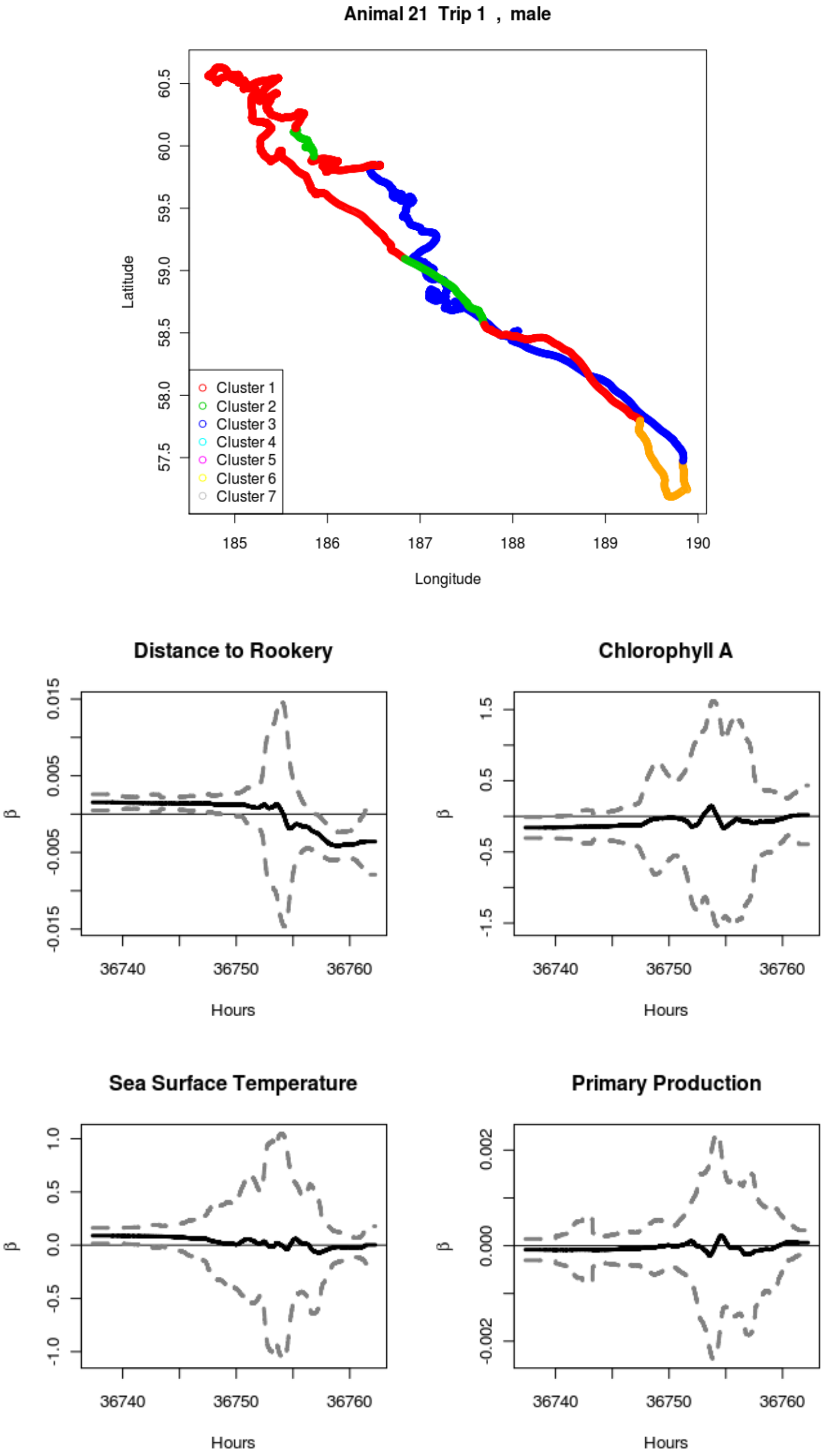
Animal 21 Trip 1 , male
Latitude
Longitude
60.5
60.0
59.5
59.0
58.5
58.0
57.5
185
186
187
188
189
190
Cluster 1
Cluster 2
Cluster 3
Cluster 4
Cluster 5
Cluster 6
Cluster 7
Distance to Rookery
Chlorophyll A
Sea Surface Temperature
Primary Production
β
Hours
36740
36750
36760
0.015
0.005
-0.005
-0.015
1.5
0.5
-0.5
-1.5
1.0
0.5
0.0
-0.5
-1.0
0.002
0.000
-0.002

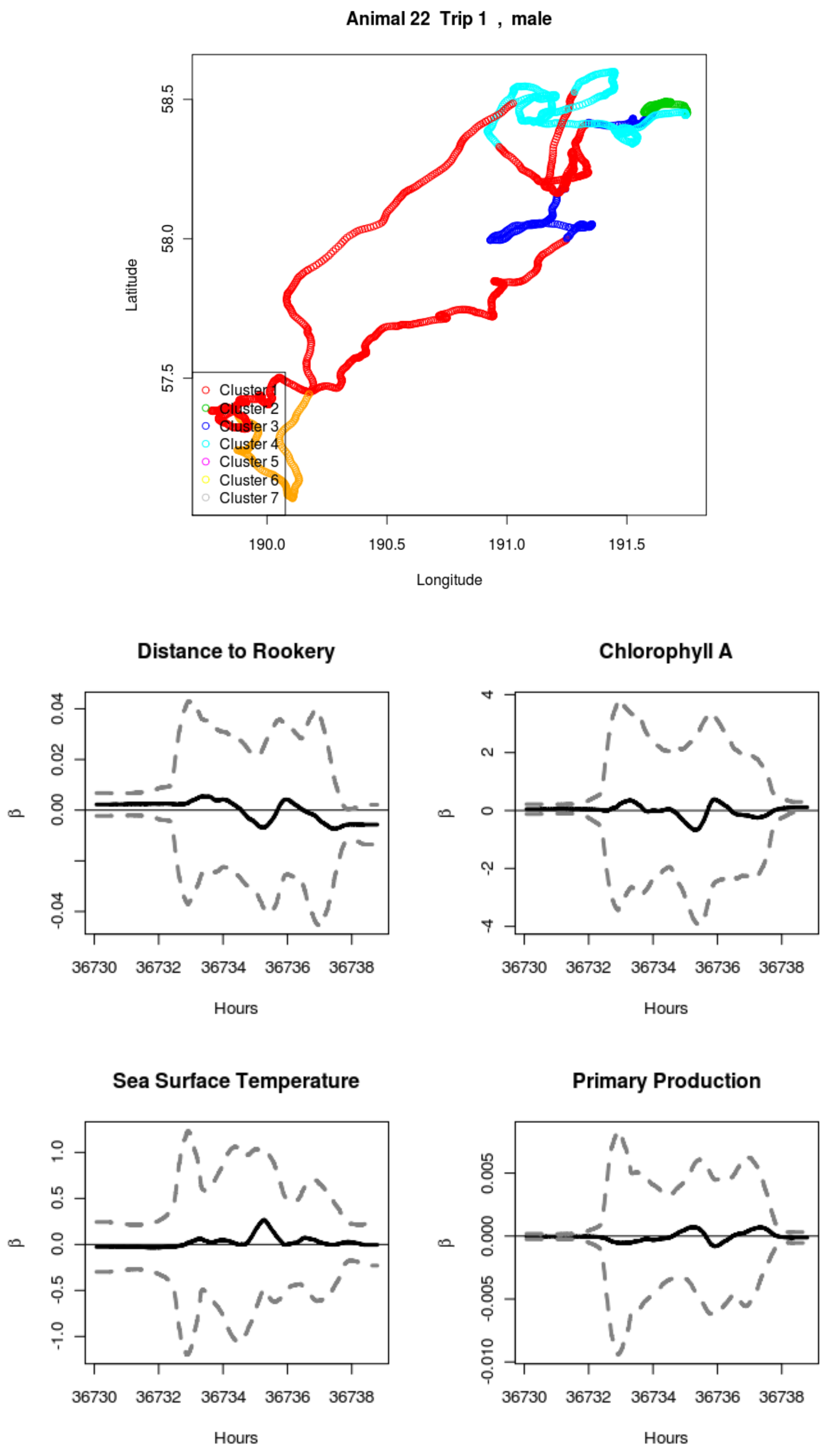
Animal 22 Trip 1 , male
Latitude
Longitude
Cluster 1
Cluster 2
Cluster 3
Cluster 4
Cluster 5
Cluster 6
Cluster 7
Distance to Rookery
Chlorophyll A
Sea Surface Temperature
Primary Production
β
Hours

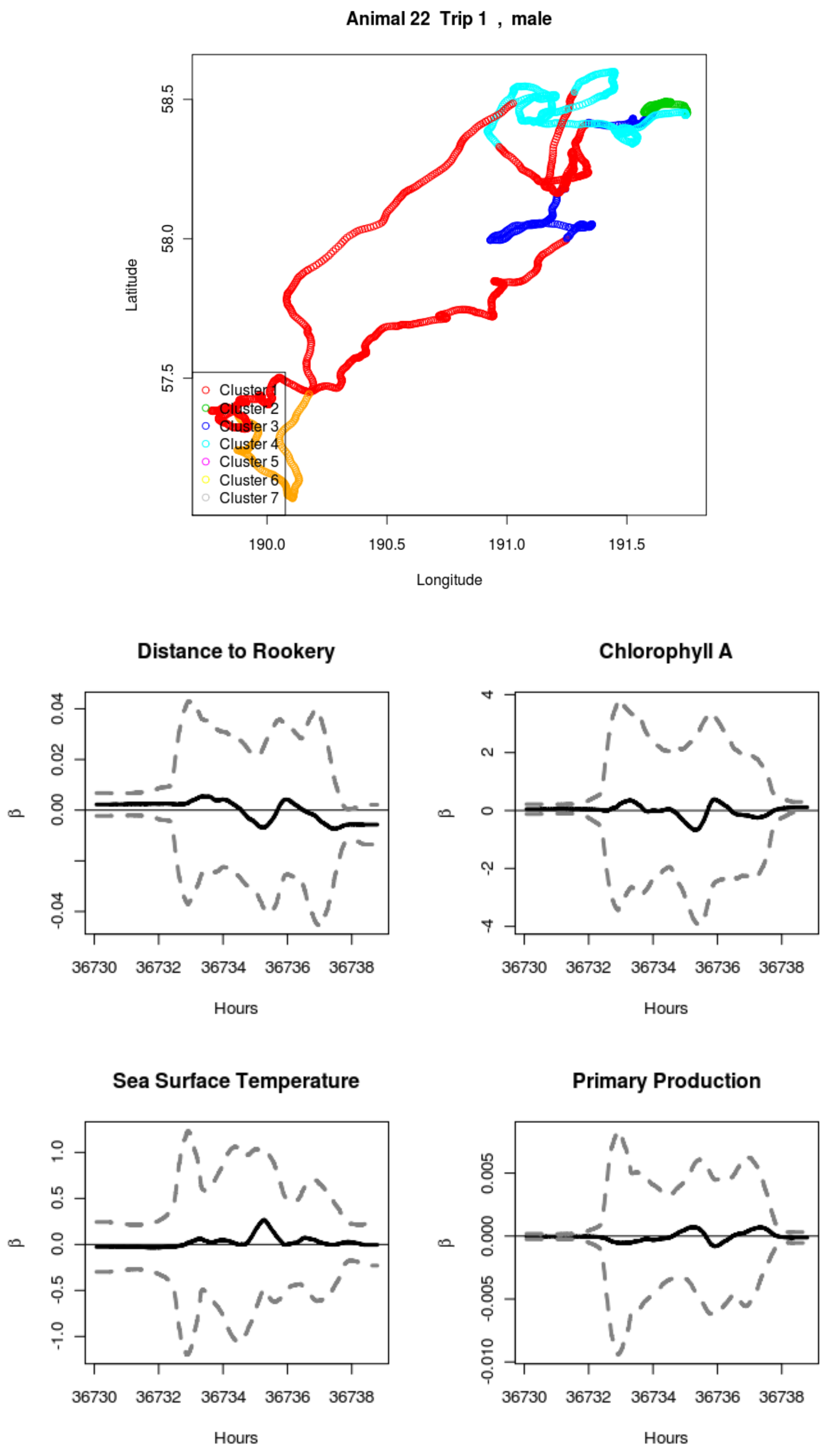

Animal 22 Trip 1 , male
Latitude
Longitude
Cluster 1
Cluster 2
Cluster 3
Cluster 4
Cluster 5
Cluster 6
Cluster 7
Distance to Rookery
Chlorophyll A
Sea Surface Temperature
Primary Production
β
Hours

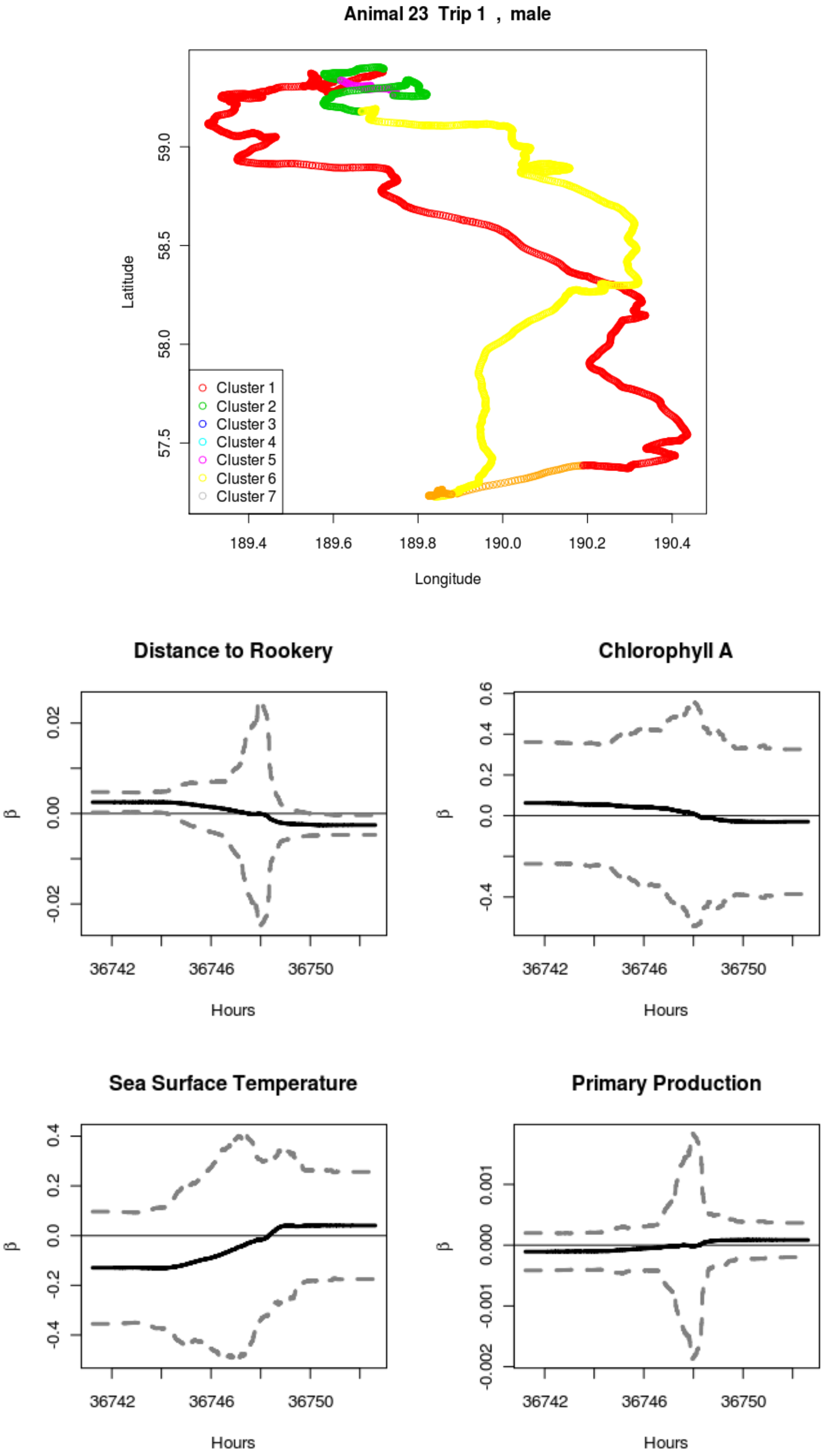
Animal 23 Trip 1 , male
Latitude
Longitude
59.0
58.5
58.0
57.5
189.4
189.6
189.8
190.0
190.2
190.4
Cluster 1
Cluster 2
Cluster 3
Cluster 4
Cluster 5
Cluster 6
Cluster 7
Distance to Rookery
Chlorophyll A
Sea Surface Temperature
Primary Production
β
Hours
36742
36746
36750
0.02
0.00
-0.02
0.6
0.4
0.2
0.0
-0.4
0.2
-0.2
0.001
0.000
-0.001
-0.002

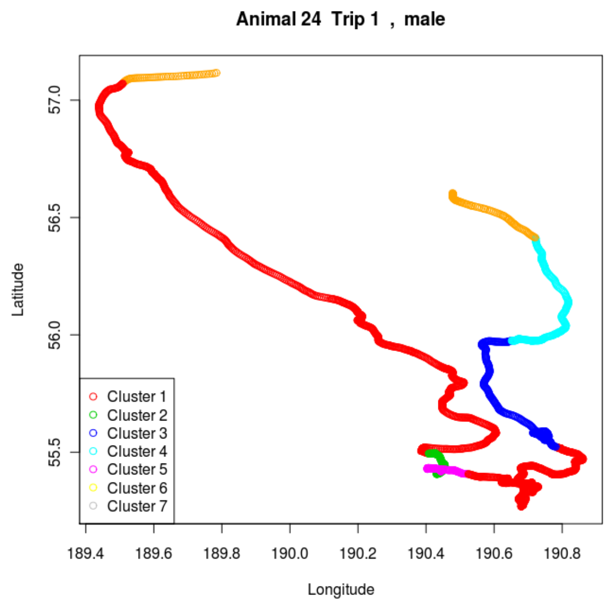
Animal 24 Trip 1 , male
Latitude
Longitude
Cluster 1
Cluster 2
Cluster 3
Cluster 4
Cluster 5
Cluster 6
Cluster 7

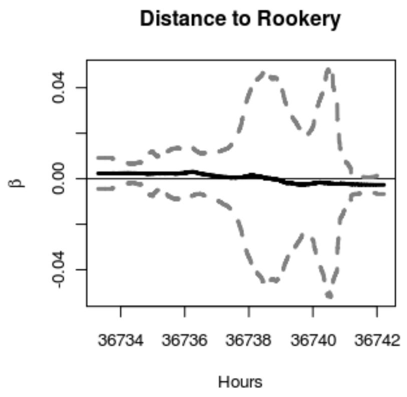
Distance to Rookery
β
Hours

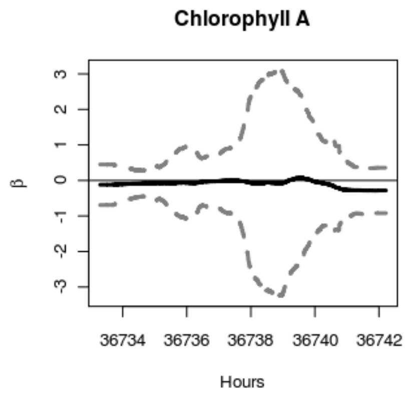
Chlorophyll A
β
Hours

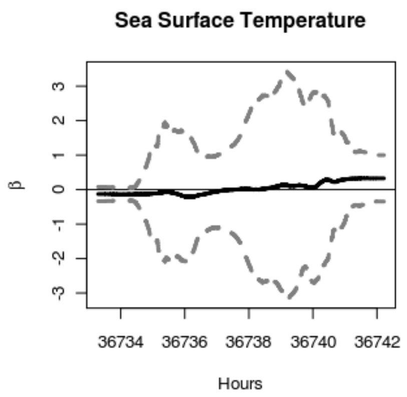
Sea Surface Temperature
β
Hours

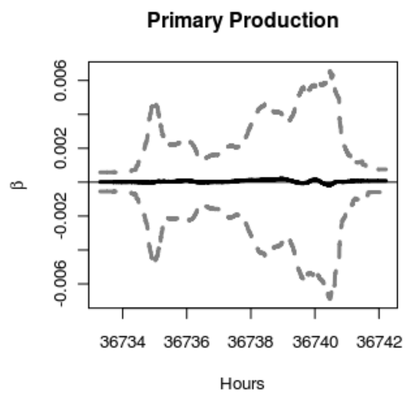
Primary Production
β
Hours

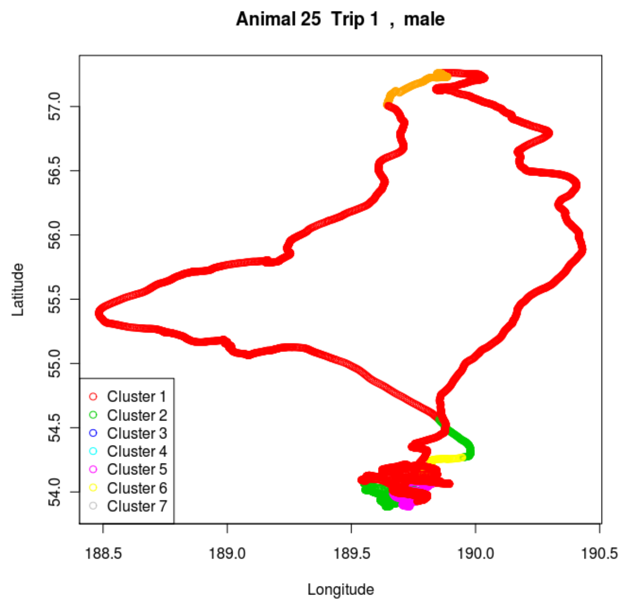
Animal 25 Trip 1 , male
Cluster 1
Cluster 2
Cluster 3
Cluster 4
Cluster 5
Cluster 6
Cluster 7
Latitude
Longitude

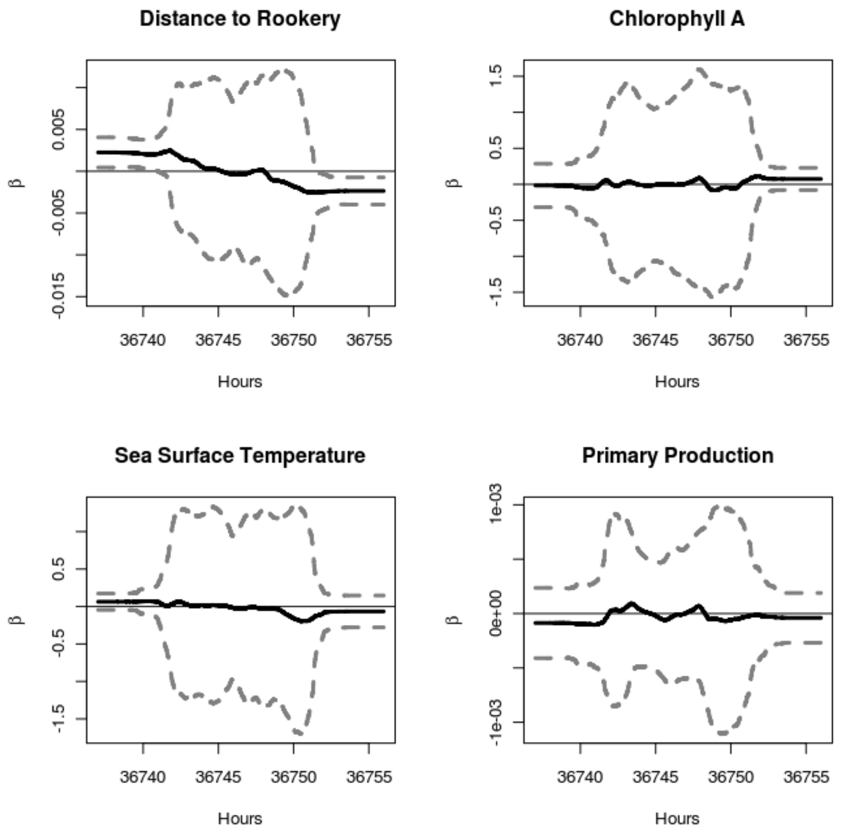
Distance to Rookery
Chlorophyll A
Sea Surface Temperature
Primary Production
β
Hours

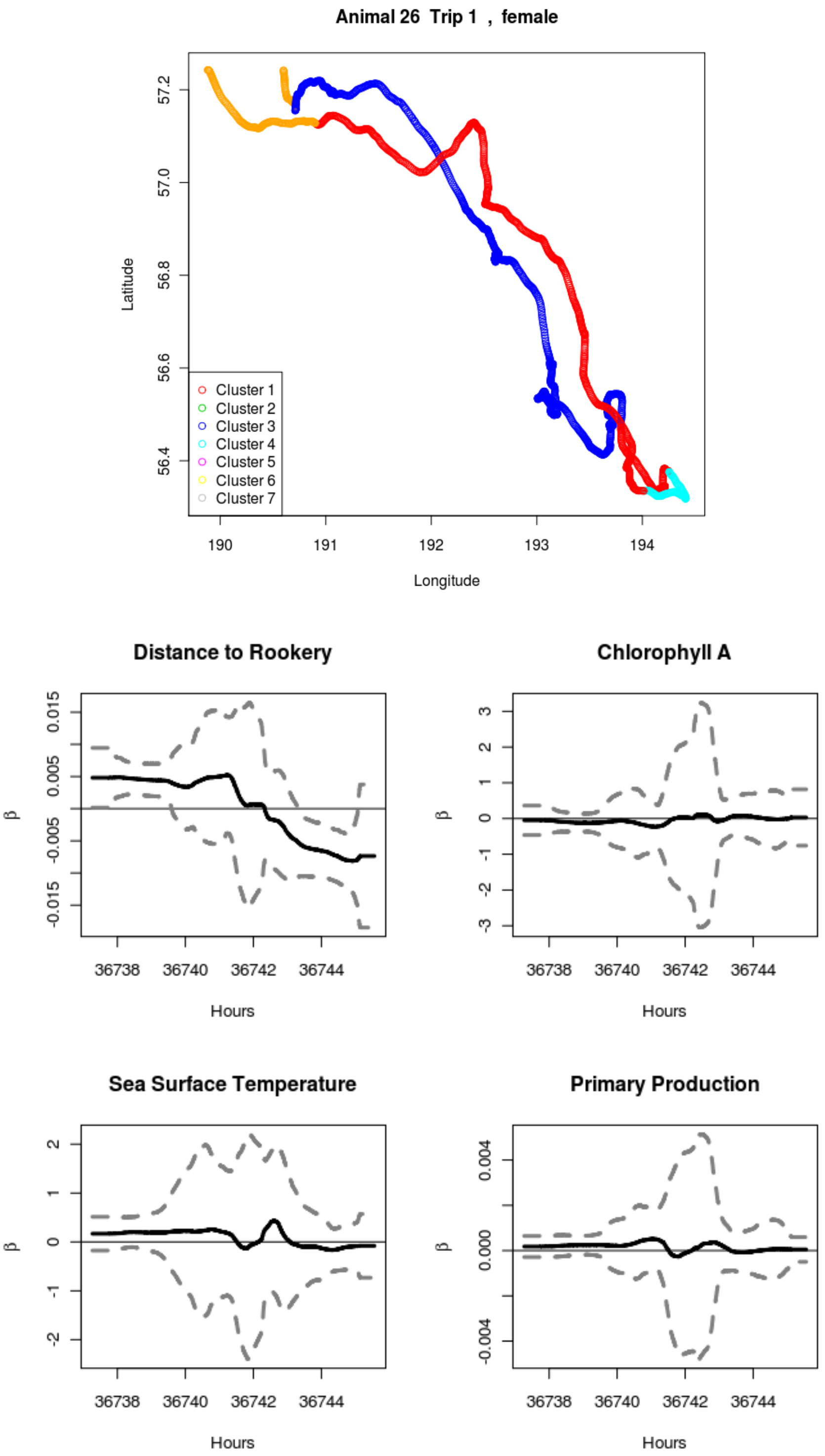

Animal 26 Trip 1 , female
Latitude
Longitude
Cluster 1
Cluster 2
Cluster 3
Cluster 4
Cluster 5
Cluster 6
Cluster 7
Distance to Rookery
Chlorophyll A
Sea Surface Temperature
Primary Production
β
Hours

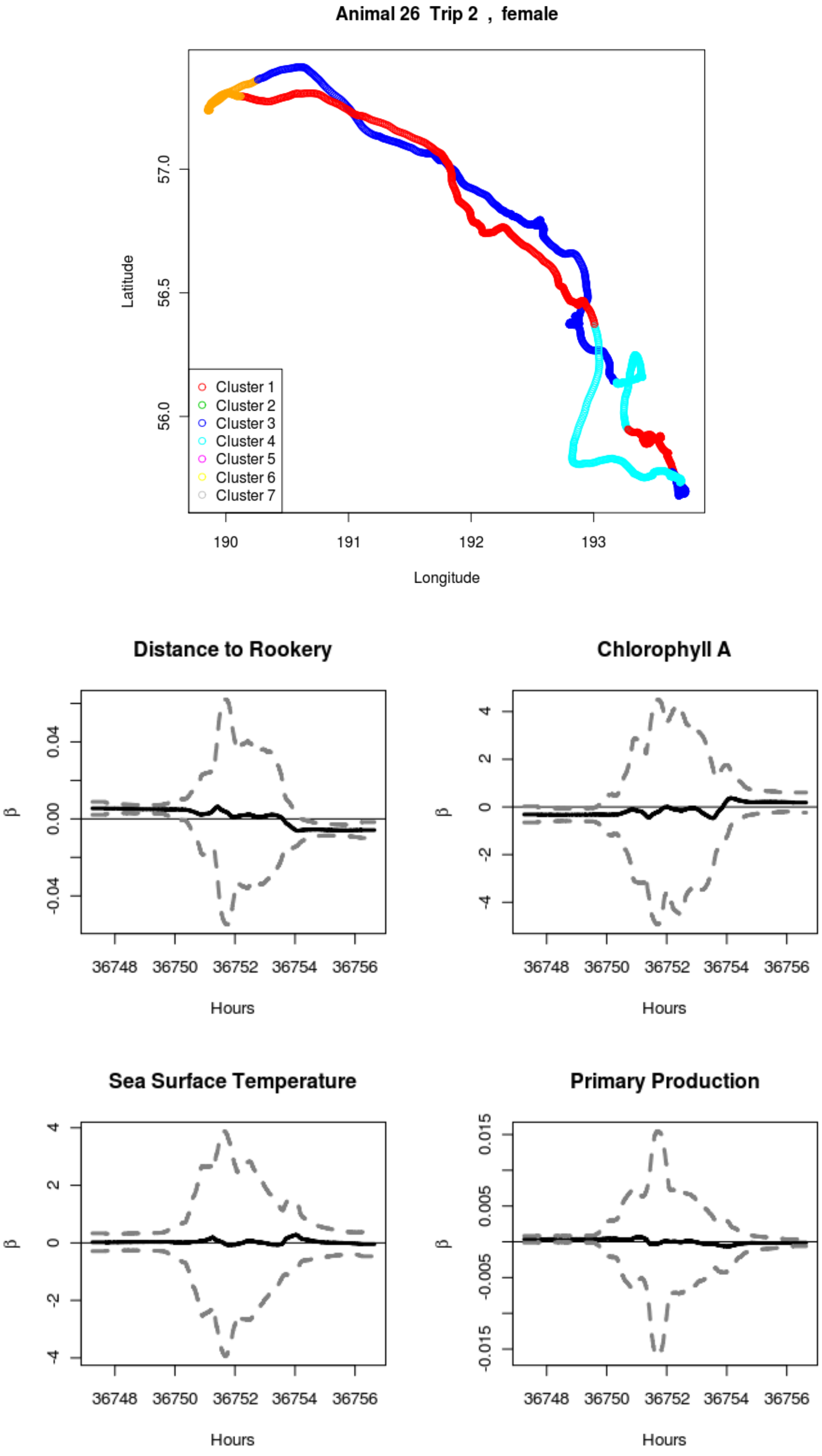
Animal 26 Trip 2 , female
Latitude
57.0
56.5
56.0
Cluster 1
Cluster 2
Cluster 3
Cluster 4
Cluster 5
Cluster 6
Cluster 7
190
191
192
193
Longitude
Distance to Rookery
β
0.04
0.00
-0.04
36748
36750
36752
36754
36756
Hours
Chlorophyll A
β
4
2
0
-2
-4
36748
36750
36752
36754
36756
Hours
Sea Surface Temperature
β
4
2
0
-2
-4
36748
36750
36752
36754
36756
Hours
Primary Production
β
0.015
0.005
-0.005
-0.015
36748
36750
36752
36754
36756
Hours

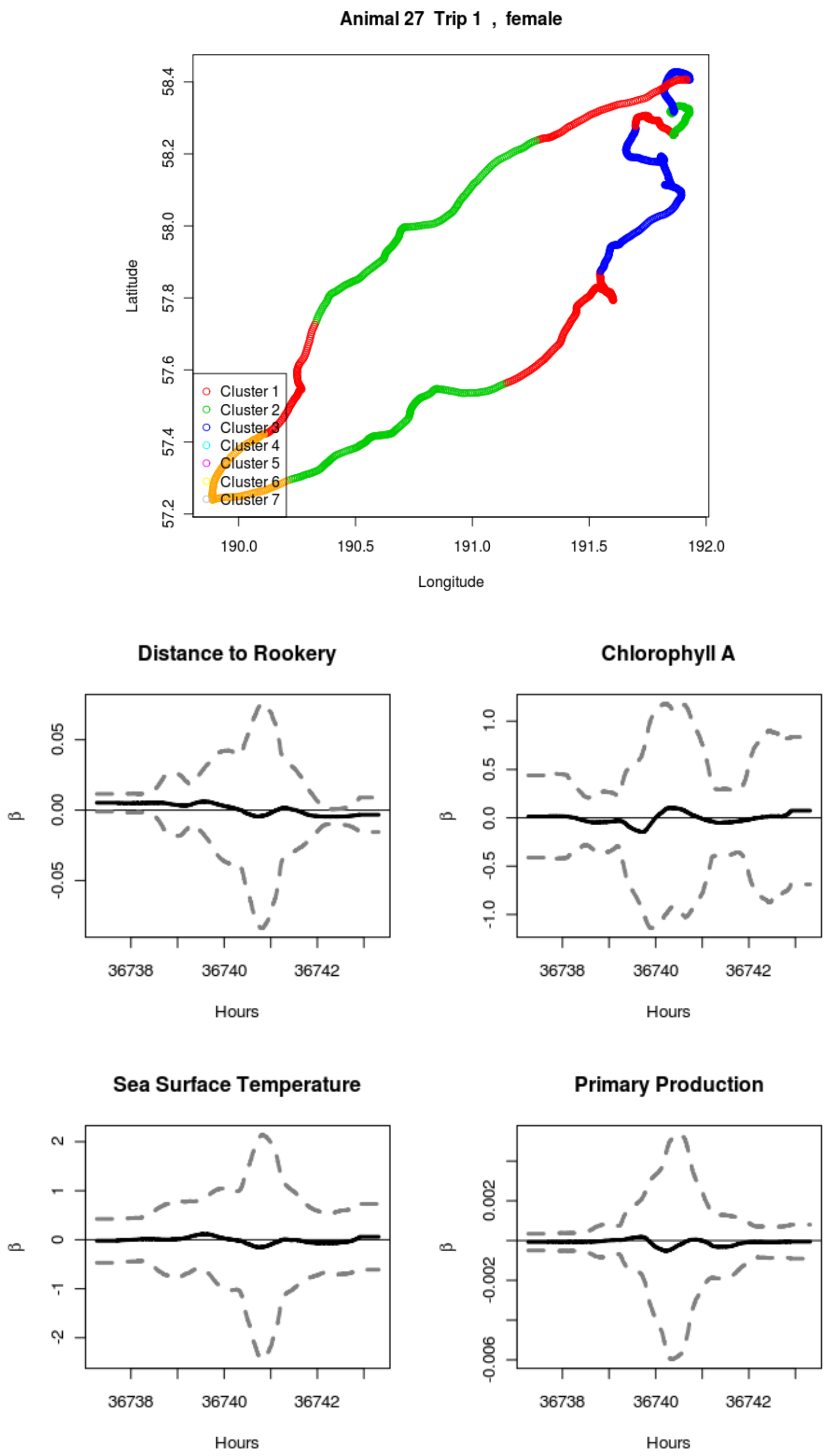
Animal 27 Trip 1 , female
Latitude
Longitude
58.4
58.2
58.0
57.8
57.6
57.4
57.2
190.0
190.5
191.0
191.5
192.0
Cluster 1
Cluster 2
Cluster 3
Cluster 4
Cluster 5
Cluster 6
Cluster 7
Distance to Rookery
Chlorophyll A
Sea Surface Temperature
Primary Production
β
Hours
36738
36740
36742
0.05
0.00
-0.05
1.0
0.5
0.0
-0.5
-1.0
2
1
0
-1
-2
0.002
-0.002
-0.006

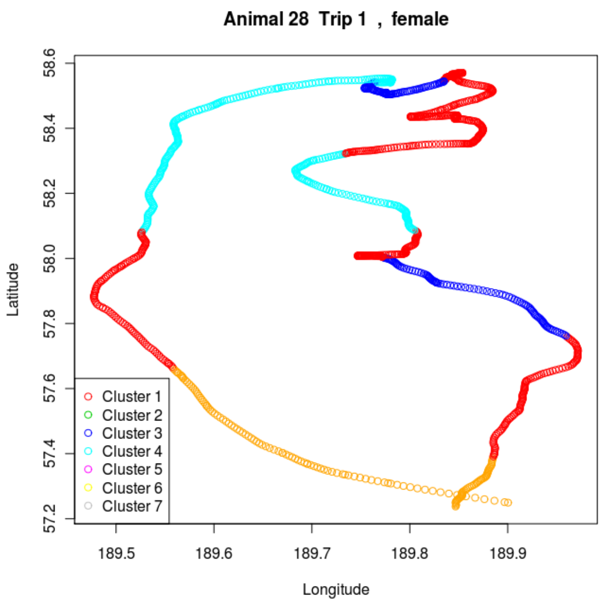
Animal 28 Trip 1 , female
Latitude
Longitude
58.6
58.4
58.2
58.0
57.8
57.6
57.4
57.2
189.5
189.6
189.7
189.8
189.9
Cluster 1
Cluster 2
Cluster 3
Cluster 4
Cluster 5
Cluster 6
Cluster 7

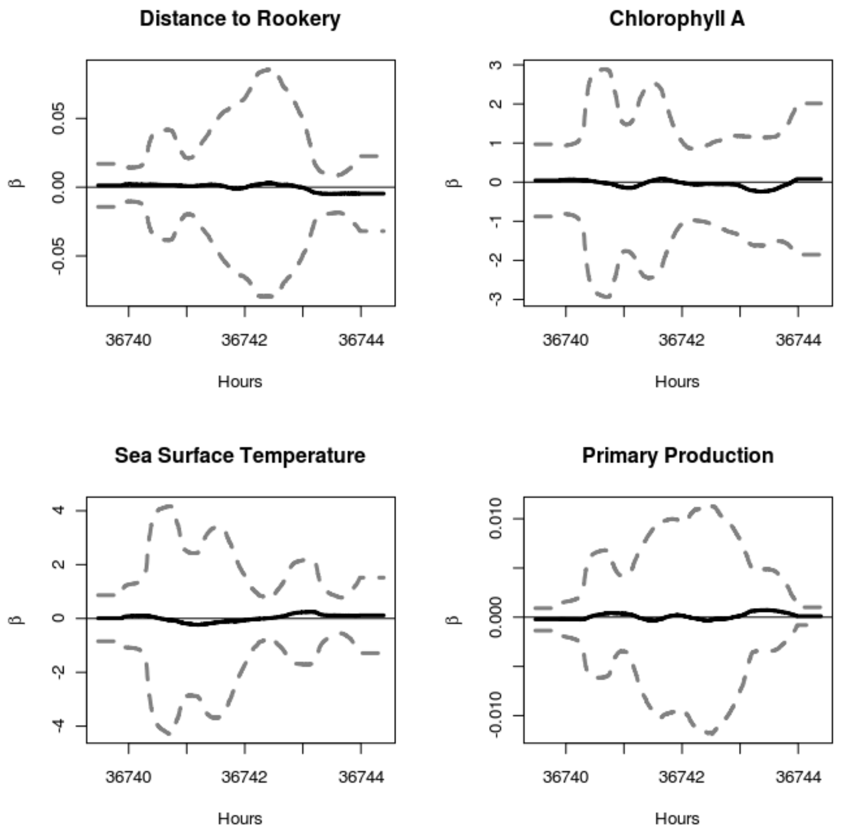
Distance to Rookery
Chlorophyll A
Sea Surface Temperature
Primary Production
β
Hours
36740
36742
36744
0.05
0.00
-0.05
3
2
1
0
-1
-2
-3
4
2
0
-2
-4
0.010
0.000
-0.010

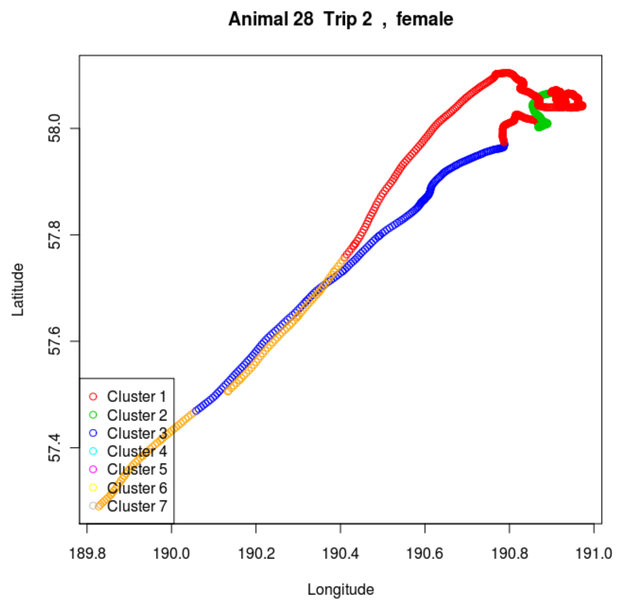
Animal 28 Trip 2 , female
Latitude
58.0
57.8
57.6
57.4
Cluster 1
Cluster 2
Cluster 3
Cluster 4
Cluster 5
Cluster 6
Cluster 7
189.8
190.0
190.2
190.4
190.6
190.8
191.0
Longitude

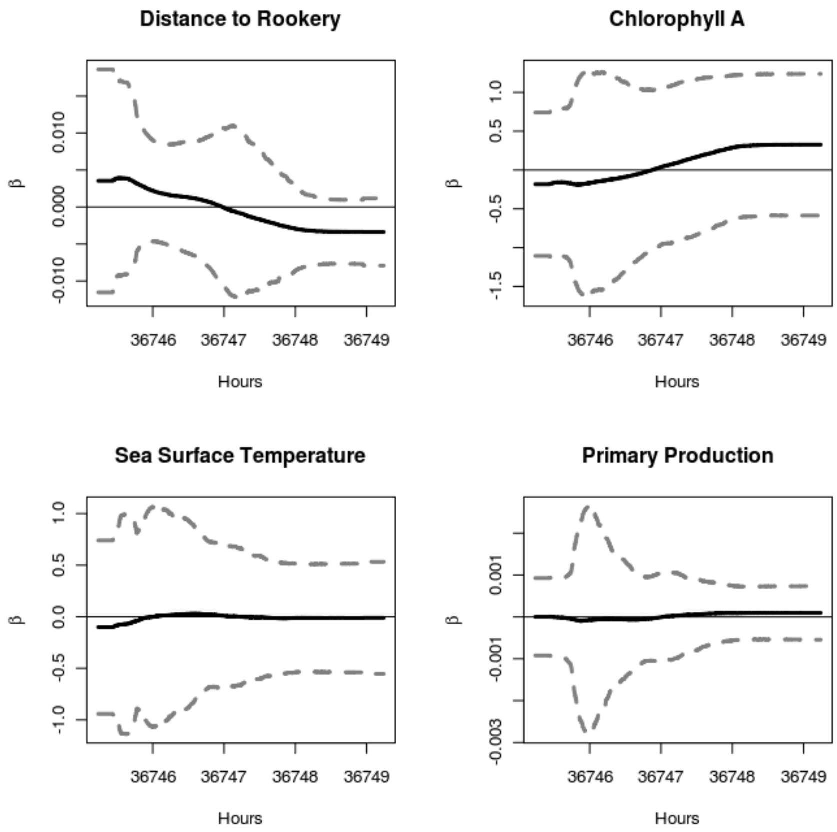
Distance to Rookery
β
0.010
0.000
-0.010
36746
36747
36748
36749
Hours
Chlorophyll A
β
1.0
0.5
-0.5
-1.5
36746
36747
36748
36749
Hours
Sea Surface Temperature
β
1.0
0.5
0.0
-0.5
-1.0
36746
36747
36748
36749
Hours
Primary Production
β
0.001
-0.001
-0.003
36746
36747
36748
36749
Hours

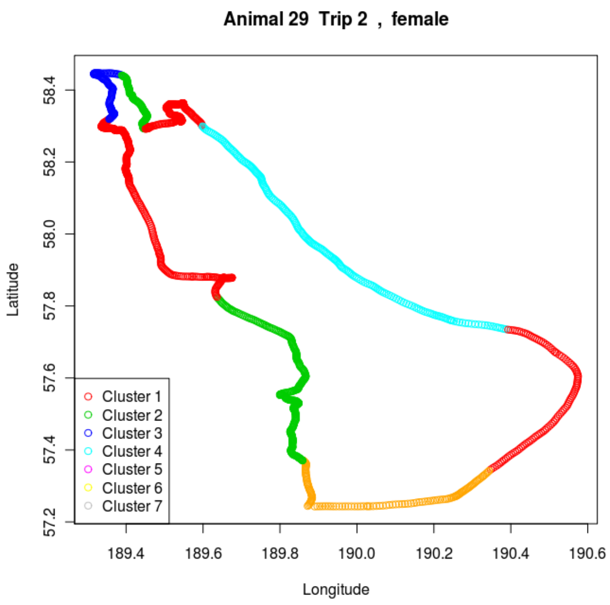
Animal 29 Trip 2 , female
Latitude
Longitude
Cluster 1
Cluster 2
Cluster 3
Cluster 4
Cluster 5
Cluster 6
Cluster 7
57.2
57.4
57.6
57.8
58.0
58.2
58.4
189.4
189.6
189.8
190.0
190.2
190.4
190.6

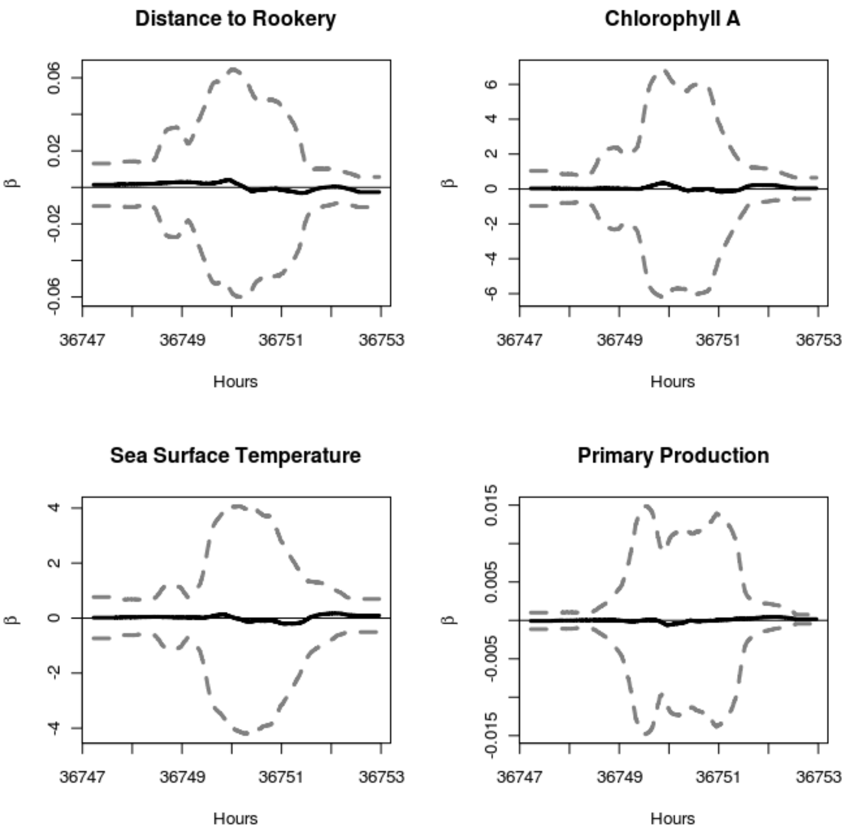
Distance to Rookery
Chlorophyll A
Sea Surface Temperature
Primary Production
β
Hours
36747
36749
36751
36753
-0.06
-0.02
0.02
0.06
-6
-4
-2
0
2
4
6
-4
-2
0
2
4
-0.015
-0.005
0.005
0.015

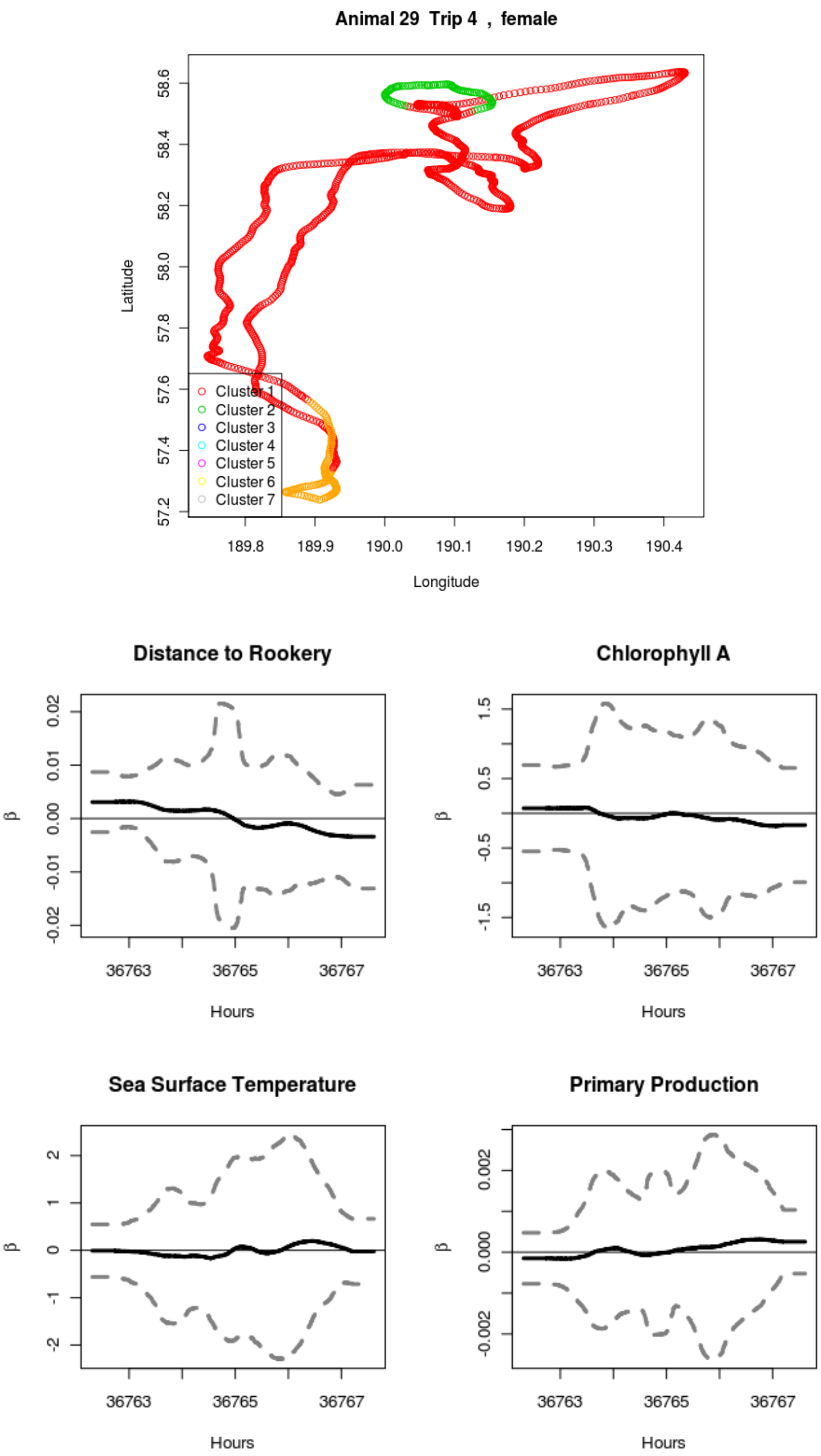
Animal 29 Trip 4 , female
Latitude
58.6
58.4
58.2
58.0
57.8
57.6
57.4
57.2
Cluster 1
Cluster 2
Cluster 3
Cluster 4
Cluster 5
Cluster 6
Cluster 7
189.8
189.9
190.0
190.1
190.2
190.3
190.4
Longitude
Distance to Rookery
β
0.02
0.01
0.00
-0.01
-0.02
36763
36765
36767
Hours
Chlorophyll A
β
1.5
0.5
-0.5
-1.5
36763
36765
36767
Hours
Sea Surface Temperature
β
2
1
0
-1
-2
36763
36765
36767
Hours
Primary Production
β
0.002
0.000
-0.002
36763
36765
36767
Hours

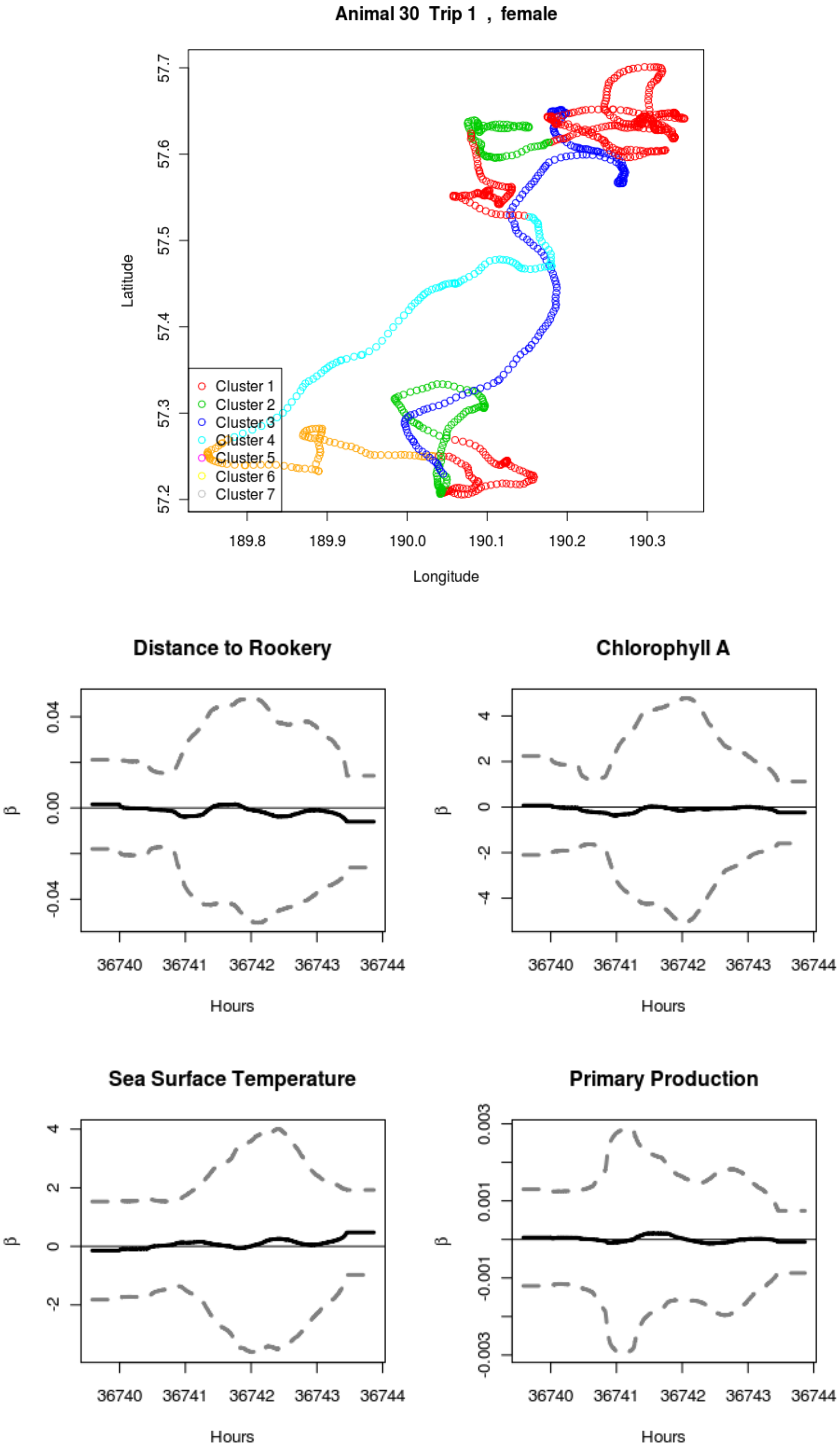
Animal 30 Trip 1 , female
Latitude
Longitude
57.7
57.6
57.5
57.4
57.3
57.2
189.8
189.9
190.0
190.1
190.2
190.3
Cluster 1
Cluster 2
Cluster 3
Cluster 4
Cluster 5
Cluster 6
Cluster 7
Distance to Rookery
Chlorophyll A
Sea Surface Temperature
Primary Production
β
Hours
0.04
0.00
-0.04
4
2
0
-2
-4
0.003
0.001
-0.001
-0.003
36740
36741
36742
36743
36744

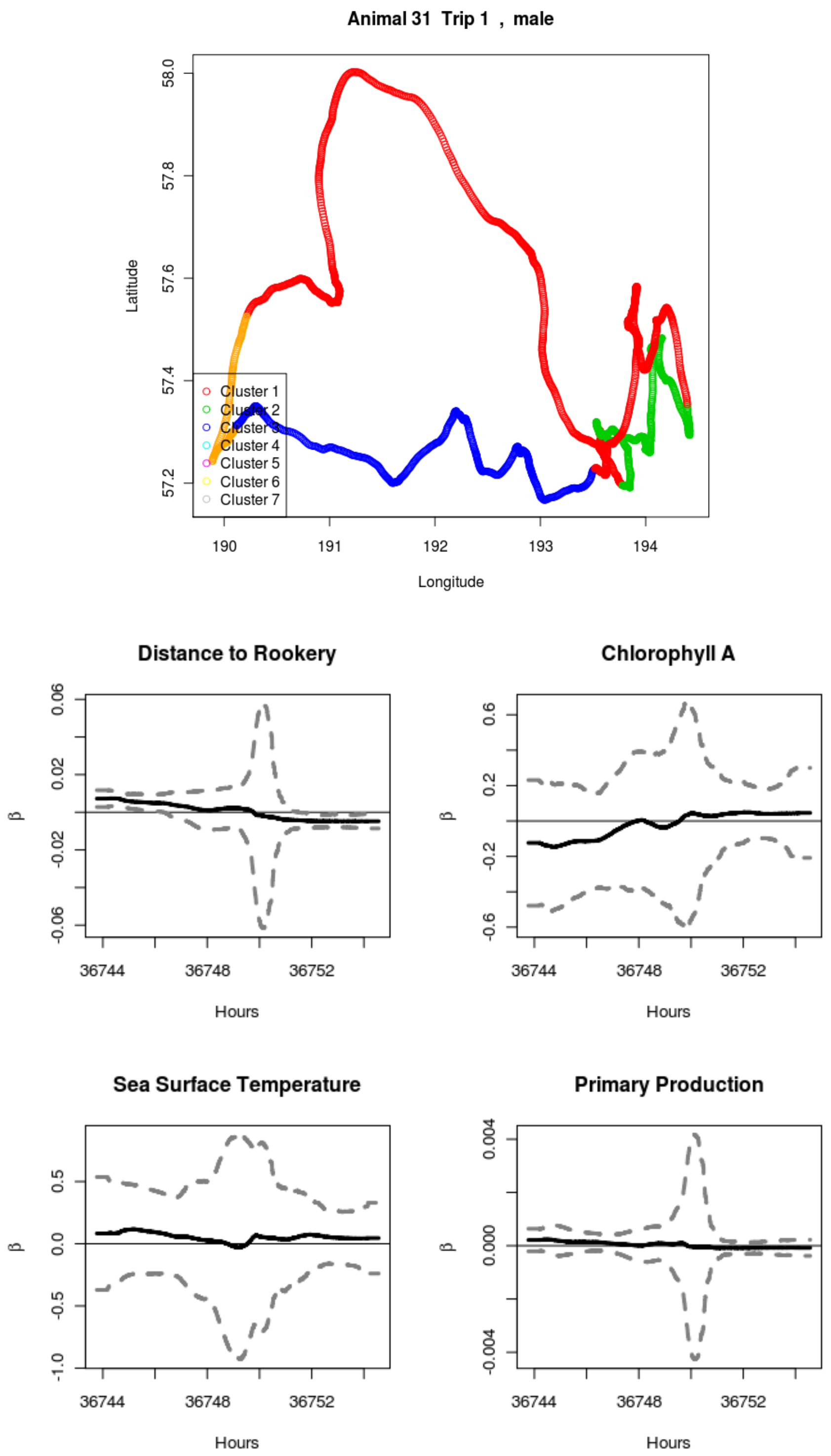
Animal 31 Trip 1 , male
Latitude
Longitude
Cluster 1
Cluster 2
Cluster 3
Cluster 4
Cluster 5
Cluster 6
Cluster 7
Distance to Rookery
Chlorophyll A
Sea Surface Temperature
Primary Production
β
Hours

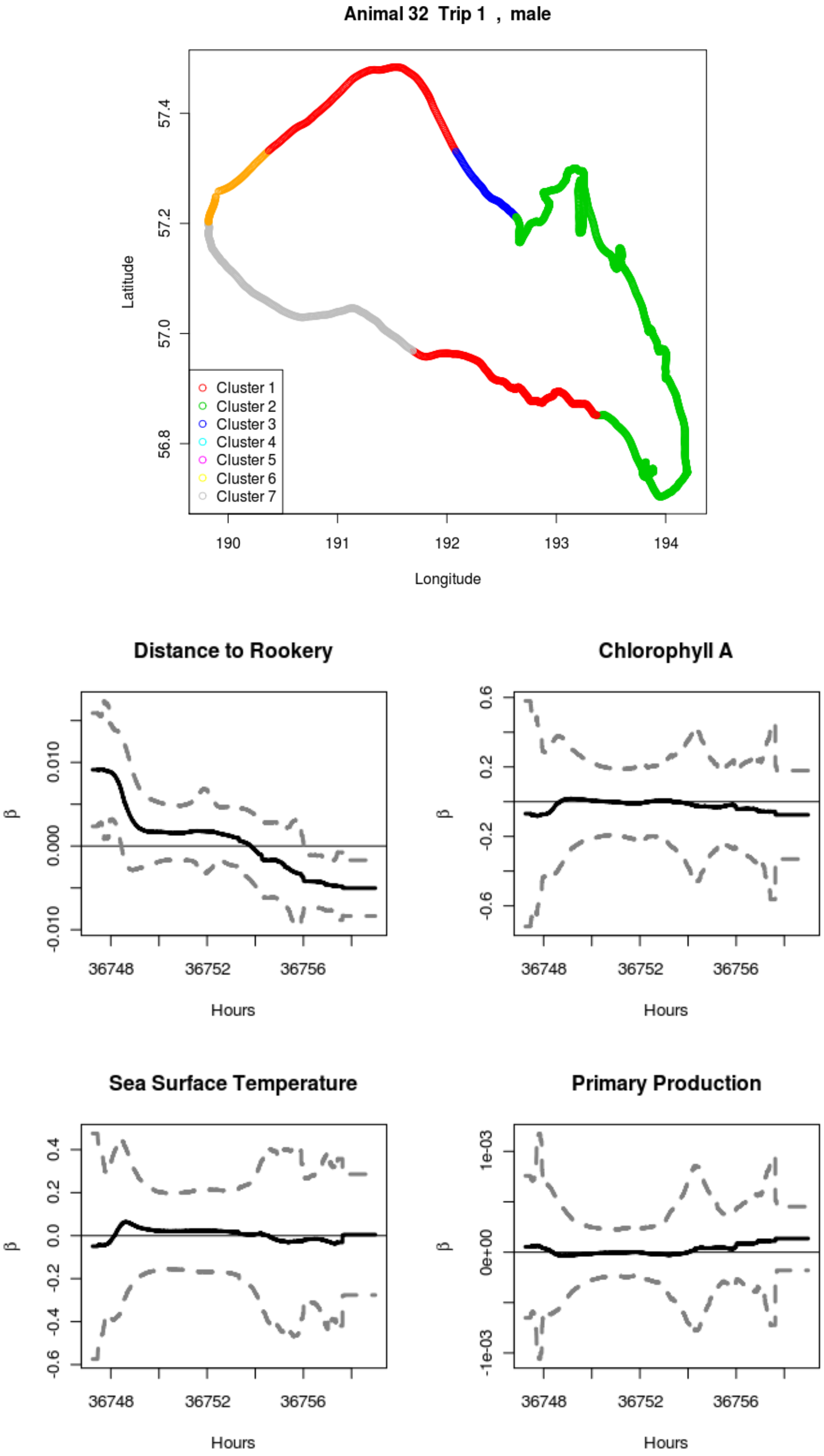
Animal 32 Trip 1 , male
Latitude
Longitude
Cluster 1
Cluster 2
Cluster 3
Cluster 4
Cluster 5
Cluster 6
Cluster 7
Distance to Rookery
Chlorophyll A
Sea Surface Temperature
Primary Production
β
Hours

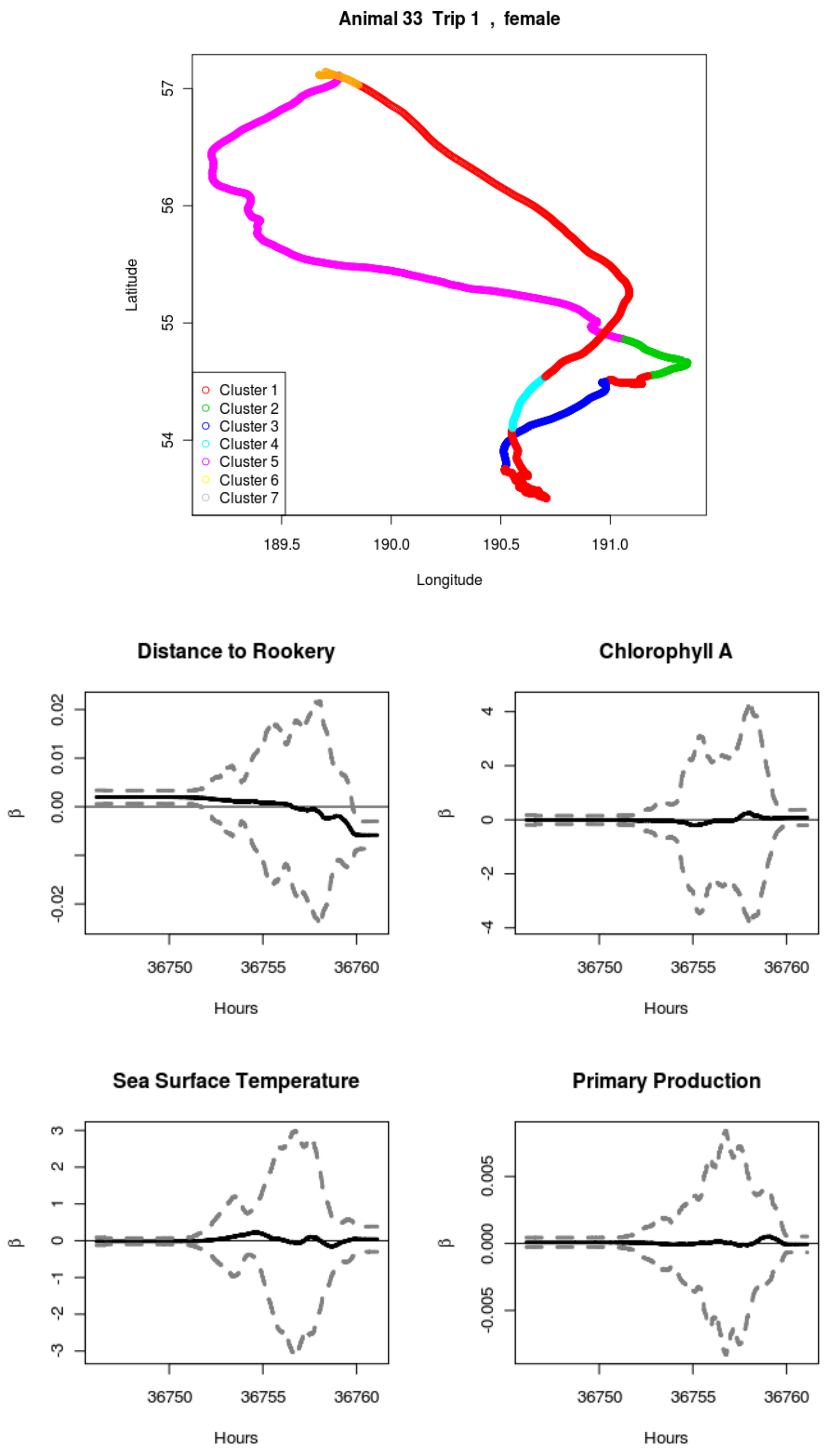
Animal 33 Trip 1 , female
Latitude
Longitude
Cluster 1
Cluster 2
Cluster 3
Cluster 4
Cluster 5
Cluster 6
Cluster 7
Distance to Rookery
Chlorophyll A
Sea Surface Temperature
Primary Production
β
Hours

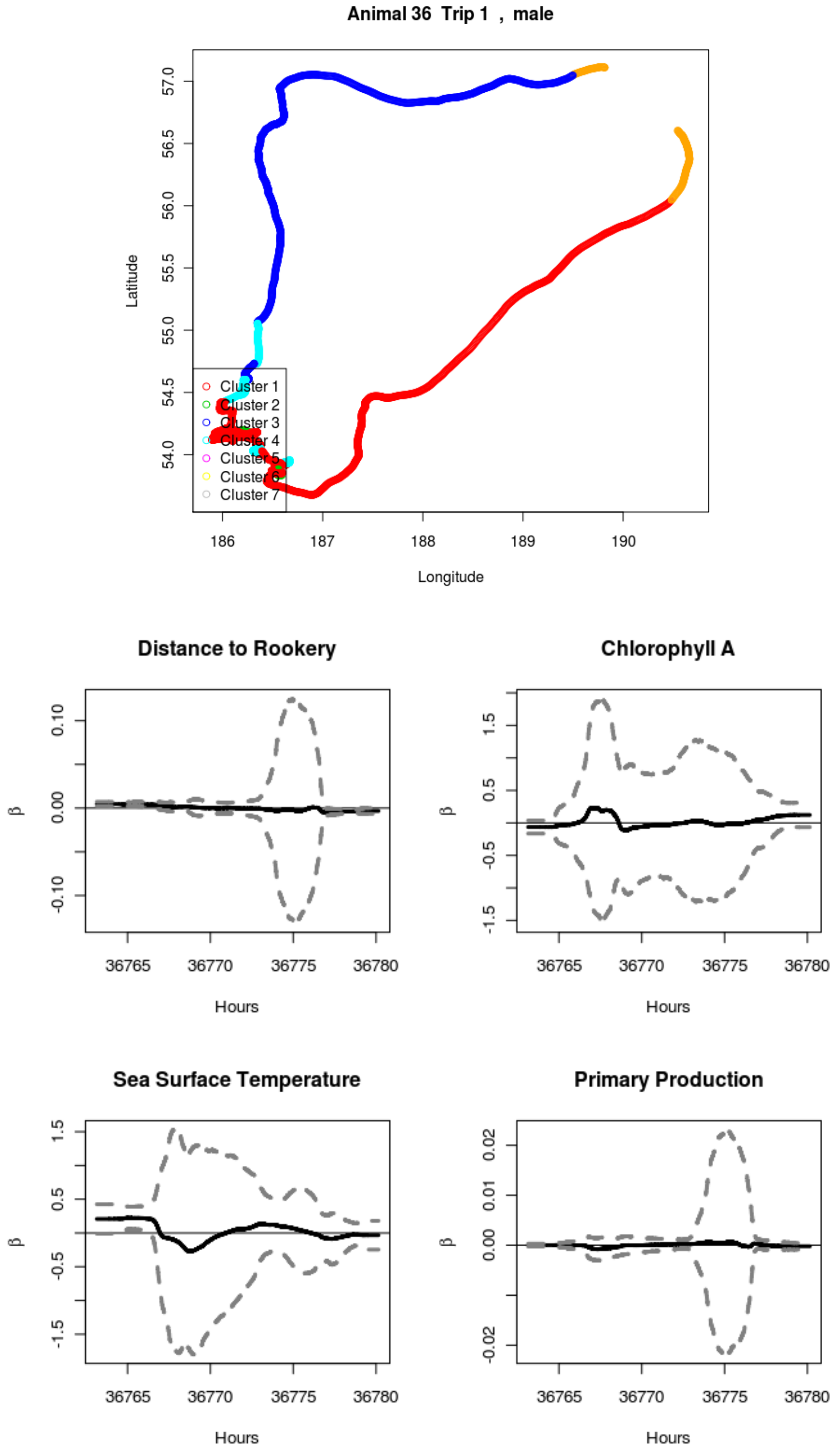
Animal 36 Trip 1 , male
Latitude
Longitude
Cluster 1
Cluster 2
Cluster 3
Cluster 4
Cluster 5
Cluster 6
Cluster 7
Distance to Rookery
Chlorophyll A
Sea Surface Temperature
Primary Production
β
Hours

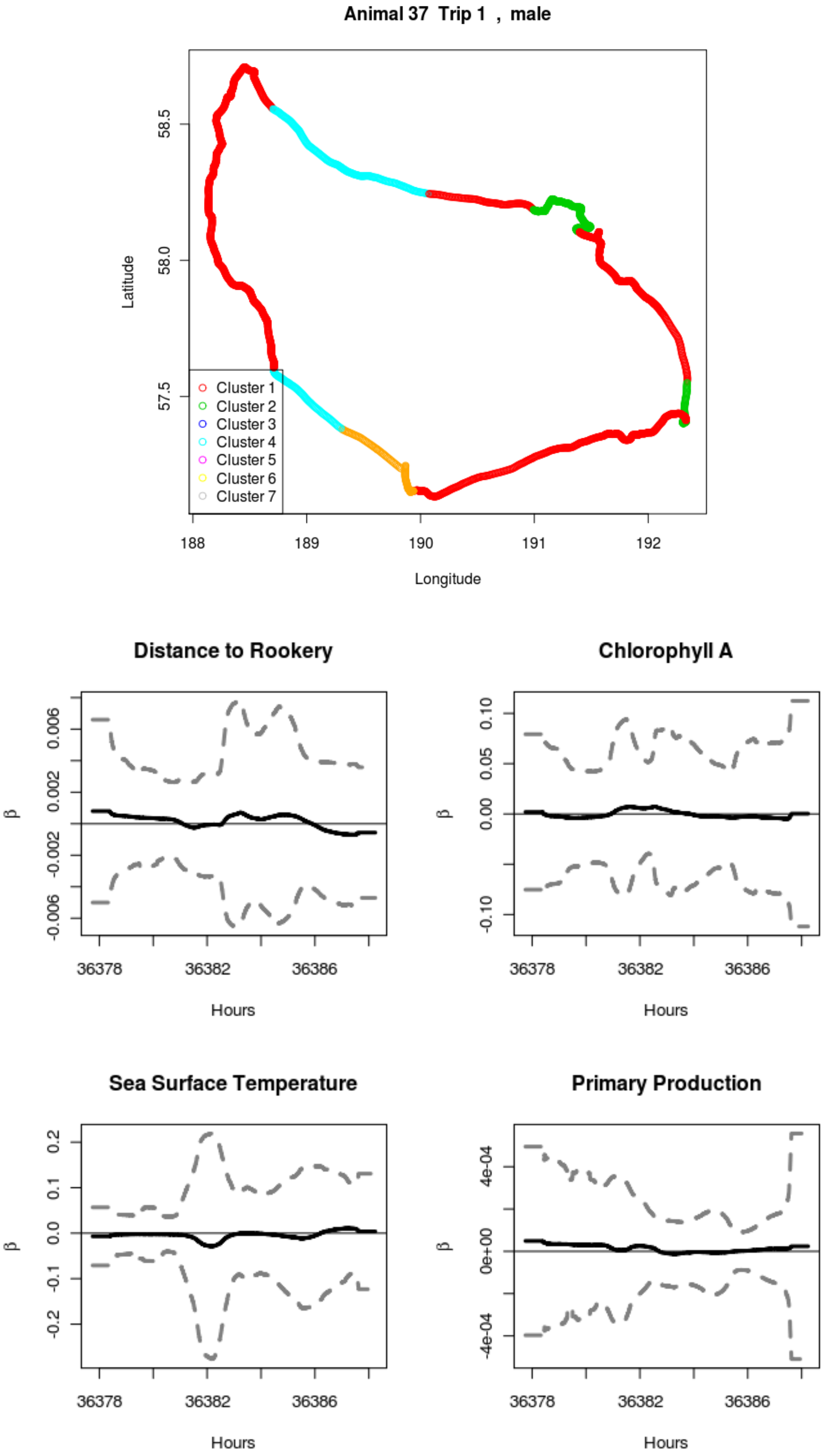
Animal 37 Trip 1 , male
Latitude
Longitude
58.5
58.0
57.5
188
189
190
191
192
Cluster 1
Cluster 2
Cluster 3
Cluster 4
Cluster 5
Cluster 6
Cluster 7
Distance to Rookery
Chlorophyll A
Sea Surface Temperature
Primary Production
β
Hours
36378
36382
36386
0.006
0.002
-0.002
-0.006
0.10
0.05
0.00
-0.10
0.2
0.1
0.0
-0.1
-0.2
4e-04
0e+00
-4e-04

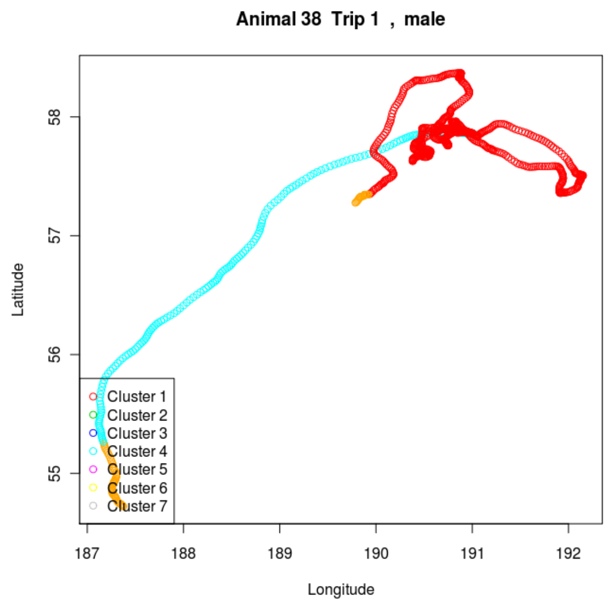
Animal 38 Trip 1 , male
Latitude
Longitude
Cluster 1
Cluster 2
Cluster 3
Cluster 4
Cluster 5
Cluster 6
Cluster 7
187
188
189
190
191
192
55
56
57
58

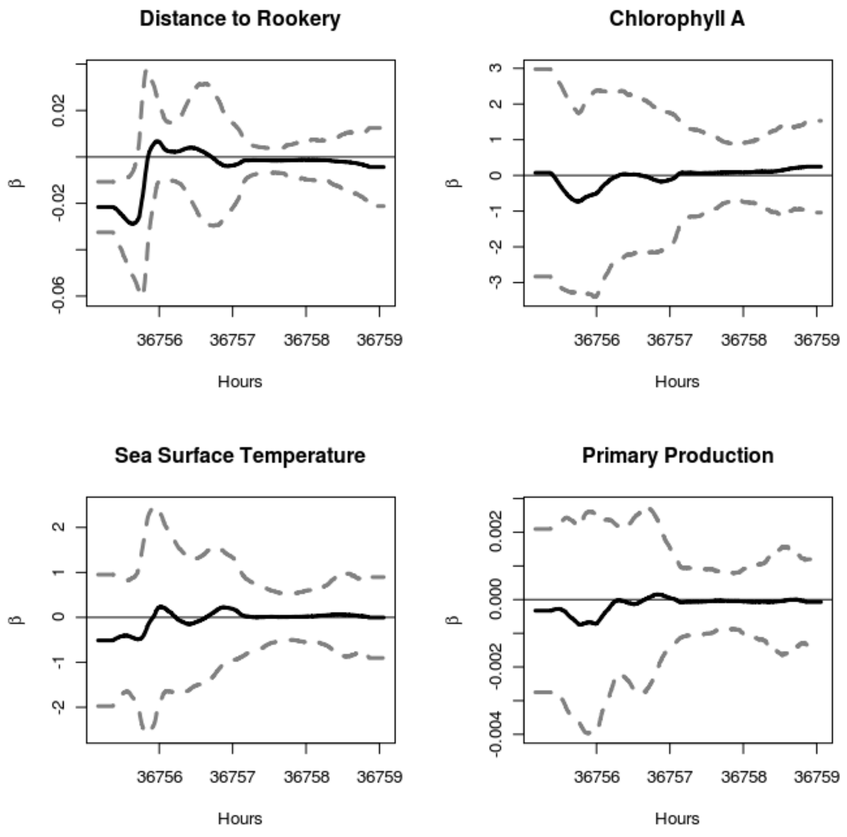
Distance to Rookery
Chlorophyll A
Sea Surface Temperature
Primary Production
β
Hours
36756
36757
36758
36759

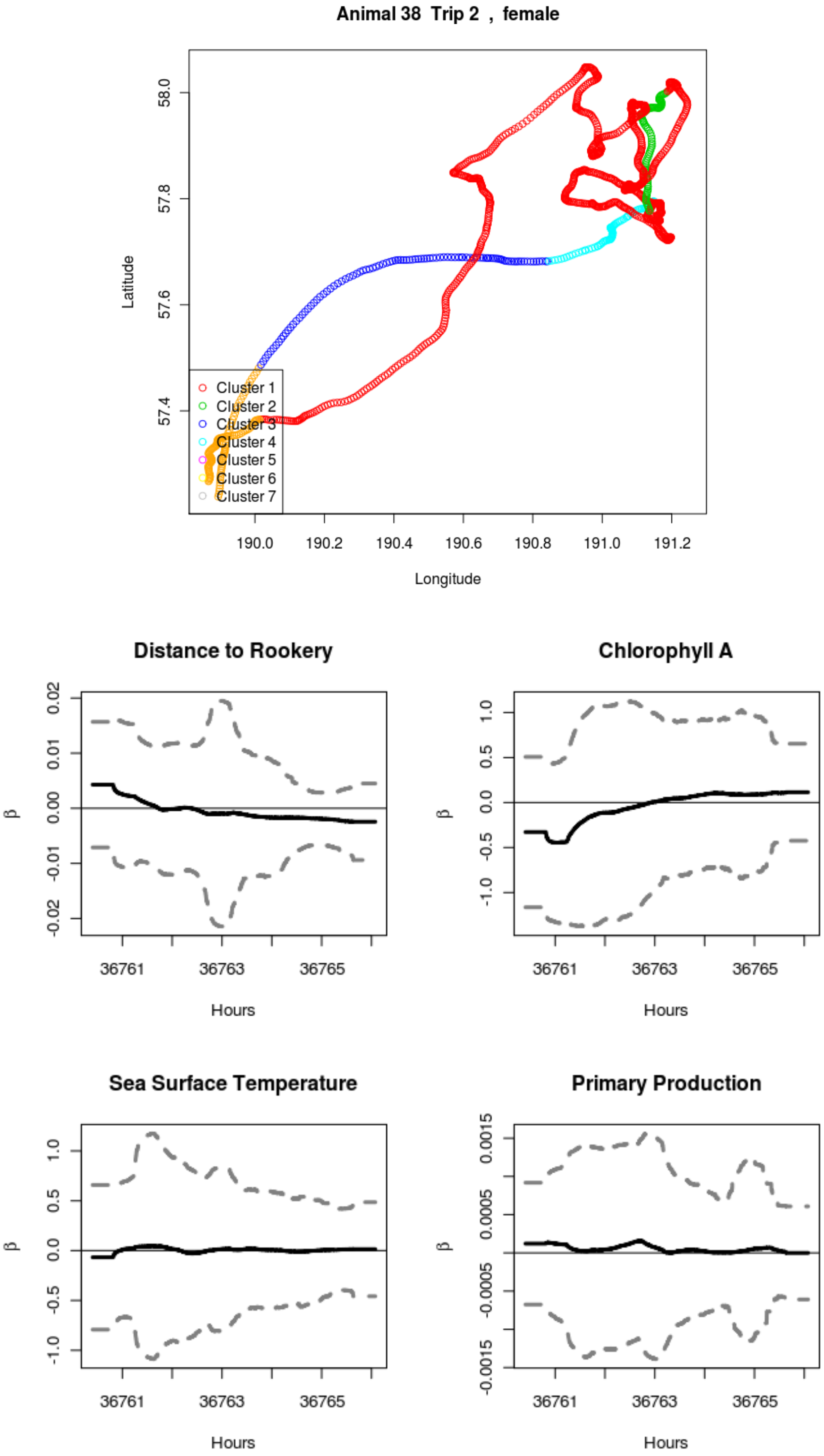
Animal 38 Trip 2 , female
Latitude
58.0
57.8
57.6
57.4
Longitude
190.0
190.2
190.4
190.6
190.8
191.0
191.2
Cluster 1
Cluster 2
Cluster 3
Cluster 4
Cluster 5
Cluster 6
Cluster 7
Distance to Rookery
Chlorophyll A
Sea Surface Temperature
Primary Production
β
Hours
36761
36763
36765
0.02
0.01
0.00
-0.01
-0.02
1.0
0.5
0.0
-0.5
-1.0
0.0015
0.0005
-0.0005
-0.0015

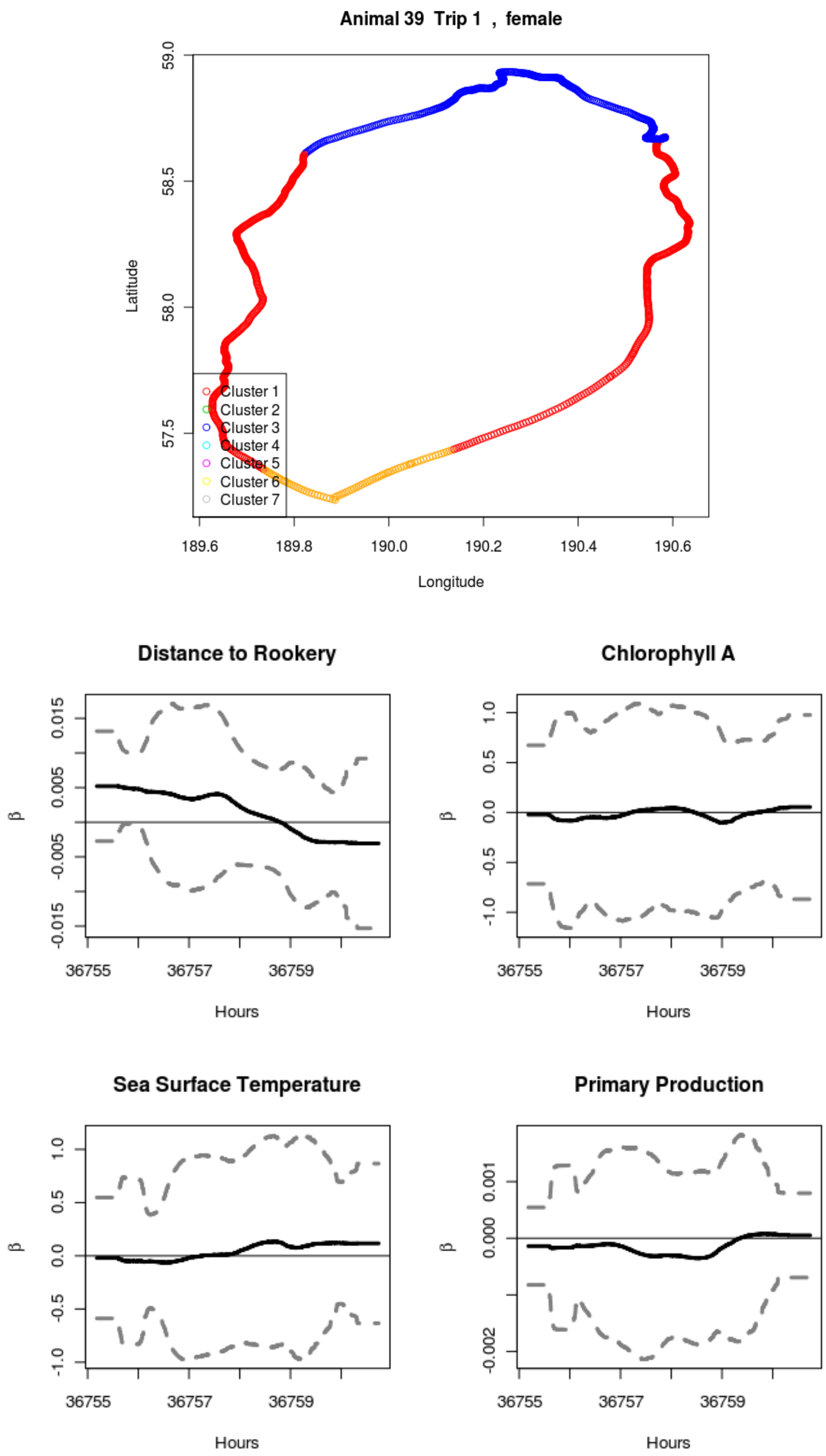
Animal 39 Trip 1 , female
Latitude
Longitude
Cluster 1
Cluster 2
Cluster 3
Cluster 4
Cluster 5
Cluster 6
Cluster 7
Distance to Rookery
Chlorophyll A
Sea Surface Temperature
Primary Production
β
Hours

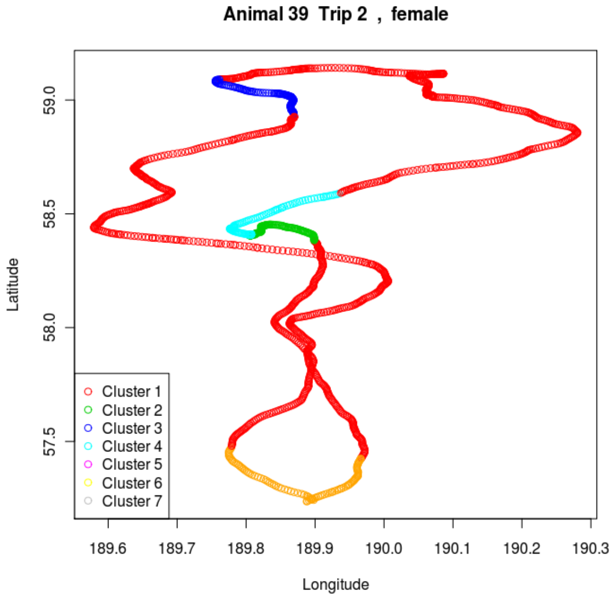
Animal 39 Trip 2 , female
Latitude
Longitude
Cluster 1
Cluster 2
Cluster 3
Cluster 4
Cluster 5
Cluster 6
Cluster 7
59.0
58.5
58.0
57.5
189.6
189.7
189.8
189.9
190.0
190.1
190.2
190.3

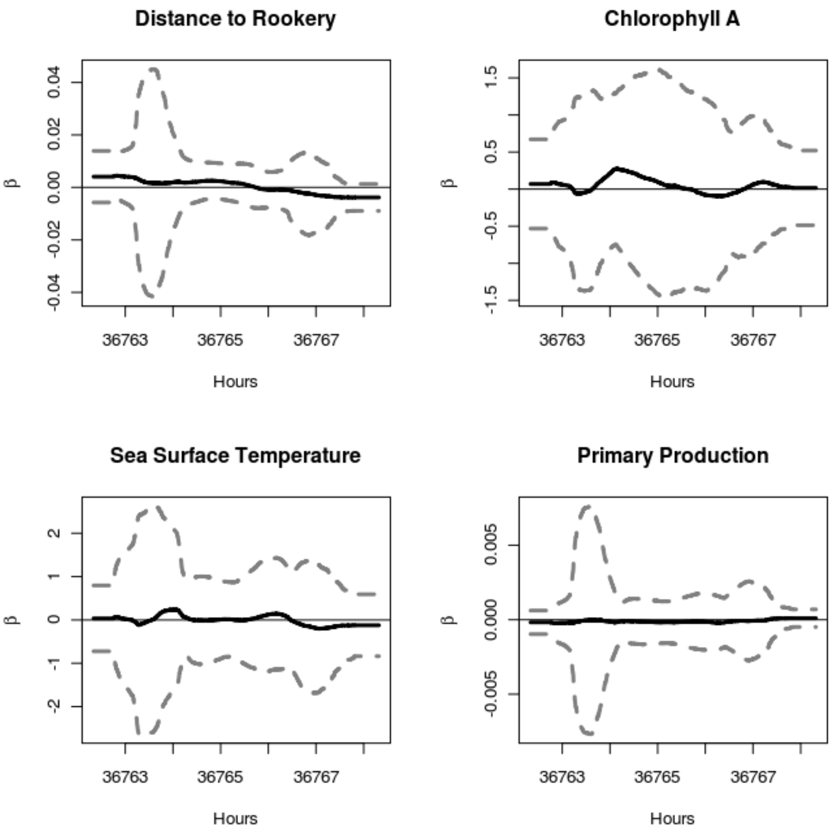
Distance to Rookery
Chlorophyll A
Sea Surface Temperature
Primary Production
β
Hours
36763
36765
36767

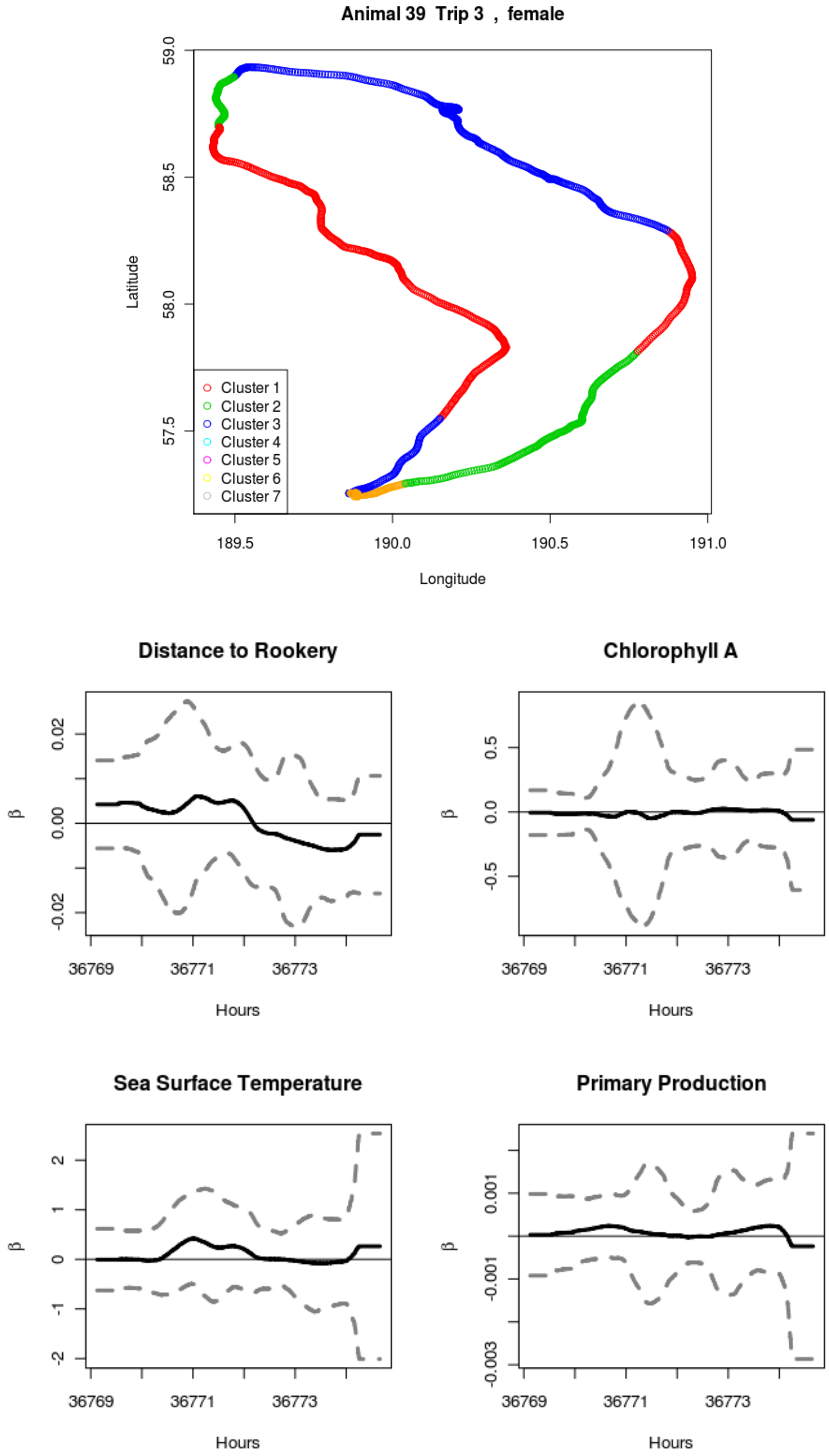
Animal 39 Trip 3 , female
Latitude
Longitude
59.0
58.5
58.0
57.5
189.5
190.0
190.5
191.0
Cluster 1
Cluster 2
Cluster 3
Cluster 4
Cluster 5
Cluster 6
Cluster 7
Distance to Rookery
Chlorophyll A
Sea Surface Temperature
Primary Production
β
Hours
36769
36771
36773
0.02
0.00
-0.02
0.5
0.0
-0.5
2
1
0
-1
-2
0.001
-0.001
-0.003

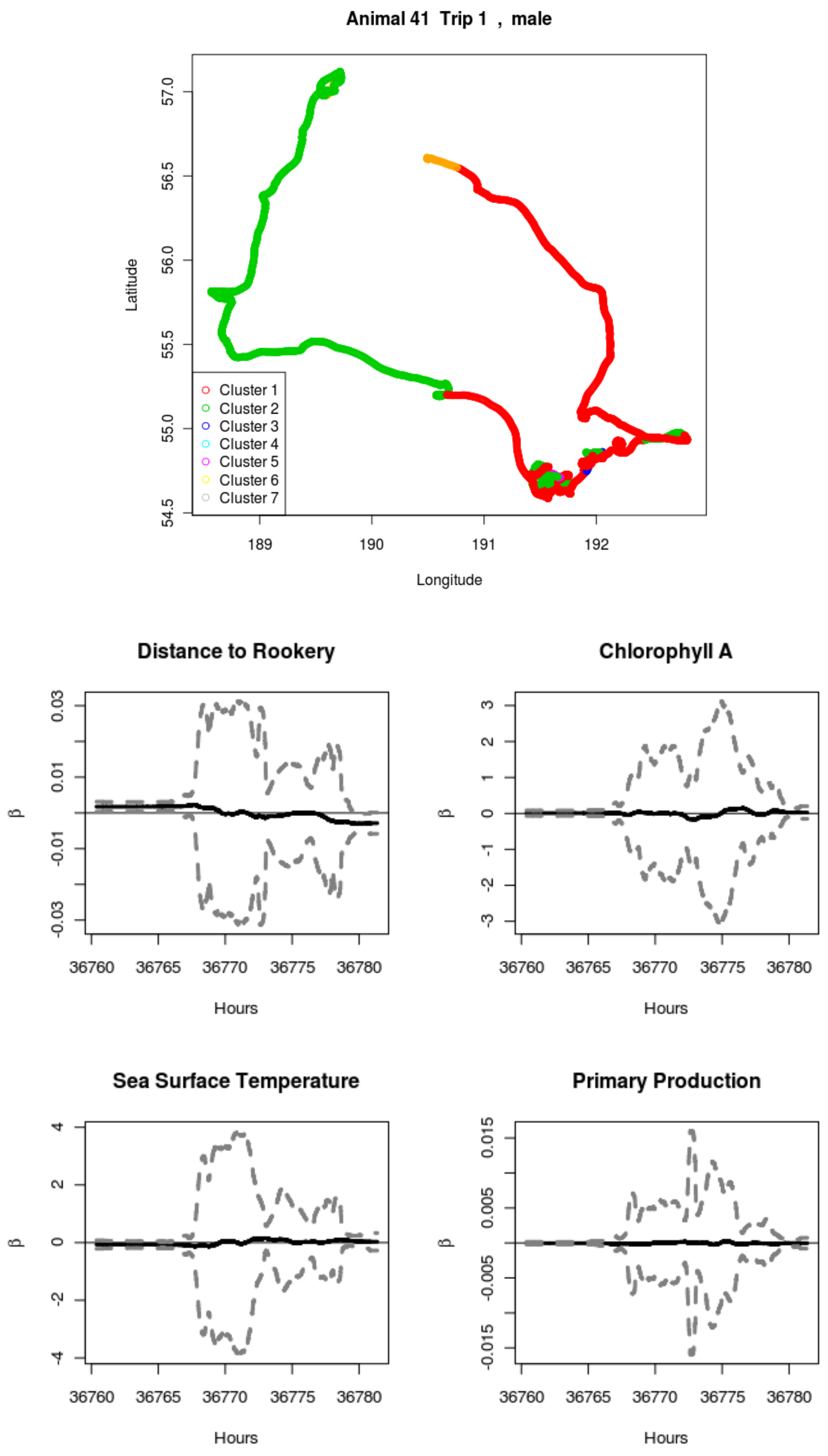
Animal 41 Trip 1 , male
Latitude
Longitude
Cluster 1
Cluster 2
Cluster 3
Cluster 4
Cluster 5
Cluster 6
Cluster 7
Distance to Rookery
Chlorophyll A
Sea Surface Temperature
Primary Production
β
Hours

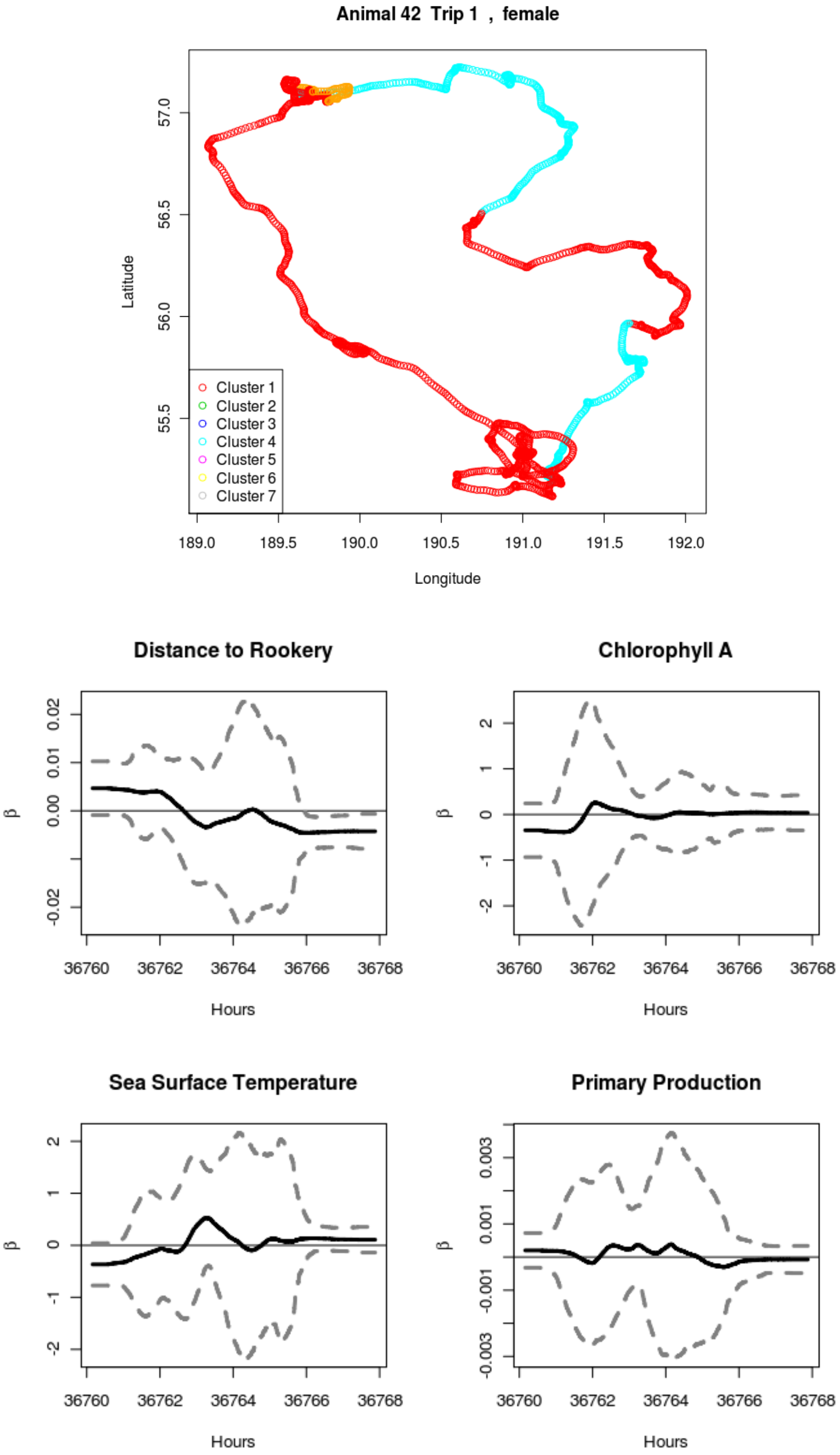
Animal 42 Trip 1 , female
Latitude
Longitude
Cluster 1
Cluster 2
Cluster 3
Cluster 4
Cluster 5
Cluster 6
Cluster 7
Distance to Rookery
Chlorophyll A
Sea Surface Temperature
Primary Production
β
Hours

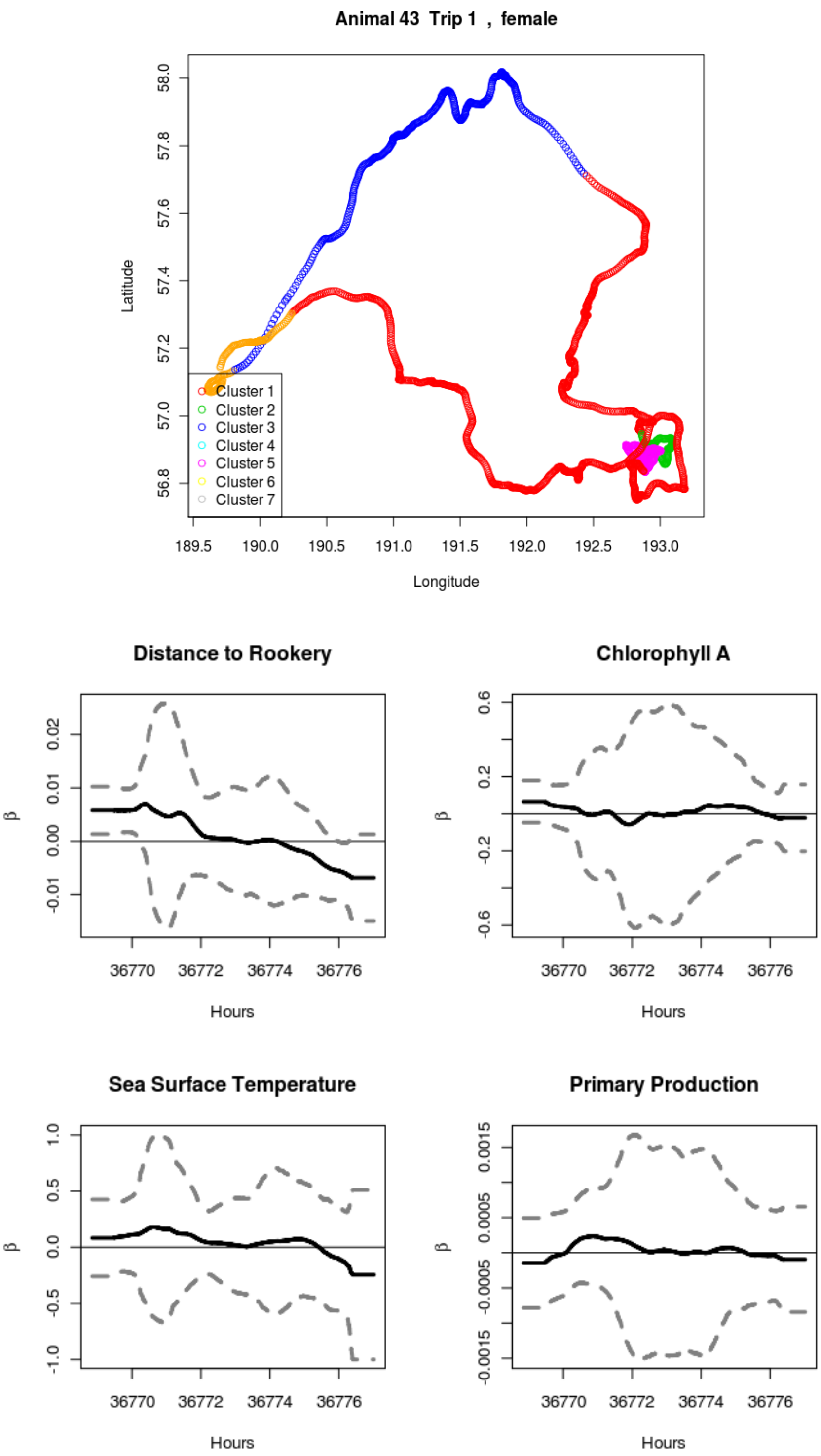
Animal 43 Trip 1 , female
Latitude
58.0
57.8
57.6
57.4
57.2
57.0
56.8
Cluster 1
Cluster 2
Cluster 3
Cluster 4
Cluster 5
Cluster 6
Cluster 7
189.5
190.0
190.5
191.0
191.5
192.0
192.5
193.0
Longitude
Distance to Rookery
β
0.02
0.01
0.00
-0.01
36770
36772
36774
36776
Hours
Chlorophyll A
β
0.6
0.2
-0.2
-0.6
36770
36772
36774
36776
Hours
Sea Surface Temperature
β
1.0
0.5
0.0
-0.5
-1.0
36770
36772
36774
36776
Hours
Primary Production
β
0.0015
0.0005
-0.0005
-0.0015
36770
36772
36774
36776
Hours

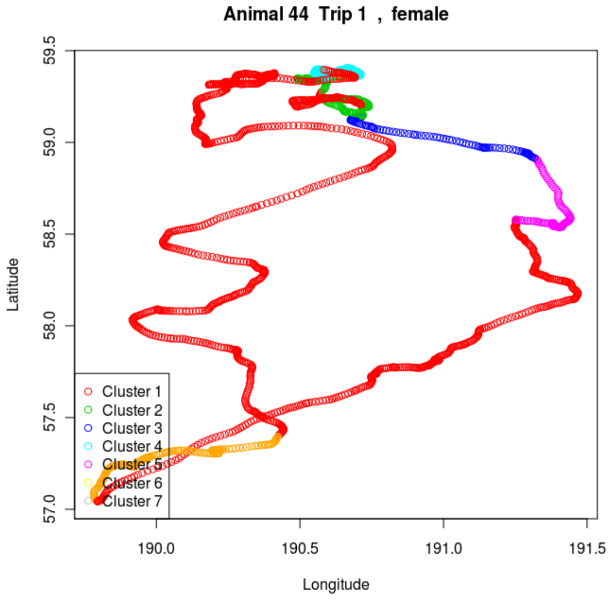
Animal 44 Trip 1 , female
Latitude
Longitude
59.5
59.0
58.5
58.0
57.5
57.0
190.0
190.5
191.0
191.5
Cluster 1
Cluster 2
Cluster 3
Cluster 4
Cluster 5
Cluster 6
Cluster 7

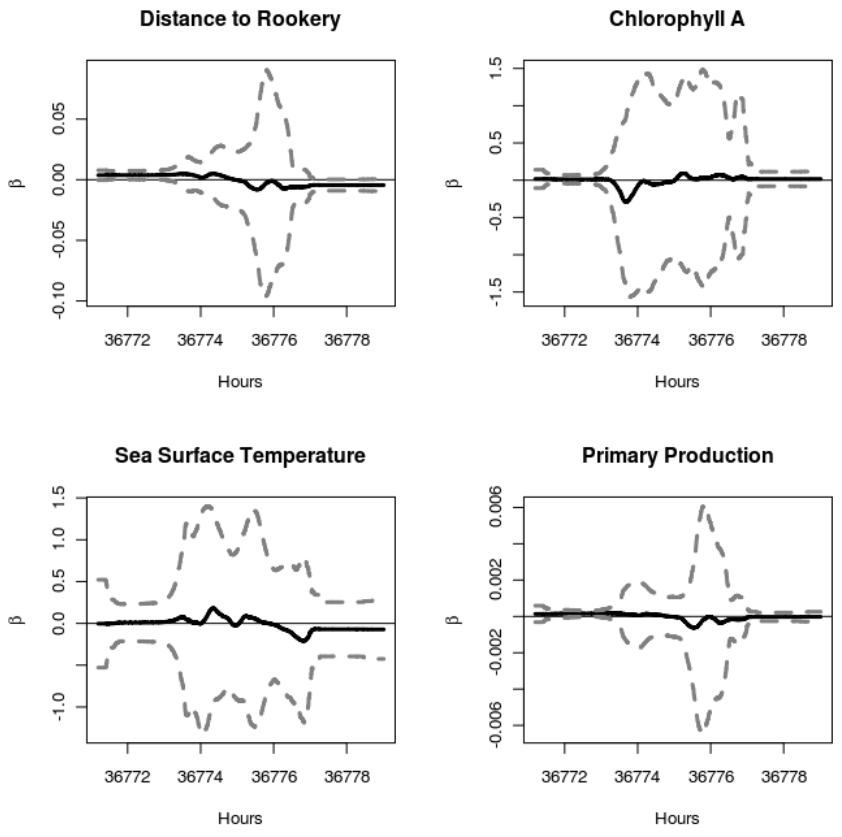
Distance to Rookery
Chlorophyll A
Sea Surface Temperature
Primary Production
β
Hours
36772
36774
36776
36778
0.05
0.00
-0.05
-0.10
1.5
0.5
-0.5
-1.5
1.0
0.0
-1.0
0.006
0.002
-0.002
-0.006

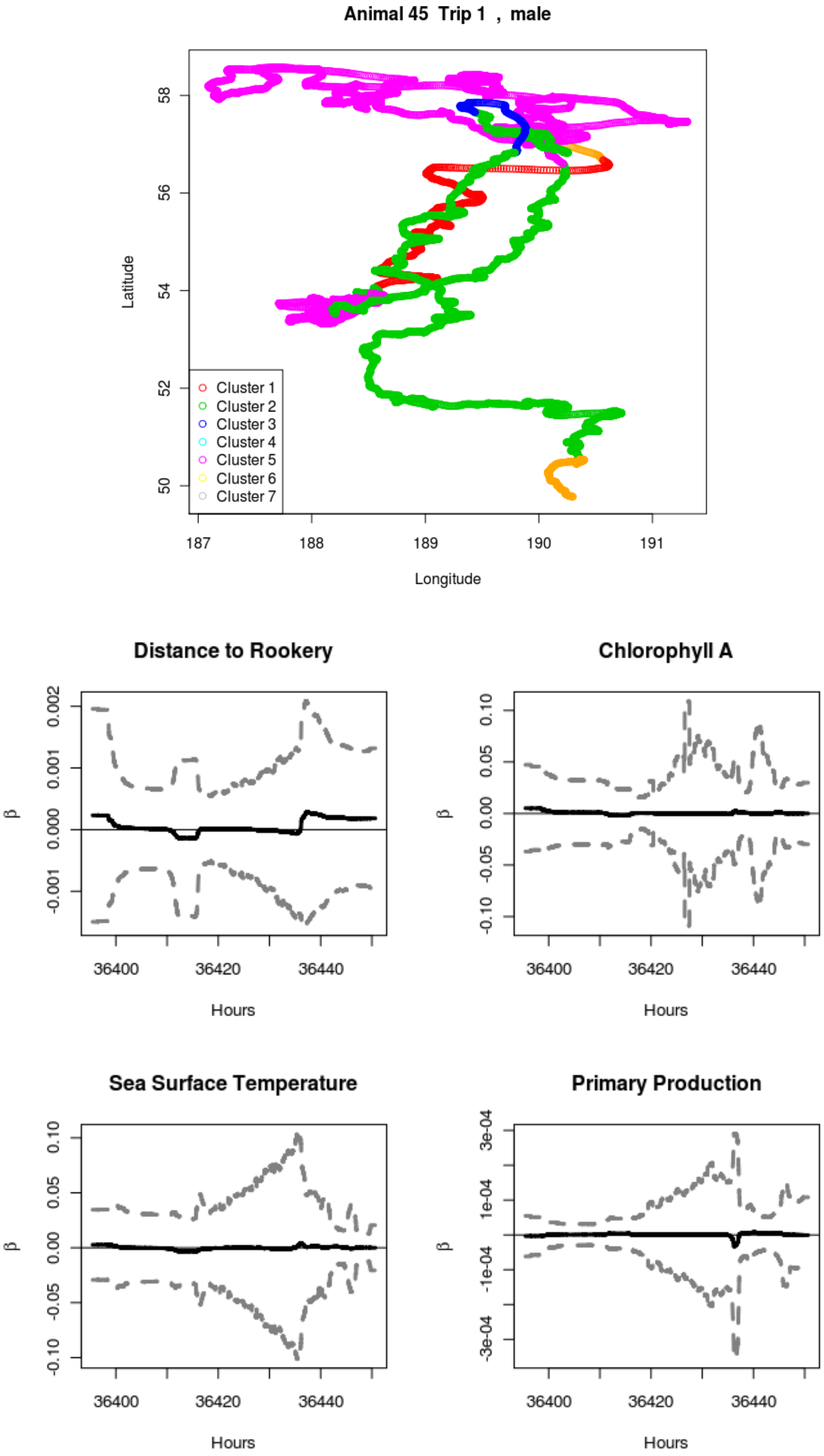
Animal 45 Trip 1 , male
Latitude
Longitude
Cluster 1
Cluster 2
Cluster 3
Cluster 4
Cluster 5
Cluster 6
Cluster 7
Distance to Rookery
Chlorophyll A
Sea Surface Temperature
Primary Production
β
Hours

3 Trace Plots

To assess convergence of our MCMC algorithm, we examined trace plots. We show trace plots from Animal 1 that exhibit convergence. Trace plots for 4 out of the 50 at-sea paths in our study exhibited slow mixing, and were removed from the analysis for lack of convergence (see Section 3.1 of the manuscript).

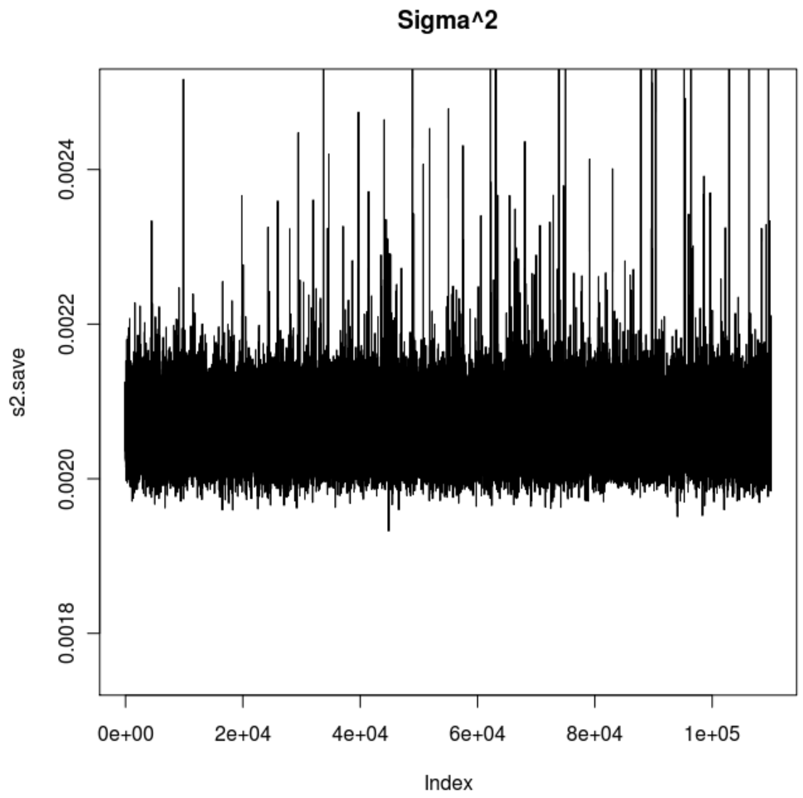

Figure 1. Animal 1 - Trace Plot for σ^2.

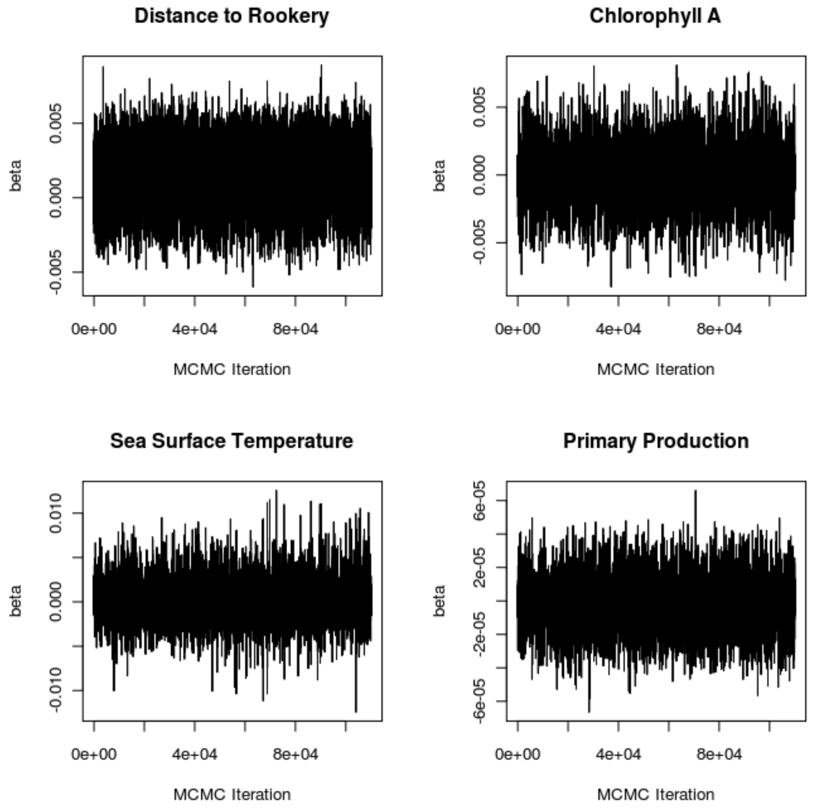

Figure 2. Animal 1 - Trace Plot for $\boldsymbol{\beta}_t$ at $t = 50$ hours.

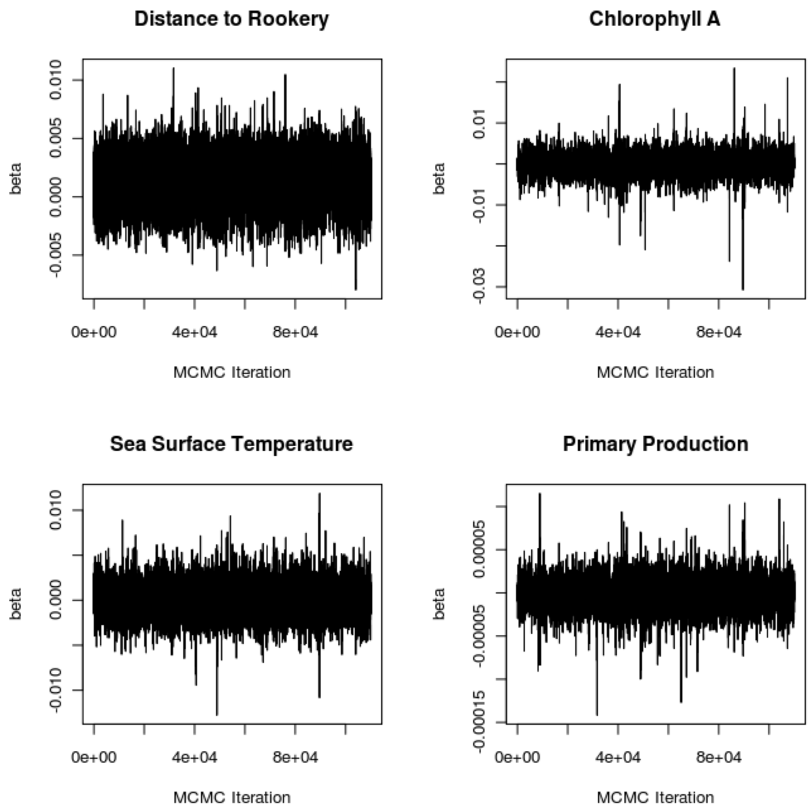

Figure 3. Animal 1 - Trace Plot for $\boldsymbol{\beta}_t$ at $t = 100$ hours.

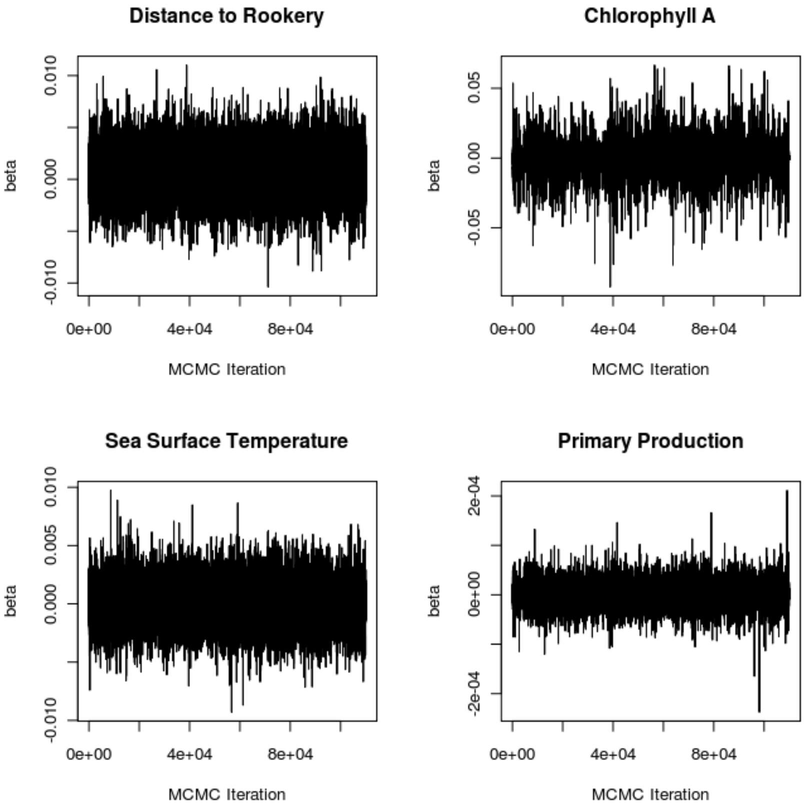

Figure 4. Animal 1 - Trace Plot for $\boldsymbol{\beta}_t$ at $t = 200$ hours.
